# Tuscan Varieties of Sweet Cherry Are Rich Sources of Ursolic and Oleanolic Acid: Protein Modeling Coupled to Targeted Gene Expression and Metabolite Analyses

**DOI:** 10.3390/molecules24081590

**Published:** 2019-04-22

**Authors:** Roberto Berni, Mubasher Zahir Hoque, Sylvain Legay, Giampiero Cai, Khawar Sohail Siddiqui, Jean-Francois Hausman, Christelle M. Andre, Gea Guerriero

**Affiliations:** 1 Department of Life Sciences, University of Siena, via P.A. Mattioli 4, 53100 Siena, Italy; berni10@student.unisi.it (R.B.); giampiero.cai@unisi.it (G.C.); 2 Trees and Timber Institute, National Research Council of Italy (CNR-IVALSA), via Aurelia 49, 58022 Follonica (GR), Italy; 3 Bio-Bio-1 Research Foundation, Sangskriti Bikash Kendra Bhaban, 1/E/1 Poribagh, Dhaka 1000, Bangladesh; mubasher_zahir856@hotmail.com; 4 Life Sciences Department, King Fahd University of Petroleum and Minerals (KFUPM), Dhahran 31261, Saudi Arabia; 5 Environmental Research and Innovation Department, Luxembourg Institute of Science and Technology, 5 avenue des Hauts-Fourneaux, L-4362 Esch/Alzette, Luxembourg; sylvain.legay@list.lu (S.L.); jean-francois.hausman@list.lu (J.-F.H.); andre.christellem@gmail.com (C.M.A.)

**Keywords:** *Prunus avium* L., Tuscan varieties, pentacyclic triterpenes, oxidosqualene cyclase, cytochrome P450, gene expression, bioinformatics

## Abstract

The potential of six ancient Tuscan sweet cherry (*Prunus avium* L.) varieties as a source of health-promoting pentacyclic triterpenes is here evaluated by means of a targeted gene expression and metabolite analysis. By using a sequence homology criterion, we identify five oxidosqualene cyclase genes (*OSC*s) and three cytochrome P450s (*CYP85*s) that are putatively involved in the triterpene production pathway in sweet cherries. We performed 3D structure prediction and induced-fit docking using cation intermediates and reaction products for some OSCs to predict their function. We show that the Tuscan varieties have different amounts of ursolic and oleanolic acids and that these variations are related to different gene expression profiles. This study stresses the interest of valorizing ancient fruits as alternative sources of functional molecules with nutraceutical value. It also provides information on sweet cherry triterpene biosynthetic genes, which could be the object of follow-up functional studies.

## 1. Introduction

The fruits of *Prunus avium* L. are rich sources of health-promoting compounds [1,2,3]. Among the beneficial phytochemicals are polyphenols [4,5] and triterpenes [6]. The latter compose the cuticle of sweet cherry fruits and, more specifically, they are found almost exclusively associated with the intracuticular waxes [7,8]. Triterpenes attract a lot of interest in the nutraceutical and pharmaceutical sectors, in virtue of their beneficial impact on human health [9]. 

The consumption of triterpene-rich fruits and vegetables has indeed a positive correlation with the prevention/decreased susceptibility to develop chronic diseases [7]. Among triterpenes, pentacyclic triterpenes (and their derivatives) are produced and commercialized as dietary supplements or therapeutic agents [9].

The enzymes involved in the synthesis of the three major classes of plant pentacyclic triterpene acids (ursolic, oleanolic and betulinic acid deriving from α-amyrin, β-amyrin and lupeol, respectively) and their structural modifications are the oxidosqualene cyclases (*OSC*s, or triterpene synthases) and cytochrome P450 (*CYP85*s) [10,11]. Numerous OSCs have been characterized in plants but, to the best of our knowledge, no information is available on those from sweet cherries. Indeed, only limited knowledge is available regarding the triterpene profile of sweet cherry fruits. Two studies reported that the major pentacyclic triterpene acids found in the cuticle of sweet cherry fruits at 85 DAFB (days after full bloom) are ursolic (60%) and oleanolic acid (7.5%) [6], with concentrations varying according to the cultivars. Peschel et al. (2007) compared four cultivars, i.e., ‘Hedelfinger’, ‘Kordia’, ‘Sam’ and ‘Van’, and reported no qualitative differences in terms of wax-associated components, but highlighted a lower amount of triterpenes in the waxes of the cv. ‘Hedelfinger’ [6]. Additionally, ursolic acid was reported to be the most abundant triterpene in cold-stored sweet cherry fruits of the cv. ‘Celeste’ and ‘Somerset’ and to increase upon cold storage in the former [12].

Italy is an important producer of sweet cherry fruits, with a stable production of around 110,000–120,000 tons over ca. 30,000 ha of orchards [13]. At the regional level, Italy has started conservation programs aimed at preserving the local biodiversity via *ex situ* collections; in this respect Tuscany is at the forefront, with a regional law (law 64/04; [14]) created for the recovery and preservation of local varieties of woody and herbaceous plant species [15]. These varieties are reported in the Regional Bank of Germplasm, which provides, when possible, both phenotypic and genotypic information [16]. All of the reported plant biodiversity comprises ancient varieties, which designate plants that were grown in the past, but that have not been subject to a market-driven pressure, thus falling out of agricultural interest. These varieties can thrive in soils where the human input is minimal and are sources of interesting agronomic characters, notably higher resilience to adverse environmental conditions.

Sustainable agricultural practices based on the valorization of the local biodiversity help prevent habitat loss due to urbanization. Additionally, they provide a boost to the local economy, by promoting the commercialization of products deriving from regional varieties, thereby using a “0 km” concept that reduces the C footprint. The local agrobiodiversity comprises crops (woody and herbaceous), as well as indigenous soil microbiota and its valorization contributes, on a wider perspective, to ecological restoration, i.e., the recovery of the local ecosystem. Such an awareness of the importance that local bioresources have is already reflected in research projects involving local farmers, enterprises and universities and aimed at promoting fully traceable products, from the raw material to the final product [17].

With a view to valorize non-commercial regional plant varieties of Tuscany, we have analyzed the content of ursolic and oleanolic acid in six ancient *P. avium* varieties. We show different levels of pentacyclic triterpenes in the fruits of Tuscan varieties. Furthermore, we identify five *OSC*s and two *CYP85*s that are putatively implicated in the sweet cherry triterpene biosynthetic pathway and analyze their expression in the six varieties at the stage commercial harvest (ca. 60 dpa, days post anthesis). Bioinformatics carried out to perform 3D structure prediction and induced-fit docking of cation intermediates, as well as of products from three sweet cherry OSCs complement the analyses.

Our results highlight the significant nutraceutical value of ancient Tuscan sweet cherries. On a longer-term perspective, this study sensitizes to the preservation and study of ancient local plant varieties.

## 2. Results and Discussion

### 2.1. Bioinformatics

NCBI BLAST searches of the apple OSC orthologs [10,11] (nucleotide sequences) in the sweet cherry genome [18] led to the identification of five transcripts coding for XP_021819927.1, XP_021819916.1, XP_021819911.1, XP_021810674.1, XP_021819928.1 (Supplementary Information). Of the corresponding protein sequences, three are full length putative OSCs, XP_021819911.1, XP_021819928.1 and XP_021819927.1. NCBI BLAST of the apple *CYP716A175,* a known triterpene C-28 oxidase [11], resulted in three transcripts coding for the full-length proteins, XP_021816761.1, XP_021823674.1 and XP_021815663.1 (Supplementary Information).

**Figure 1:**
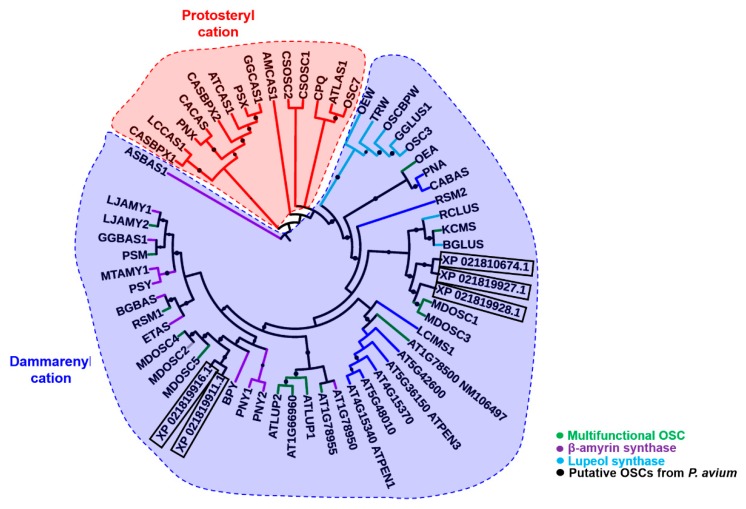
Maximum likelihood phylogenetic tree (number of bootstrap alignments in ultrafast mode: 1000) of different OSC protein sequences (conserved regions only) from the plant species used by Brendolise and colleagues [10] and from sweet cherry (boxed). The branch with MdOSC2 is in gray, as it may represent a non-functional pseudogene. The FASTA protein sequences used to build the tree are provided in Supplementary Information. Boostrap values ranging from 0.8 to 1 are displayed as black circles; the bigger the circle, the higher the bootstrap value.

Alignment of the *P. avium* putative OSCs with those from apple revealed the presence of the conserved sequence QX_3_GXW, together with the substrate-binding motif DCTAE [11,19,20,21] (Figure S1). The maximum likelihood phylogenetic analysis shows that the *P. avium* putative OSCs XP_021819911.1 and XP_021819916.1 branch together with the cluster formed by monofunctional β-amyrin synthases (indicated in violet) and the multifunctional apple MdOSC4 (*Malus domestica* oxidosqualene cyclase) and 5 (indicated in green) (Figure 1). The putative OSCs XP_021810674.1, XP_021819927.1 and XP_021819928.1 cluster instead in the clade formed by multifunctional OSCs and lupeol synthases (Figure 1). From the phylogenetic analysis, we can speculate that the identified OSCs belong to the multifunctional type, given their stricter clustering with apple multifunctional orthologs.

**Figure 2:**
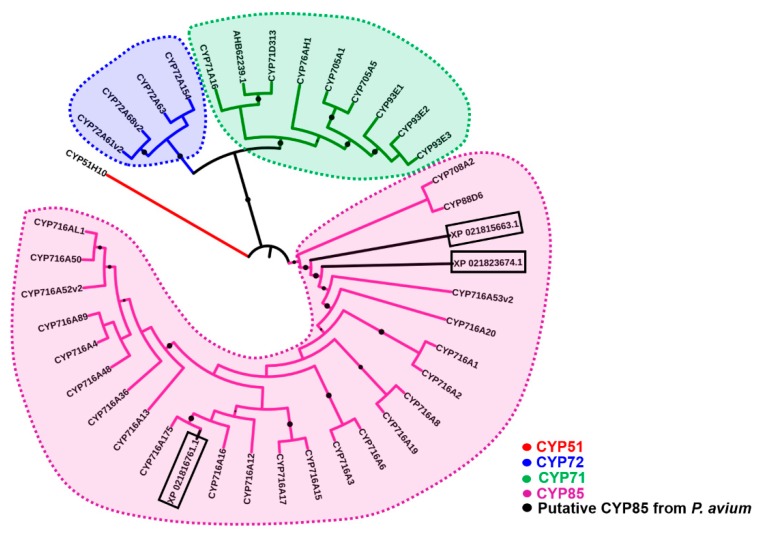
Maximum likelihood phylogenetic tree (number of bootstrap alignments in ultrafast mode: 1000) of different CYP protein sequences (conserved regions only) from the plant species used by Andre and colleagues [11] and from sweet cherry (boxed). The FASTA protein sequences used to build the tree are provided in Supplementary Information. Bootstrap values ranging from 0.8 to 1 are displayed as black circles; the bigger the circle, the higher the bootstrap value.

The phylogenetic analysis of the putative sweet cherry CYPs shows their clustering in the clade composed by CYP85s (Figure 2). XP_021816761.1 clusters together with the recently characterized apple CYP716A175 [11] and is therefore the closest, in terms of sequence, among the three CYP85s found in sweet cherry.

### 2.2. Protein Modeling

**Figure 3:**
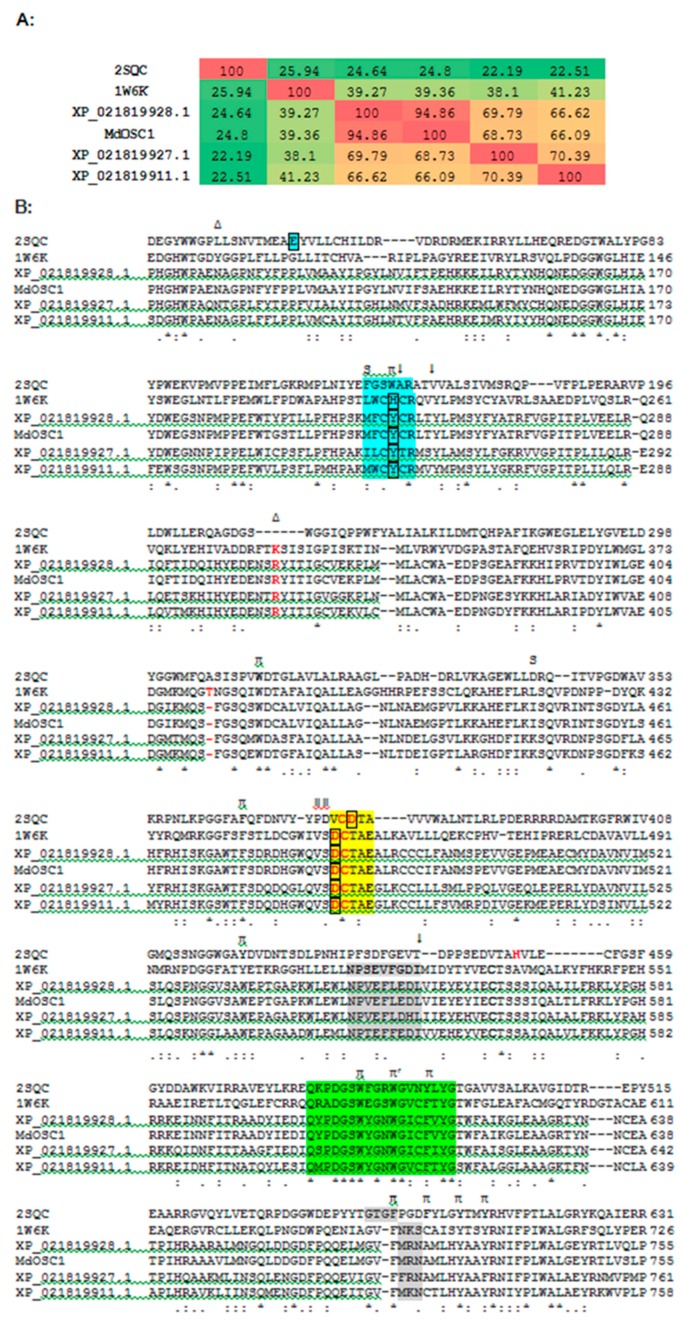
Percentage identity (**A**) and multiple alignment (**B**) of OSCs from fruits, bacteria (2sqc) and human (1w6k). Apple, Cherry9911, 9927 and 9928 indicate MdOSC1, XP_021819911.1, XP_021819927.1 and XP_021819928.1, respectively. Cyan highlighted, conserved M(W/Y)CY(C/S)R motif; yellow highlighted, conserved DCTAE motif; green highlighted, conserved Q-X3-G-X-W-X3-W-G motif; grey highlighted, loops surrounding substrate channel; π, FYWH may be involved in carbocation-pi interactions, whereas π^r^ is used as binding reference in docking experiments; red boxed, Asp involved in the initial protonation of linear substrate; red Cys/His, H-bonding partners of Asp involved in increasing their acidity; cyan boxed, involved in deprotonation of the cyclic substrate at the end; ↓, substrate entry; Δ, may be involved in steric deformation; ǁ, may be involved in correct substrate orientation (Asp in 2SQC; Val in all others); S, product specificity. Unimportant sequence blocks have been removed.

**Figure 4:**
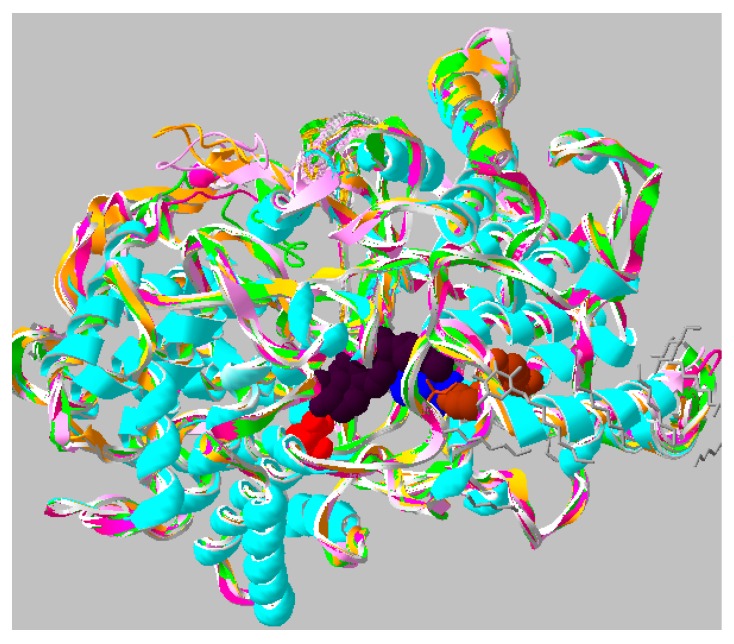
Homology models of fruit OSCs superimposed on the human crystal structure (1w6k). Cyan, human; yellow, orange and pink, XP_021819911.1, XP_021819927.1 and XP_021819928.1; green, MdOSC1. The product, along with important residues, are shown only for human OSC. Black, lanosterol; blue, H232; brown, substrate entry point (C233, Y237, I524); red, active site D455 involved in protonation of oxidosqualene substrate. Grey, β-octylglucoside showing enzyme regions interacting with hydrophobic membrane-inserting surface.

**Figure 5:**
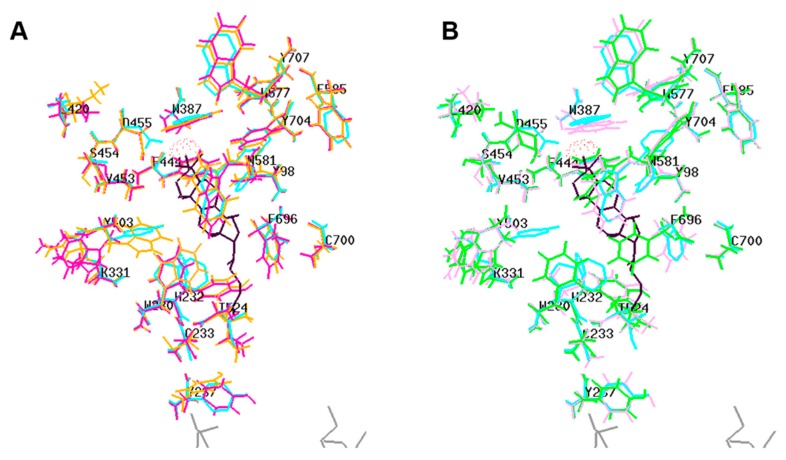
Critical residues of human OSC crystal structure (PDB, 1w6k) superimposed on fruit models. **A:** human (cyan); XP_021819911.1 (orange) and XP_021819928.1 (dark pink). **B:** XP_021819927.1 (light pink) and apple MdOSC1 (green). The ligand (black) is lanosterol. The red dots correspond to hydroxyl group in the substrate that makes H-bond with the catalytic Asp residues (D455 in human). Readers are referred to Figure 4 for a close-up of the interaction between D455 and the ligand. The residue numbering is as in human OSC. Grey, β-octylglucoside showing enzyme region interacting with hydrophobic membrane-inserting surface. The reader should refer to legend of Figure 1B (multiple alignment) for the significance of each residue.

In the absence of a plant OSC X-ray structure, fruit (cherry and apple) enzymes were modeled on the human template (PDB 1w6k). Our results indicate that the sequence alignment (Figure 3), overall protein fold (Figure 4) and ligand interacting residues (Figure 5A,B) of fruit enzymes are very similar to the human OSC that produces tetracyclic products, rather than to the bacterial enzyme (1ump) that produces pentacyclic products.

Interestingly, the percentage identity within all three cherry isoforms ranges from 66–70%, whereas the identity between cherry (XP_021819928.1) and apple (MdOSC1) was 95%. The percentage identity of fruit OSCs with the bacterial and human ones ranges from 22–25% and 38–41%, respectively (Figure 3A).

A Tm-score > 0.5 indicates a model of correct topology. The Tm-scores and root-mean-square deviation of atomic positions (RMSD) of fruit models using the human template (1w6k) range from 0.93–0.97 and 0.64–1.08 Å, respectively, whereas Tm-scores and RMSD of fruit models using bacterial squalene-hopene cyclase template (1ump, 2sqc) are around 0.75 and 2.9 Å, respectively. Based on the higher sequence identity (Figure 3A) and modeling scores (Tm-score and RMSD) of fruit OSCs with the human template (1w6k), rather than with the bacterial squalene-hopene cyclase (1ump, 2sqc), all the modeling was carried out using the human OSC template. The quality of fruit models based on the Ramachandran plots (not shown) indicate that for all cherry and apple models, 93.1–94.6 and 97% respectively of the residues are found in favored + allowed regions. More importantly, none of the ligand-interacting and catalytically critical residues shown in Figure 3B and Figure 5 are located in the outlier region for any of the fruit models.

The multiple alignment shows various conserved motifs and residues involved in the ligand interaction in fruit, bacterial and human OSCs (Figure 3B). The catalytic Asp (D455 in the human enzyme and equivalent residues in the fruit ones) involved in the protonation of oxidosqualene to form a hydroxyl group is structurally conserved in all fruit and human OSCs (Figure 3B, red boxed; Figure 4, red residue; Figure 5). In the bacterial OSC, it is shifted two residues towards the C-terminus (Figure 3B). Another important catalytic residue is His232 in human OSC (Figure 3B, cyan boxed; Figure 4, blue residue; Figure 5, H232 and equivalent Tyr residues in fruits) that is involved in the deprotonation of the cyclic substrate at the end. While this role has been proposed to be performed by a Glu and/or water molecule in the bacterial OSC [22] (Figure 3B, cyan boxed), it is interesting that in fruit OSCs H232 has been replaced by Tyr residues (Figure 3B, cyan boxed; Figure 5). The pKa of the side chain of Tyr residues is ~10.5, which is expected to further increase inside the hydrophobic active site. This results in the inability of Tyr to be ionized at physiological pH and deprotonate the cation intermediate, thus leading to pentacyclic (lupeol, α- and/or β-amyrin), rather than tetracyclic products, such as cycloartenol and lanosterol [22,23,24]. Conserved aromatic residues involved in stabilizing intermediate cations by cation-pi interactions are also shown, with the noteworthy exception that F605 in bacterial OSC is replaced with Cys or Ala residues in plant and fruit enzymes (Figure 3B). W169 and F605 stabilize the cation intermediate required for the cyclization of the 5th ring in bacterial enzyme [24]. Although W169 is replaced by Tyr residues in fruits, there is no aromatic residue in lieu of F605 in fruit OSCs (Figure 3B).

Minor changes in amino acids have been implicated in major changes in the type of products formed by OSCs [25]. For example, Trp within MWCYCR motif (as in XP_021819911.1) has been linked to β-amyrin specificity, whereas Leu (as in XP_021819927.1) and Phe (in XP_021819928.1 and MdOSC1) at this position are implicated in lupeol synthesis [10] (Figure 3B). Similarly, Lys residue (K449 in XP_021819911.1, K452 in XP_021819927.1) have been implicated in α/β-amyrin specificity, whereas Ala, Asn or Ile (I448 in XP_021819928.1 and MdOSC1) have been proposed to be involved in lupeol activity [10]. The mutation of Ser or Thr (equivalent to S699 in human) to Phe in plants has been shown to convert OSC to make tetracyclic rather than pentacyclic products [20]. However, these rules are not unambiguous, as all fruit OSCs are supposed to be amyrin producers based on Lys and Ile residues, but should be lupeol synthases based on MFCYCR/ILCYCR motifs in XP_021819927.1, XP_021819928.1 and MdOSC1. This implies that these fruit enzymes are either multifunctional OSCs (as the phylogenetic analysis shown before would suggest, Figure 1), or other residues may be involved in the product specificity [10,25]. It is noteworthy that MdOSC1, which shows 95% identity with XP_021819928.1, has been proposed to be a mixed amyrin synthase [10], able to produce α-amyrin, β-amyrin and lupeol with a ratio 85:13:2 [11].

**Figure 6:**
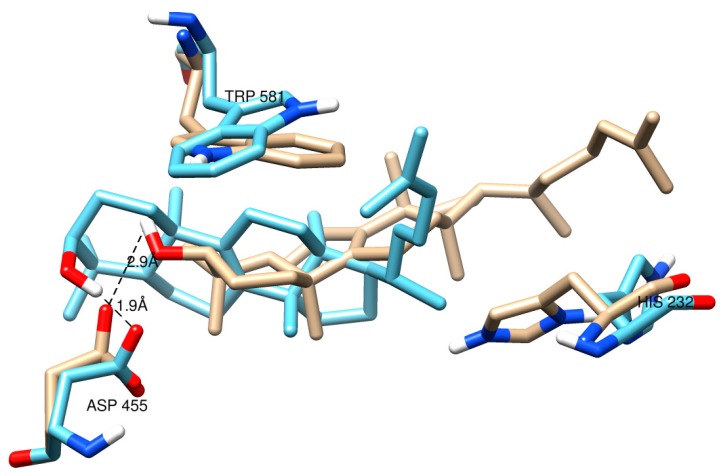
Validity of docking using ROSIE (Rosetta Online Server that Includes Everyone) Ligand Docking. Lanosterol was docked using the human OSC crystal structure (1w6k) without the ligand and then superimposed on 1w6k-lanosterol complex using CZ3 atom of W581 as the docking center. Brown, ligand pose in the crystal structure showing oxygen atom (red) of hydroxyl group; cyan, ligand docked using ROSIE. Hydrogen bonds between lanosterol and D455 (involved in initial protonation of the substrate) are shown for both structures. The deprotonating H232 (lower right) is also shown.

Although sequence comparisons give useful insight into the functions of OSCs, it cannot accurately predict the product specificity. In order to identify which products could possibly be formed by sweet cherry OSCs, we subjected human and fruit structures to docking analysis using intermediate cations [10,23]. Enzymes bind high-energy intermediate states much better than ground-state substrates or products and can help predict the structures of products [26]. In order to validate docking, we compared the lanosterol-complexed human OSC (1w6k) with the OSC structure docked with the same ligand. The result shows that the docking of lanosterol (ligand-OH….OOC-Asp distance, 1.9 Å) mimics the pose of the ligand, as obtained by X-ray crystallography (ligand-OH….OOC-Asp distance, 2.9 Å). Both are within the allowed H-bond distance (2–4 Å), except the linear chain with rotatable bonds where the docked ligand shows different orientation (Figure 6).

**Figure 7:**
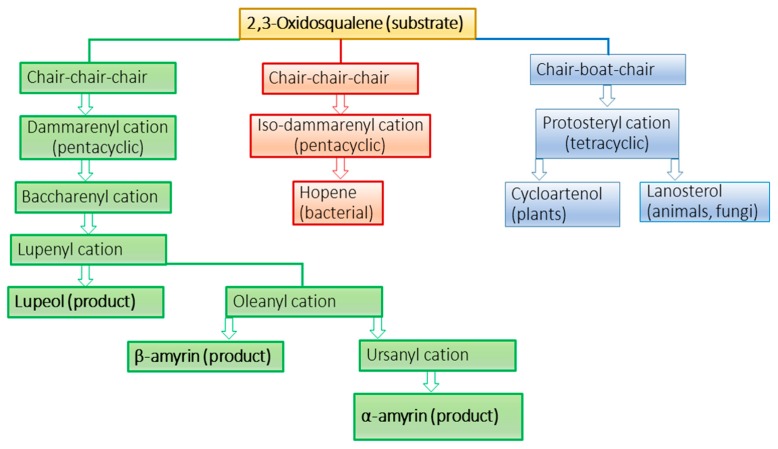
Conversion of substrate to various products via cation intermediates in fruits compared to that in other organisms, according to the previous literature [10,20,28]. The three main routes are colored green, pink and blue based on various triterpene skeletons. Products of OSCs (amyrins) can subsequently be converted to oleanolic and ursolic acids by C-28 oxidases.

Product specificity in OSCs has been proposed to be achieved by enforcing 2,3-oxidosqualene to bind the hydrophobic active site in either chair-chair-chair (CCC) or chair-boat-chair (CBC) conformations leading to partially cyclized intermediate cations that are stabilized by aromatic residues via cation-pi interactions [27]. The CCC and CBC forms lead to various products via cation intermediates (Figure 7) that prevent early termination of the cyclization process. The termination of cyclization in humans occurs by deprotonation by His232 and in bacteria by Glu45 [27] (Figure 3B), or water molecule. However, corresponding His or Glu residues are missing in cherry and MdOSC1 enzymes (Figure 3B). The docking analysis (distance between ligand-OH and O atom of catalytic Asp and protein-ligand affinities) of OSCs with various cation intermediates, such as protosteryl, iso-dammarenyl and dammarenyl is expected to discriminate between various pathways leading to different products in human, bacterial and fruit enzymes (Figure 7).

Previously, we successfully used the Mcule tool to analyze the docking of various substrates in laccases from Tuscan sweet cherry varieties [29]. However, compared to the accessible active site of laccases, OSCs have a more hydrophobic and closed catalytic pocket (Figure 4). Docking of the cation intermediates using the advanced Mcule version showed incorrect poses and low affinities for all, but a couple of OSC–ligand complexes, where the -OH group of the ligand either does not face the O atom of catalytic Asp, or displayed very large ligand-OH…OOC-Asp-protein distances (results not shown). The inability to dock in the correct pose with Mcule (-OH of the ligand able to form a hydrogen bond within a distance of 1.5–4 Å with the oxygen atom of catalytic Asp residue) [24,28] and the low ligand-OSC affinities are probably either due to the high number of rotatable bonds giving alternative catalytically non-competent orientations [26], and/or the inability of the protein backbone to undergo large movements (free-energy of orientation) upon binding of cation intermediates [30]. It is noteworthy that the cyclization reaction (where double bonds are replaced by single bonds) is highly exothermic [27] and this heat can be exploited for the conformational changes in both the ligand and the active site [22].

Recent work in *Ononis spinosa* OSC found that by using 2,3-oxidosqualene dioxide with epoxide groups on either end, the hydroxylated intermediate is able to re-enter the active site for hydroxylation of the second epoxide ring to give α-onocerin, implying conformational changes in the active site to accommodate the intermediate [31]. Protein flexibility is very crucial, as it can compensate for errors in the homology models, as well as allow ligands to bind to the active site [28]. Some workers have got a higher level of correct poses due to the application of strict restraints, such as H-bond between the ligand and catalytic Asp as in OSCs [28], or molecular dynamics simulation to allow conformational changes in the protein molecule [30]. However, putting H-bond restraint will preclude from getting realistic poses, variable ligand to OSC distances and binding affinities.

**Figure 8:**
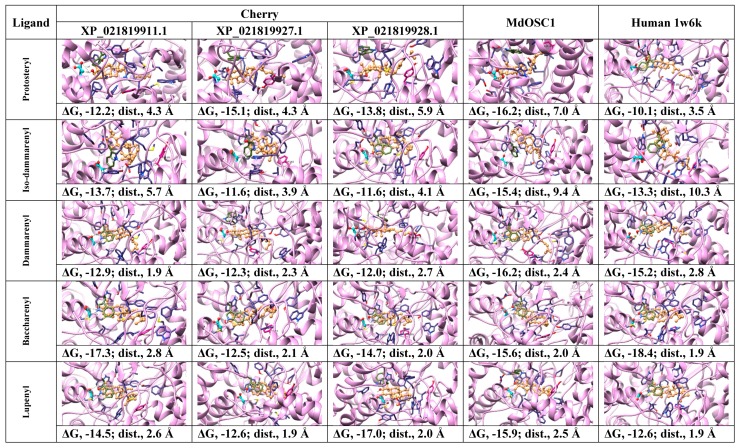

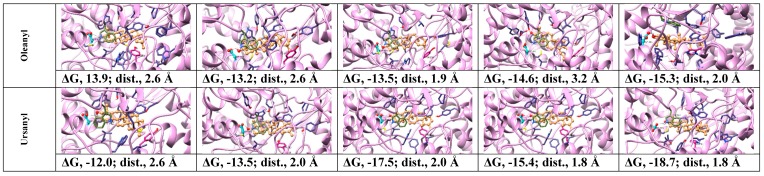
Poses, ligand to protein distances and affinities of oxidosqualene cyclases (OSCs) docked with the intermediate cations from fruit models and human X-ray structure. ΔG, Gibbs free-energy of binding (kcal mol^−1^); dist, ligand-OH.…OOC-Asp-protein distances, ligand, brown; catalytic Asp, cyan; Trp docking reference, green; Tyr/His: magenta. Residues surrounding the active site, dark blue; oxygen atoms, red; nitrogen atoms, light blue and hydrogens, white.

In order to assess if induced-fit docking of cation intermediates can improve the ligand-OH…..OOC-Asp distances and protein-ligand affinities, all OSCs were subjected to docking analysis using ROSIE Ligand Docking (RLD) tool (Figure 8). In contrast to Mcule that considers the ligand and protein side-chains as flexible and the protein backbone as rigid, RLD additionally takes into account the backbone flexibility of the active site, thus improving the ligand–protein fit via induced-fit mechanism [32].

**Figure 9:**
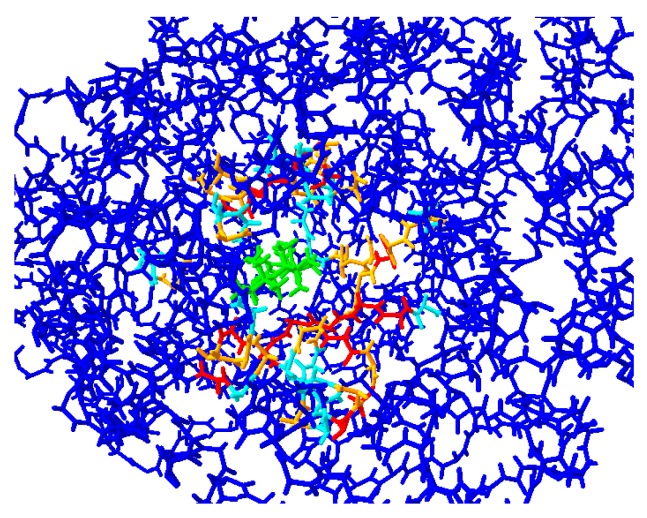
Cherry XP_021819928.1 model was superimposed on its docked structure with dammarenyl cation for the determination of backbone RMSD of all residues due to induced-fit docking. Blue, 0 RMSD; cyan, RMSD between 0.05–0.1 Å; orange, RMSD between 0.1–0.2 Å; red, RMSD > 0.2 Å. Dammarenyl cation ligand is shown in green.

In order to validate that RLD is taking into account the flexibility of the active site during ligand docking, we determined the RMSD of the overall structures and key residues surrounding the catalytic-site. The overall RMSD (spread over the entire protein molecule) of all OSCs docked with cation intermediates and products varied from 0.04–0.076 Å, whereas RMSD of residues away from the active site were close to 0 Å and RMSD of residues surrounding the active site were > 0 Å (results not shown). For example, the RMSD values of the residues surrounding the catalytic site of a representative docked cation-intermediate and a product are given in Table 1, whereas a color representation of cherry XP_021819928.1 docked with dammarenyl cation is shown in Figure 9. The RMSD of residues away from the active site were close to 0 Å (Figure 9), whereas the RMSD values of residues surrounding the catalytic-site varied from 0.102–1.245 Å (Table 1), thereby confirming that backbone conformational changes within the active site took place upon docking. 

The docking poses, ligand to Asp distances and OSC–ligand binding affinities are given in Figure 8. The results indicate that for protosteryl cation, only human OSC gave the distance between the ligand and catalytic Asp within the hydrogen bond distance (3.5 Å, Figure 8), whereas none of the distances in fruit OSCs were within the hydrogen bond distances. This indicates that fruit OSC do not synthesize tetracyclic products via protosteryl cation route (Figure 6). For iso-dammarenyl cation, the ligand-Asp distances were large for all OSCs, except for cherry XP_021819927.1 and XP_021819928.1, which were borderline (~4 Å), with relatively low ligand–OSC affinities (Figure 8) implying that fruit and human enzymes do not likely form hopene-type products (Figure 6). For all other intermediates, leading from dammarenyl to ursanyl cations, docking showed very short hydrogen bond distances and high ligand to protein affinities (Figure 8) implying that fruit OSCs likely forms lupeol, α-amyrin and β-amyrin products (Figure 6). The likely reason that human OSC binds these intermediates with short hydrogen bonds and high affinities (Figure 8) implies a flexible active site that can undergo conformational change to accommodate these cations. In spite of very good binding between these cations and human enzyme, the formation of four-ringed lanosterol product in human OSC is due to the active site His232 that deprotonoates the cation intermediate thus terminating the reaction at the tetracyclic stage [24].

**Figure 10:**
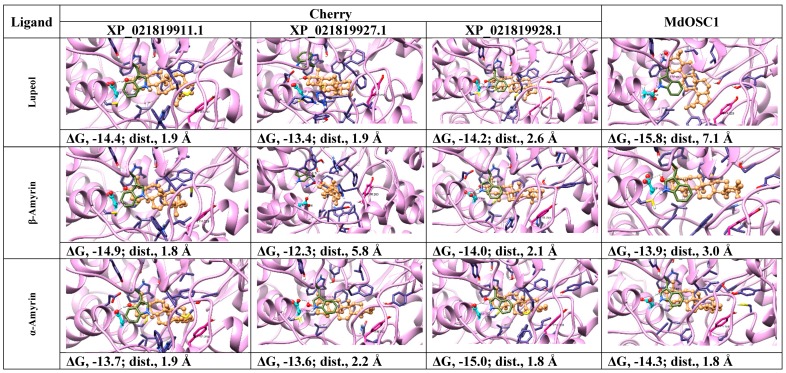
Poses, ligand-OH----OOC-Asp-protein distances and affinities of oxidosqualene cyclases (OSCs) docked with the reaction products from fruit models. ΔG, Gibbs free-energy of binding (kcal mol^−1^); dist, ligand-OH.…OOC-Asp-protein distances. Ligand, brown; catalytic Asp, cyan; Trp docking reference, green; Tyr/His: magenta. Other active site residues, dark blue; oxygen atoms, red; nitrogen atom, light blue and hydrogens, white.

In order to discriminate between three possible types of products (lupeol, α-amyrin and β-amyrin) formed by the Tuscan sweet cherry OSCs, we subjected fruit OSCs to docking analysis using products (Figure 10) along with lanosterol and human OSC as a control (not shown). While tetracyclic lanosterol binds well to sweet cherry XP_021819911.1 and MdOSC1, the minimum ligand to Asp distance (1.9 Å) was obtained with human OSC (results not shown) implying that lanosterol is the preferred substrate for human enzyme. Lupeol binds to all cherry enzymes with low ligand to Asp distances (1.9–2.6 Å) and high ligand–protein affinities (−13.4 to −14.2 kcal mol^−1^), compared to apple MdOSC1 that showed larger distance (Figure 10), implying that all cherry OSCs have correct active-site topology to synthesize lupeol. Similarly, Figure 10 shows that all fruit OSCs also have potential to form α-amyrin, as well as β-amyrin, except cherry XP_021819927.1.

Based on the present docking results employing cation intermediates and products, all cherry OSCs are competent to produce all three products (lupeol, α-amyrin, β-amyrin), except XP_021819927.1 that is unable to make β-amyrin (Figure 8 and Figure 10). Based on the above-described role of amino acids in product specificity (Figure 3B), it can be predicted that sweet cherry XP_021819928.1 is likely to be a lupeol synthase, XP_021819927.1 a mixed amyrin/lupeol synthase and XP_021819911.1 an amyrin-synthesizing OSC. Collectively, the results obtained via bioinformatics imply that the OSCs from the Tuscan sweet cherries are able to synthesize a mixture of all three products; this awaits experimental confirmation, as these enzymes may each show capability to produce all the products in different ratios, as shown experimentally for MdOSC1 [11].

### 2.3. Gene Expression Analysis

The primers designed on the genes encoding the OSC XP_021810674.1 and the CYP85 XP_021815663.1 did not amplify with suitable efficiencies (comprised between 90–110%); they were therefore discarded and not included in the gene expression study.

**Figure 11:**
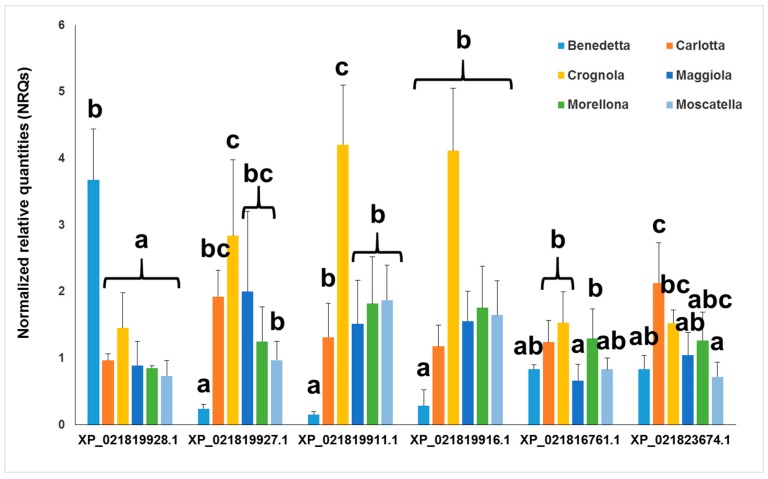
Gene expression analysis of the putative *OSC*s and *CYP85*s in the ancient sweet cherry varieties. For linearity with the previous phylogenetic data, the accession numbers of the corresponding proteins are used to identify the *OSC*s and *CYP85*s. Error bars indicate standard deviations (*n* = 4). Different letters indicate statistically significant differences (*p* < 0.05) calculated with the Tukey’s post-hoc test.

The expression analysis highlights different values among the Tuscan varieties (Figure 11). ‘Crognola’ and ‘Carlotta’ are among the varieties showing the highest expression levels of *OSC*s and *CYP85*s. This finding is very interesting if one considers the recently reported higher content of phenolic compounds in these ancient Italian sweet cherries [33] and the higher expression levels of genes encoding laccase-like multicopper oxidases [29]. ‘Benedetta’ shows higher levels than all the other varieties only for XP_021819928.1. As discussed in the next paragraph, for the sake of comparison we also included a commercial variety (‘Durone’, purchased at a local grocery shop in Siena, Tuscany), which displayed lower expression levels (not shown). The data for the commercial variety are not given because of the different storage conditions of the fruits: the post-harvest storage may indeed have had an effect on the endogenous mRNA concentrations, thereby affecting the expression levels. The results here shown on the expression levels of *OSC*s and *CYP*s suggest higher levels of pentacyclic triterpenes. It was therefore necessary to quantify their amounts in the ancient fruits from Tuscany.

### 2.4. Pentacyclic Triterpene Content

**Figure 12:**
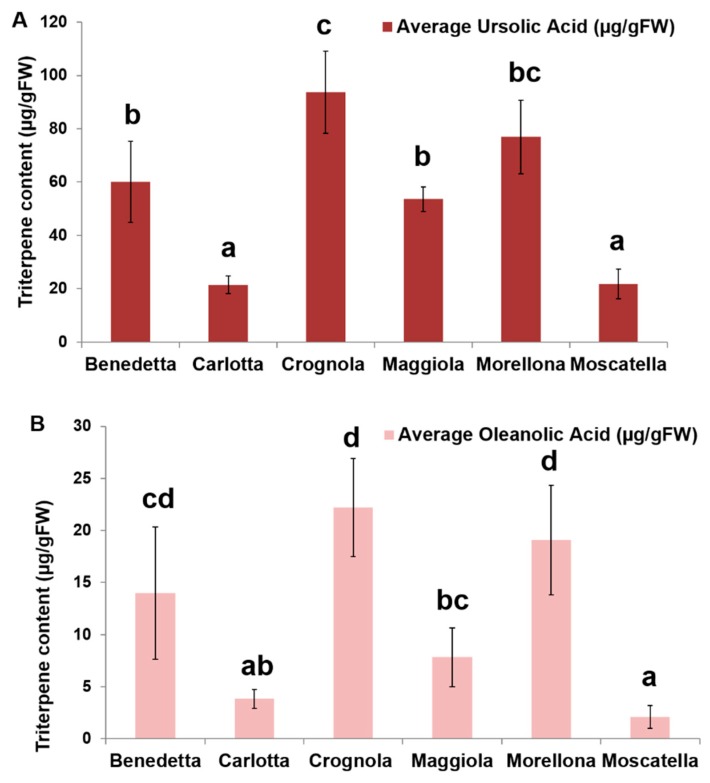
Triterpene composition of the Tuscan sweet cherry fruits. **A**: ursolic acid content; **B**: oleanolic acid content. Data are expressed in µg g^−1^ FW (*n* = 5, with the exception of ‘Crognola’, where *n* = 4). Different letters indicate statistically significant differences (*p* < 0.05) calculated with the Tukey’s post-hoc test.

The quantification of the pentacyclic triterpenes from the Tuscan sweet cherries indicates a higher content of ursolic acid, as compared to oleanolic acid (Figure 12). No betulinic acid is found in the fruits. This is in agreement with what previously reported in the literature, where a higher % w/w was quantified in *P. avium* fruits [6]. The average content of ursolic acid is significantly higher in some of the Tuscan cherries; in particular, the varieties ‘Crognola’ and ‘Morellona’ show the highest contents, followed by ‘Maggiola’, ‘Benedetta’, ‘Carlotta’ and ‘Moscatella’. The same ranking in terms of average oleanolic acid content is observed among the ancient varieties: ‘Crognola’ and ‘Morellona’ show the highest contents, followed by ‘Benedetta’, ‘Maggiola’, ‘Carlotta’ and ‘Moscatella’. We also determined the concentrations of ursolic and oleanolic acid in the commercial variety. The commercial fruits displayed significantly lower contents (7-fold and 31-fold lower than ‘Crognola’ for ursolic and oleanolic acid, respectively). We aimed at making a first comparison to confirm the nutraceutical value of the ancient fruits. Future studies on the commercial fruits performed under the same sampling conditions here described will confirm these preliminary data. It is interesting to note that post-harvest studies on two commercial varieties showed very little variation (1—10%) in both ursolic and oleanolic content after separate (20 °C, 3 days and 0 °C, 14 days), as well as combined (20 °C + 0 °C, 17 days) storage [12].

The gene expression analysis and targeted metabolomics here performed show varying levels of the pentacyclic triterpenes ursolic and oleanolic acids in the ancient Tuscan fruits. We identify high producing varieties, such as ‘Crognola’ and ‘Morellona’ and our preliminary comparison with a widespread commercial counterpart indicates higher levels of triterpenes in the ancient fruits. This result should however be confirmed by studies carried out on commercial fruits sampled under the same conditions here reported for the Tuscan varieties. Our data confirm the value of ancient local varieties of fruit-trees as alternative sources of secondary metabolites of pharmacological and nutraceutical interest.

## 3. Materials and Methods

### 3.1. Fruit Harvesting

The sweet cherry fruits used in this study were sampled in 2017, as previously described [29], from eighteen-year-old cherry trees (ancient local varieties of *P. avium* on P-HL-B rootstocks) grown in the experimental field of the CNR in Ivalsa Follonica (GR, Italy, coordinates 42° 55′ 59″ N, 10° 45′ 57″ E). Sampling took place in May 2017. However, the variety ‘Benedetta’ was sampled in May 2016, as the trees gave no fruits in 2017. At the sampling time (between 9:00–10.00 a.m.), the average temperature was of 17–20 °C, 71–74% of humidity in 2016 and 2017, respectively. Twenty sweet cherry fruits (growth stage: 60 dpa) were sampled from each tree to have enough biological replicates (four in this study), each represented by a pool of five fruits. Fruits were sampled from different places on the tree canopy (at around 1.70–1.90 m from the soil), to minimize eventual bias due to variations in solar exposure. Fruits were sampled from the trees with the stems, which was rapidly removed after picking. Then the samples were immediately plunged in liquid nitrogen in tubes, brought to the laboratory and stored at −80 °C in Ziploc bags until processing.

### 3.2. RNA Extraction, Primer Design and Real-Time PCR Data Analysis

The RNA extraction procedure, quality control and quantification, as well as reverse transcription and real-time PCR conditions have been previously detailed [29]. The primers used for the reference genes have been previously published [29], those for *eTIF4E*, *GAPDH*, *OSC*s and *CYP8*5s are indicated in Table 2. They were designed with Primer3Plus (http://www.bioinformatics.nl/cgi-bin/primer3plus/primer3plus.cgi) and checked with OligoAnalyzer 3.1 (http://eu.idtdna.com/calc/analyzer).

The expression values of the *OSC*s and *CYP85*s were calculated with qBase^PLUS^ (version 2.5, Biogazelle, Ghent, Belgium) by using the reference genes indicated by geNorm^PLUS^. Eight reference genes were tested for stability; *PavAct7* and *PaveTIF4E* were identified as sufficient for data normalization when tested together with *PavPP2A*, *PavPolyUbq*, *PavSerThr*, *PavAP4*, *PavGAPDH* and *PavTIP41*. A one-way ANOVA with a Tukey’s post-hoc test was performed on log2 transformed NRQs (Normalized Relative Quantities) by using IBM SPSS Statistics v19.

### 3.3. Bioinformatics

The pair-wise multiple alignment of OSCs and CYP85s to identify conserved residues was carried out with CLUSTAL-Ω (http://www.ebi.ac.uk/Tools/msa/clustalo/) [34]. The alignment was then used to build the maximum likelihood phylogenetic tree using W-IQ-TREE [35] (number of bootstrap alignments in ultrafast mode: 1000), available at http://iqtree.cibiv.univie.ac.at. The tree in Newick format was visualized with iTOL (available at https://itol.embl.de/).

The OSC sequences from human, bacteria and fruits to identify conserved residues and motifs were aligned using CLUSTAL-Ω (http://www.ebi.ac.uk/Tools/msa/clustalo/) [34]. The 3D homology models were generated with the I-TASSER Suite (http://zhanglab.ccmb.med.umich.edu/I-TASSER/) [36] utilizing LOMETS, SPICKER, and TM-align. The models based on human OSC (PDB 1w6k) were then refined using REMO by optimizing the backbone hydrogen-bonding networks and FG-MD by removing the steric clashes and improving the torsion angles. The quality of the models was checked by RAMPAGE [37].

Molecular docking of intermediate cations (protosteryl, dammarenyl, iso-dammarenyl, baccharenyl, lupenyl, oleanyl and ursanyl) and the product (lanosterol, lupeol, α-amyrin and β-amyrin) with OSCs was carried out with the advanced version of online Mcule tool (http://doc.mcule.com/doku.php?id=1clickdocking), based on AutoDock Vina [38] and Ligand Docking Tool [32] based on the Rosetta Online Server that Includes Everyone, ROSIE (https://rosie.graylab.jhu.edu/ligand_docking) [39], using the coordinates of atom CZ3 of Trp581 in 1w6k as the binding center and equivalent CZ3 atoms of conserved Trp residues in all other structures [40]. The SMILES codes of products were imported from PubChem (https://pubchem.ncbi.nlm.nih.gov/). The structures of the cation intermediates were drawn within Mcule tool [10,23]. For ROSIE Ligand Docking (RLD), the structures of the cation intermediates were either drawn in PubChem Sketcher (https://pubchem.ncbi.nlm.nih.gov/edit2/index.html) and the SMILES codes imported into NIH Cactus Online SMILES translator (https://cactus.nci.nih.gov/translate/) developed by National Cancer Institute or the structures were directly drawn in NIH Cactus Online Translator and converted to 3D SDF files with hydrogen atoms added according to standard valences. The overall structures showing critical residues were visualized by superimposing fruit models on the human OSC (PDB 1w6k) with DeepView Swiss-PdbViewer v4.1 (http://www.expasy.org/spdbv/) [41]. Molecular graphics and analyses of OSC structures docked with cation intermediates were performed with UCSF Chimera, developed by the Resource for Biocomputing, Visualization, and Informatics at the University of California, San Francisco [42] available at http://www.rbvi.ucsf.edu/chimera. RMSD of the overall structures and key residues surrounding the active site was determined by PDB viewer after superimposing OSC structures before and after induced-fit docking employing cation intermediates and products.

### 3.4. Extraction and Analysis of Pentacyclic Triterpenes

Sample preparation and HPLC analysis were performed based on the previously described method by Andre and colleagues [11]. Five hundred mg of powdered freeze-dried material (exocarp and ca. 5 mm of the mesocarp tissue) were mixed with 10 mL of ethyl acetate/hexane (50:50 *v*/*v*). The mixture was homogenized using a vortex for 30 s and then shaken for 1 h at room temperature. After centrifugation at 10.000 g for 15 min, the supernatant was collected and evaporated to dryness using a centrifugal vacuum evaporator. The pellet was re-extracted using 10 mL of ethanol/H_2_O (80:20 *v*/*v*) solution, homogenized and shaken for 2 h at room temperature and centrifuged at 10.000*g* for 15 min. The supernatant was collected, combined with the lipophilic dried extract and evaporated to dryness. Triterpenes were re-suspended in 1 mL of EtOH and filtered (0.45 µm) before HPLC analysis. Each cherry sample was analyzed in triplicate (*n* = 3). Extracts were analyzed with a Waters Acquity UPLC system (Waters, Milford, MA, USA) hyphenated to a Diode Array Detector (UPLC-DAD). The separation of the 5 µL aliquot was performed on a reverse-phase Acquity UPLC BEH C18 column (2.1 × 100 mm, 1.7 μm particle size, Waters, Milford, MA, USA). The eluents were 0.05% *o*-phosphoric acid in water (A) and 0.05% *o*-phosphoric acid in methanol (B). The gradient was as follows: 0 min, 75% B; 2 min, 75% B; 16 min, 82% B; 25 min, 100% B; 26.5 min, 100% B; 27 min, 75% B; 30 min, 75% B. The flow rate was of 0.3 mL min^−1^ and the column temperature was 40 °C. Ursolic acid and oleanolic acid were identified by their retention times and spectral data by comparison with standards and they were quantified at 210 nm using five-point calibration curves. Excellent linearity (R^2^ > 0.99) was obtained in the concentration range 100–6.25 µg mL^−1^ for both compounds.

## 4. Conclusions

We identified *OSC*s and *CYP85*s involved in pentacyclic triterpene biosynthesis in sweet cherry and measured their expression in ancient Tuscan varieties. The *in silico* analysis performed on three OSCs suggests that these isozymes can synthesize all the three classes (α- and β-amyrins, lupeol) of pentacyclic triterpenes. The high gene expression levels and the increased contents of oleanolic and ursolic acids indicate that the ancient sweet cherries are rich sources of secondary metabolites.

## Figures and Tables

**Table 1:** RMSD values of some key residues surrounding the active site after the induced-fit docking of a cation intermediate and a product on fruit OSCs.

OSC	Residue	RMSD (Å)	
		Dammarenyl Cation	β-Amyrin Product
**XP_021819911.1**	Y 259	0.368	0.254
	G 414	0.109	0.350
	D 485 (protonating Asp)	0.089	0.105
	D 554	0.242	0.690
	W 612 (binding center)	0.395	0.288
	G 613	0.567	0.361
	W 621	0.384	0.001
	K 730	0.151	0.251
**XP_021819927.1**	Y 263	0.266	0.105
	V 373	0.321	0.001
	G 374	0.318	0.001
	D 488 (protonating Asp)	0.123	0.238
	A 536	0.216	0.409
	W 537	0.041	0.483
	W 615 (binding center)	0.161	0.245
	F 732	0.124	0.336
**XP_021819928.1**	Y 259	0.187	0.132
	C 369	1.245	0.793
	F 473	0.354	0.237
	D 484 (protonating Asp)	0.578	0.144
	A 532	0.301	0.246
	W 611 (binding center)	0.102	0.160
	M 726	0.302	0.305
	H 732	0.554	0.044
**MdOSC1**	Y 259	0.198	0.161
	C 369	0.332	0.503
	E 371	0.388	0.432
	K 372	0.876	0.786
	P 373	0.572	0.020
	F 412	0.250	0.417
	D 484 (protonating Asp)	0.151	0.163
	W 611 (binding center)	0.115	0.214

**Table 2:** List of primers with details of the sequences, amplicon sizes and amplification efficiencies.

Name	Sequence (5′→3′)	Amplicon Size	Amplification Efficiency
PaveTIF4E Fwd	GGCAAAGCCTCGATACAATG	71	1.90
PaveTIF4E Rev	TTGGTTATGGAGAGCGAAGAC		
PavGAPDH Fwd	TATCAAAGCCACAGCCACTG	118	1.88
PavGAPDH Rev	TGCTATTCGGAGAACCAACC		
XP_021819928.1 Fwd	AAGGCAGACATGGGAGTTTG	135	1.94
XP_021819928.1 Rev	ATCTGAAAACGCCAGAGGAG		
XP_021819916.1 Fwd	ATGAGGGTTCAGCTTGATGC	104	2.09
XP_021819916.1 Rev	CCACCGTGATTGTGTGAATG		
XP_021819927.1 Fwd	CGAAGAGTGTTGTCTGCTTAACG	109	2.04
XP_021819927.1 Rev	AAAGAGAATAGTCACCCCCTTG		
XP_021819911.1 Fwd	TGTATTCCCAGCAGAGCATC	74	1.90
XP_021819911.1 Rev	CCCAACCACCATCTTCATTC		
XP_021816761.1 Fwd	TGAGAAAGATGCTCCCCAAC	107	1.90
XP_021816761.1 Rev	CCTGTTTTCCCAACCATCAG		
XP_021823674.1 Fwd	GAGGTTCAAATGGGAAGTGC	96	1.90
XP_021823674.1 Rev	TGACGATGAAGACGAACTGG		

## Supplementary Materials

The following are available online, Figure S1: Alignment of the sweet cherry and apple OSCs showing the conserved DCTAE (green highlight), M(W/Y)CY(C/S)R (cyan highlight), as well as the Q-X3-G-X-W motifs (yellow highlight) [11,18,19,20].
