# Supplementary material for: Tuscan Varieties of Sweet Cherry Are Rich Sources of Ursolic and Oleanolic Acid: Protein Modeling Coupled to Targeted Gene Expression and Metabolite Analyses
Source: Molecules. 2019 Apr 22;24(8):1590. doi: 10.3390/molecules24081590 (PMC6515059; doi:10.3390/molecules24081590)
Supplement: Supplementary file 1 [file molecules-24-01590-s001.zip › molecules-471845-final-SM/molecules-471845-final-Figure S1-SM.pdf]

```
XP_021819911.1      MWKLKVADGG--NDPYIYSTNDFVGRQIFEFDPEAGTPEERAEVEEARLNFYNNRYKVKP     58
XP_021819916.1      ------------------------------------------------------------     0
MdOSC5              MWKLKVADGG--NDPYIYSTNNFVGRQIFKFDPEAGTTEERAEVEEARLHFYNTRYQVKP     58
MdOSC2              MWKLKVADGG--NDPYIYSTNNFVGMQIFEFDPEAGTTEERAEVEEARLHFYNNRYQVKP     58
MdOSC4              MWKLKVADGG--NDPYIYSTNNFVGRQIFEFDPEAGTTEERAEVEEARLHFYNNRYQVKP     58
XP_021819928.1      MWKIKFGEGA--NDPLLYSTNNFHGRQTWEFDPDAGTPEERAEVEEARENFYRNRFKVQP     58
MdOSC1              MWKIKFGEGA--NDPMLFSTNNFHGRQTWEFDPDAGTEEERAEVEAAREHFYQNRFKVQP     58
MdOSC3              MWRIKFGEGA--NDPFLFSTNNFHGRQTWEFDPDAGTEEERAEVEAAREHFYQNRFKVTP     58
XP_021810674.1      ------------------------------------------------------------     0
XP_021819927.1      MWKLKFGAEGENVDPYLFSTNKFVGRQIWEFDPHAGTPQERAEVEEARQNFYNNRYKVKA     60

XP_021819911.1      SGDLIWRMQFLREKNFKQTIPEVKVEDGE-EITYEKATASLRRSVHFFSALQASDGHWPA     117
XP_021819916.1      ------------------------------------------------------------     0
MdOSC5              NSDLLWRMQFLREKNFKQTIPPVKVEDGE-EITYEKATAALRRSVHFFSALQASDGHWPA     117
MdOSC2              SGDLLWRMQFLREKNFKQTIPPFKVEDGE-EITHEKATASLRRSVHFFSALQASDGHWPA     117
MdOSC4              SGDFLWRMQFLREKNFKQTIPPIKVEDGE-EITHEKATASLRRSVHFFSALQASDGHWPA     117
XP_021819928.1      SSDLLWRFQMQRERNFKQEIPPVRIGEGE-EITYDQATAAYRRAATFWNALQSPHGHWPA     117
MdOSC1              SSDLLWRFQILREKNFKQEIPPVRVGEGE-DITYDQATAAFRRAATFWNALQSPHGHWPA     117
MdOSC3              SSDLLWRFQILREKNFQQEIPPVRIGEGE-EITYDQATAAFRRAATFWNALQSPHGHWPA     117
XP_021810674.1      ------------------------------------------------------------     0
XP_021819927.1      SSDILWRMQLHRERNFKQTIPQVKIEDDDEECRHEKATVAFKRAVAFWSALQSPHGHWPA     120

XP_021819911.1      ENAGPLFFLPPLVMCAYITGHLNTVFPAEHRKEIMRYIYYHQNEDGGWGLHIE-GHSTMF     176
XP_021819916.1      ----------------------------------------------------------MF     2
MdOSC5              ENAGPLFFLPPLVMCTYITGHLNTVFPTEHRKEILRYIYYHQNQDGGWGLHIE-GHSTMF     176
MdOSC2              ENAGPLFFLPPLVMCVYITGHLNTVFHAEHRKEILRYIYYHQNQDGGWGLHIE-GHSTMF     176
MdOSC4              ENAGPLFFLPPLVMCVYITGHLNTVFHAEHRKEILRYIYYHQNQDGGWGLHIE-GHSTMF     176
XP_021819928.1      ENAGPNFYFPPLVMAAYIPGYLNVIFTPEHKKEILRYTYNHQNEDGGWGLHIA-GPSMMF     176
MdOSC1              ENAGPNFYFPPLVMAAYIPGYLNVIFSAEHKKEILRYTYNHQNEDGGWGLHIA-GPSMMF     176
MdOSC3              ENAGPNFYFPPLVMAAYIPGYLNVIFSAEHKKEILRYTYNHQNEDGGWGLHIS-GPSMMF     176
XP_021810674.1      ------------------------------------------------------------     0
XP_021819927.1      QNTGPLFYTPPFVIALYITGHLNMVFSADHRKEMLWFMYCHQNEDGGWGLHIEEGQSMML     180

XP_021819911.1      CTALSYICMRILGEGPDGGQDNACARARKWILDHGSVTHIPSWGKTWLSILGVFEWSGSN     236
XP_021819916.1      CTALSYICMRILGEGPDGGQDNACARARKWILDHGSVTHMPSWGKTWLSILGVFEWSGSN     62
MdOSC5              CTALNYICMRILGEGPDGGQDKACARARKWILDHGSVTHIPSWGKTWLSILGVFEWSGSN     236
MdOSC2              CTALSYICMRILGEGPDGGQDNACARARKWILDHGSVTHIPSWGKTWLSILGVFEWSGSN     236
MdOSC4              CTALSYICMRILGEGPDGGQDNACARARKWILDHGSVTHIPSWGKTWLSILGVFEWSGSN     236
XP_021819928.1      TTCLNYCMMRILGDGPDGGRDNACARARKWILDRGGACYAASWGKTWMAILGVYDWEGSN     236
MdOSC1              TTCLNYCMMRILGDGPDGGRDNACARARKWILDRGGAYYSASWGKTWMAILGVYDWEGSN     236
MdOSC3              TTCLNYCMMRILGEGPDGGRDNACARARKWILDRGGAYYSASWGKTWMAILGVYDWEGSN     236
XP_021810674.1      ------------------------------------------------------------     0
XP_021819927.1      CTLLNYICMRLLGEGPDGGLDNACARARKWILDHGGATHSSSWGKIWMAMLGVYDWEGNN     240

XP_021819911.1      PMPPEFWVLPSFLPMHPAKMWCYCRMVYMPMSYLYGKRFVGPITPLILQLREELYAQPYN     296
XP_021819916.1      PMPPEFWMLPSFLPMHPAKMWCYCRMVYMPMSYLYGKRFVGPITPLILQLREELYAQPYD     122
MdOSC5              PMPPEFWILPSFLPMHPAKMFCYSRMVYMPMSYLYGKRFVGPITPLILQLREELYAQPYG     296
MdOSC2              PMPPEFWILPSFLPMHPAKMWCYCRMVYMPMSYLYGKRFVGPITPLILQLREELYAQPYD     296
MdOSC4              PMPPEFWILPSFLPMHPAKMWCYCRMVYMPMSYLYGKRFVGPITPLILQLREELYAQPYD     296
XP_021819928.1      PMPPEFWTYPTLLPFHPSKMFCYCRLTYLPMSYFYATRFVGPITPLVEELRQEIYCEPYS     296
MdOSC1              PMPPEFWTGSTLLPFHPSKMFCYCRLTYLPMSYFYATRFVGPITPLVEELRQEIYCESYN     296
MdOSC3              PMPPEFWTGSTLLPFHPSKMFCYCRLTYLPMSYFYATRFVGPITPLVEELRQEIYCEPYS     296
XP_021810674.1      ------------------------------------------------------------     0
XP_021819927.1      PIPPELWICPSFLPFHPAKILCYTRMSYLAMSYLFGKRVVGPITPLILQLREEIYNEPYN     300

XP_021819911.1      EINWKGVRHHCAKEDIYYPHPWIQDIMWDSLYICTEPLLNRWPFNKLIREKALQVTMKHI     356
XP_021819916.1      EINWKGVRHLCAKEDIYYPHPWIQDIMWDSLYICTEPLLTRWPFNKLVREKALQVTMKHI     182
MdOSC5              EINWKGVRRHCAKVDIYYPHPWIQDFLWDSLYVCTEPLLTRWPFNKLIRKRALEVTMEHV     356
MdOSC2              EINWKGVRHHCAKEDIYYPHPWIQDFLWDSLYICTEPLLTRWPFNKLIRKRALDVTMKHI     356
MdOSC4              EINWKGVRHHCAKEDIYYPHPWIQDFLWDSLYICTEPLLTRWPFNKMIRKRALEVTMKHI     356
XP_021819928.1      EINWSTVRHSCAKEDNYYPHGRVQRFMWDSLYTVVEPLLKRWPFKK-IRDNAIQFTIDQI     355
MdOSC1              EINWPKVRHWCATEDNYYPHGRVQRFMWDGFYNIVEPLLKRWPFKK-IRDNAIQFTIDQI     355
MdOSC3              EINWSKVRHWCAPDDNYYPHGRVQRFMWDSFYNIAEPLLKRWPFKK-IRDNAIQFTIDQI     355
XP_021810674.1      ------------------------------------------------------------     0
XP_021819927.1      EIKWSEVRHLCAKEDKYHPHRKLQCLMWDSLHTICEPLFTRWPFKK-LREKALQETSKHI     359
```

```
XP_021819911.1       HYEDENSRYITIGCVEKVLCMLACWAEDPNGDYFKKHLARIPDYLWVAEDGMKMQSFGSQ    416
XP_021819916.1       HYEDENSRYITIGCVEKVLCMLACWAEDPNGDYFKKHLARIPDYLWVAEDGMKMQSFGSQ    242
MdOSC5               HYEDENSRYITIGCVEKVLCMLACWVEDPNGDYFKKHLARIPDYLWVAEDGMKMQSFGSQ    416
MdOSC2               HYEDENSRYITIGCVEKVLCMLACWAEDPNGDYFKKHLARIPDYLWVAEDGLKMQSFGSQ    416
MdOSC4               HYEDENSRYITIGCVEKVLCMLACWAEDPNGDYFKKHLARIPDYLWVAEDGMKMQSFGSQ    416
XP_021819928.1       HYEDENSRYITIGCVEKPLMMLACWAEDPSGEAFKKHIPRVTDYIWLGEDGIKMQSFGSQ    415
MdOSC1               HYEDENSRYITIGCVEKPLMMLACWAEDPSGEAFKKHLPRVTDYIWLGEDGIKMQSFGSQ    415
MdOSC3               HYEDENSRYITIGCVEKPLMMLACWAEDPSGEAFKKHLPRVTDYIWLGEDGIKMQSFGSQ    415
XP_021810674.1       ------------------------------------------------------------    0
XP_021819927.1       HYEDENTRYITIGVGGKPLNMLACWAEDPNGESYKKHLARIADYIWVAEDGMTMQSFGSQ    419

XP_021819911.1       EWDTGFAIQALLASNLTDEIGPTLARGHDFIKKSQVKDNPSGDFKSMYRHISKGSWTFSD    476
XP_021819916.1       QWDTGFAIQALLASNLADEIGPTLARGHDFIKKSQVKDNPSGDFRSMHRHISKGSWTFSD    302
MdOSC5               QWDTGFAIQALLASNLTDEIAPTLARGHDFVKKSQVKDNPSGDFKSMYRHISKGSWTFSD    476
MdOSC2               LWDTGCAIQALLASNLTDEIAPTLARGHDFVKKSQVKDNPSGDFKSMHRHISKGSWTFSD    476
MdOSC4               QWDTGFAIQALLASNLTDEIAPTLARGHDFVKKSQVKDNPSGDFKSMHRHISKGSWTFSD    476
XP_021819928.1       SWDCALVIQALLAGNLNAEMGPVLKKAHEFLKISQVRINTSGDYLAHFRHISKGAWTFSD    475
MdOSC1               SWDCALVIQALLAGNLNAEMGPTLKKAHEFLKISQVRINTSGDYLSHFRHISKGAWTFSD    475
MdOSC3               SWDCALVIQALLAGNLNTEMAPTLKKAHEFLKISQVRVNTSGDYLAHFRHVSKGAWTFSD    475
XP_021810674.1       MWDASFAMQALLAANLNDELGSVLKKGHDFLKKSQVRDNPSGDFVAHFRHISKGGWTFSD    60
XP_021819927.1       MWDASFAIQALLAANLNDELGSVLKKGHDFIKKSQVRDNPSGDFLAYFRHISKGAWTFSD    479
                      ** . .:*****.**  *:. .* :.*:*:* ***: * ***: : .**:***.*****

XP_021819911.1       QDHGWQVSDCTAEGLKCCLLFSVMRPDIVGEKMEPERLYDSINVLLSLQSKNGGLAAWEP    536
XP_021819916.1       QDHGWQVSDCTAEGLKCCLLFSTMRPDIVGEKMEPQRLYDSVNVLLSLQSKNGGLAAWEP    362
MdOSC5               QDHGWQVSDCTAEGLKCCLLLSMMPPEMVGEKMEPERLFDAVNVLISLQSKNGGLAAWEP    536
MdOSC2               QDHGWQVSDSTADGLKCCLLLSMMPPEMVGEQMEPERLYDAVNVIISLQSKNGGLAAWEP    536
MdOSC4               QDHGWQVSDCTAEGLKCCLLLSMMPPELVGEKMEPERLYDAVNVLISLQSKNGGLAAWEP    536
XP_021819928.1       RDHGWQVSDCTAEALRCCCLFANMSPEVVGEPMEAECMYDAVNVIMSLQSPNGGVSAWEP    535
MdOSC1               RDHGWQVSDCTAEALRCCCIFANMSPEVVGEPMEAECMYDAVNVIMSLQSPNGGVSAWEP    535
MdOSC3               RDHGWQVSDCTAEALRCCCIFANMSPELVGEPMEAECMYDAVNVILTLQSPNGGVSAWEP    535
XP_021810674.1       QDHGWQVSDCTAEALKNCLLLSMLPPQLVGEQLEPERLYDAVNVVLSLQGPNGGVSAWEP    120
XP_021819927.1       QDQGLQVSDCTAEGLKCCLLLSMLPPQLVGEQLEPERLYDAVNVILSLQSPNGGVSAWEP    539
                     :*:* ****.**:.*: * ::: : *::*** :* : ::*::**:::**. ***::****

XP_021819911.1       AGAADWLEMLNPTEFFEDIVVEHEYVECTSSAIQALVLFKKLYPGHRKREIDHFITNATQ    596
XP_021819916.1       AGAAEWLEMLNPTEFFADIVVEHEYVECTSSAIQALVLFKKLYPGHRKKEIDHFITNATE    422
MdOSC5               AGAAEWLEMFNPSEFFADIVVEHEYVECTSSAIQALVLFKKLCPGHRKEEIDQFITAAAL    596
MdOSC2               AGAADWLEMLNPTEFFADIVVEHEYVECTSSAIQALVLFKKLYPGHRKKEIDQFITNAAQ    596
MdOSC4               AGAAEWLEMLNPTEFFADIVVEHEYVECTSSAIQALVLFKKLYPGHRKKEIDQFITNAAQ    596
XP_021819928.1       TGAPKWLEWLNPVEFLEDLVIEYEYIECTSSSIQALILFRKLYPGHRRKEINNFITRAAD    595
MdOSC1               TGAPKWLEWLNPVEFLEDLVIEYEYIECTSSSIQALTLFRKLYPGHRRKEINNFITRAAD    595
MdOSC3               TGAPKWLEWLNPVEFLEDLVIEYEYIECTSSSIQALTLFRKLYPGHRRKEINNFITRAAD    595
XP_021810674.1       AGAPKWLEWLNPIEFMGDLVIEYEHVECTSSSIQALALFRKLYPTHRRKQIDNFITTAAG    180
XP_021819927.1       AGAPKWLEWLNPVEFLDHLIIEYEHVECTSSSIQALALFRKLYPAHRKKQIDNFITTAAG    599
                     :** .*** :** **: .:::*:*::*****:**** **:** * **:.:*::*** *:

XP_021819911.1       YLESIQMPDGSWYGNWGVCFTYGSWFALGGLAAAGKTFNNCLAMRKGVNFLLKTQRENGG    656
XP_021819916.1       YLENIQMPDGSWYGNWGVCFTYGSWFALGGLAAAGKTFNNCLTVRKGVNFLLTTQRENGG    482
MdOSC5               YLENVQMADGSWYGNWGVCFTYGTWFALGGLTAAGKTFNNCAAMRKAISFLLAIQKENGG    656
MdOSC2               YLENTQMADGSWYGNWGVCFTYGTWFALGGLTAAGKTFNNCAVIRKAISFLLTIQKENGG    656
MdOSC4               YLENIQMEDGSWYGNWGVCFTYGTWFALGGLTAAGKTFNNCAVIRKAINFLLTIQKENGG    656
XP_021819928.1       YIEDIQYPDGSWYGNWGICFVYGTWFAIKGLEAAGRTYNNCEAVRKGVDFLLKTQREDGG    655
MdOSC1               YIEDIQYPDGSWYGNWGICFVYGTWFAIKGLEAAGRTYNNCEAVRKGVDFLLKTQRADGG    655
MdOSC3               YIEDIQYPDGSWYGNWGICFVYGTWFAIKGLEAAGRTYNNCEAVRKGVDFLLKTQRADGG    655
XP_021810674.1       FIEDIQSPDGSWYGNWGICFMYGTWFAIRGLEAAGKTYNNCEAIRRGVEFLLKTQRDDGG    240
XP_021819927.1       FIEDIQSPDGSWYGNWGICFIYGTWFAISGLEAAGKTYNNCEAIRRGVEFLLKTQRDDGG    659
                     ::*. *  *********:** **:***: ** ***:*:*** .:*:.:.***  *: :**

XP_021819911.1       WGESYLSCPKKEYVPLEGNRSNLVHTAWAMMGLIHAGQAHRDPAPLHRAVKLIINSQMEN    716
XP_021819916.1       WGESYLSCPKKEYVPLEENRSNLVHTAWAMMGLIQAGQAERDPAPLHRAAKLIINSQMEN    542
MdOSC5               WGESYLSCPKKEYVPLEGNRSNLVHTAWAMMGLIHAGQAERDPTPLHRAAKLIINSQMEN    716
MdOSC2               WGESYLSCPKKEYVPLEGNRSNLVHTAWAMMGLIHAGQAERDPTPLHRAAKLIINSQMEN    716
MdOSC4               WGESYLSCPKKEYVPLEGNRSNLVHTAWAMMGLIYAGQAERDPAPLHRAAKLIMNSQMEN    716
XP_021819928.1       WGEHYTSCTNKKYTAQ--DSTNLVQTALGLMGLIHGRQAERDPTPIHRAARALMNGQLDD    713
MdOSC1               WGEHYTSCTNKKYTAQ--DSTNLVQTALGLMGLIHGRQAERDPTPIHRAAAVLMNGQLDD    713
MdOSC3               WGEHYTSCTNKKYTAQ--DSTNLVQTALGLMGLIHGRQAERDPTPIHRAAAVLMKGQLDD    713
```

```
XP_021810674.1      WGESYISCTNKIYTPLEGDRSNVVQTAMGLMGLIHGGQAERDPTPIHQAAKMLINSQLEN     300
XP_021819927.1      WGESYISCETKIYTPLEGDRSNLVQTAMGLMGLIHGGQAERDPTPIHQAAKMLINSQLEN     719
                    *** * ** .* *.    : :*:*:** .:**** . **.***:*:*:*.  :::.*:::

XP_021819911.1      GDFPQQEITGVFMKNCTLHYAAYRNIYPLWALAEYRKWVPLPSKA-- 761
XP_021819916.1      GDFPQQEITGVFMKNCMLHYAAYRNIYPLWALAEYRKRVPLPSEA-- 587
MdOSC5              GDFPQQEITGAFMKNCMLHYANYRNIYPLWALAEYRRRVPFVDV--- 760
MdOSC2              GDFPQQEITGVIMKNCMLHYAAYRNIHPLWALAEYRKRVHCLPKPH- 762
MdOSC4              GDFSQQEITGVFNKNCMLHYAAYRNIYPLWALAEYRKWVPLPSKA-- 761
XP_021819928.1      GDFPQQELMGVFMRNAMLHYAAYRNIFPLWALGEYRTLVQLPTKRI- 759
MdOSC1              GDFPQQELMGVFMRNAMLHYAAYRNIFPLWALGEYRTLVSLPIKKIA 760
MdOSC3              GDFPQQELMGVFMRNAMLHYAAYRNIFPLWALGEYRTLVSLPIKKIA 760
XP_021810674.1      GDFPQQEVMGVFMRNVMLHYAAYRNTIPIWALAEYSNMVRVVV---- 343
XP_021819927.1      GDFPQQEVIGVFFRNAMLHYAAFRNIFPIWALAEYRNMVPMPLYV-- 764
                    *** ***: *.: :*  **** :**  *:***.**   *
```

Figure S1: Alignment of the sweet cherry and apple OSCs showing the conserved DCTAE (green highlight), M(W/Y)CY(C/S)R (cyan highlight), as well as the Q-X3-G-X-W motifs (yellow highlight) [11, 17-19].
