# Supplementary material for: Tuscan Varieties of Sweet Cherry Are Rich Sources of Ursolic and Oleanolic Acid: Protein Modeling Coupled to Targeted Gene Expression and Metabolite Analyses
Source: Molecules. 2019 Apr 22;24(8):1590. doi: 10.3390/molecules24081590 (PMC6515059; doi:10.3390/molecules24081590)
Supplement: Supplementary file 1 [file molecules-24-01590-s001.zip › molecules-471845-final-SM/molecules-471845-final-SM.pdf]

Supplementary Information

Sweet cherry OSCs (nucleotide and protein sequences)

>XM_021964236.1
TGATTTTACCTCTAATTTCTCCCTCTTCCTCTCTCTCTCTGCACACACACTCACAGTCACACAAACAG
TTGAGGATGGATAAAATATTCGAAAAAATGGGGCTTGGTGGTTTTTTATTTTATTGTCGGCGTCTGTAAG
CAAAACCATTGTATCTATATAAAGTGAGGTTCCCATGCATCAACCATCACAGACTCATTCAGTATTGTTT
GTGCTAAGCAACTAACTCAAGCAACCCGGCCTTCTTCCTTCCCTCATCACCAGCCAGTTTTTCCAGATCG
TTAATACGGGAAGAAACTTTTTGTACTATTTAATTACGTGATCAAGAGAAGATGTGGAAGATTAAGTTTG
GAGAGGGGGCAAATGACCCCTTGTTGTACAGCACAAACAACTTCCATGGAAGGCAGACATGGGAGTTTGA
CCCAGATGCAGGTACCCCTGAAGAGAGAGCAGAGGTTGAAGAAGCTCGTGAAAATTTCTACAGAAACCGC
TTCAAGGTTCAGCCCAGCAGTGATCTCCTCTGGCGTTTTCAGATGCAAAGAGAGAGAAACTTCAAACAAG
AAATTCCTCCAGTAAGAATTGGGGAGGGTGAGGAAATCACATATGATCAGGCCACAGCTGCATACAGGAG
GGCTGCTACCTTTTGGAATGCCTTGCAATCACCTCATGGACATTGGCCTGCTGAAAATGCTGGCCCAAAC
TTTTACTTTCCACCCCTGGTCATGGCTGCATACATTCCAGGATATCTTAATGTTATTTTCACACCTGAGC
ATAAGAAGGAAATCTTGCGTTATACATACAACCATCAGAATGAAGATGGTGGGTGGGGATTGCACATAGC
AGGGCCTAGTATGATGTTCACCACATGTCTTAACTACTGTATGATGCGTATACTCGGAGATGGCCCTGAT
GGCGGTCGTGACAACGCATGCGCAAGGGCACGAAAGTGGATTCTTGACCGTGGGGGTGCATGCTATGCTG
CATCATGGGGAAAAACCTGGATGGCGATCCTTGGTGTGTACGATTGGGAAGGCAGCAACCCCATGCCCCC
GGAGTTTTGGACTTACCCTACTCTGCTTCCTTTTCATCCATCAAAAATGTTCTGCTACTGCAGGTTGACT
TACCTGCCTATGTCTTACTTTTATGCCACAAGATTTGTTGGTCCAATCACTCCTCTTGTTGAAGAATTGA
GACAAGAAATTTACTGTGAACCGTACAGTGAAATTAACTGGAGTACAGTGCGCCATTCGTGTGCAAAGGA
AGATAACTACTATCCCCACGGCAGGGTTCAACGTTTTATGTGGGATAGTCTTTACACAGTTGTTGAGCCT
CTTCTTAAGCGCTGGCCCTTCAAGAAGATCAGAGACAATGCTATTCAATTTACAATTGACCAAATTCATT
ATGAAGATGAGAACAGTCGCTATATTACGATCGGATGCGTGGAGAAGCCATTGATGATGCTTGCTTGCTG
GGCCGAGGATCCTAGTGGAGAAGCTTTTAAGAAGCATATTCCTAGAGTTACAGATTATATATGGCTTGGA
GAAGATGGAATCAAGATGCAGAGTTTTGGCAGCCAGTCATGGGATTGTGCTCTTGTAATTCAAGCTTTGC
TTGCTGGGAATCTGAATGCTGAAATGGGACCTGTACTTAAGAAAGCACACGAATTCCTGAAGATATCTCA
GGTGAGGATCAATACTTCTGGCGACTACTTAGCTCATTTCCGTCACATTTCTAAGGGAGCATGGACTTTC
TCTGATCGGGATCACGGATGGCAAGTTTCAGATTGTACTGCAGAGGCATTGAGGTGTTGCTGCCTGTTTG
CAAATATGTCCCCAGAAGTTGTTGGTGAGCCAATGGAAGCTGAGTGTATGTATGATGCTGTCAATGTCAT
AATGTCTCTTCAGAGTCCAAATGGTGGTGTATCAGCCTGGGAGCCAACAGGAGCACCAAAATGGTTGGAG
TGGCTCAACCCTGTGGAATTTCTCGAGGACCTTGTCATCGAATACGAGTACATCGAGTGCACTTCATCTT
CAATCCAGGCCTTAATTTTGTTCAGGAAGTTGTACCCTGGGCACAGAAGGAAAGAGATCAACAATTTCAT
CACAAGGGCTGCAGACTACATTGAAGACATACAATATCCTGATGGCTCATGGTATGGAAACTGGGGAATC
TGTTTTGTGTATGGTACCTGGTTTGCAATCAAAGGGTTGGAGGCTGCAGGCAGAACATACAACAATTGCG
AGGCAGTACGCAAAGGTGTTGACTTTTTGCTCAAAACTCAAAGGGAAGATGGTGGTTGGGGAGAGCACTA
CACCTCATGCACAAACAAGAAATATACAGCTCAAGACAGTACAAATTTGGTCCAAACTGCACTTGGATTG
ATGGGTCTGATTCACGGCCGGCAGGCTGAGAGAGATCCAACTCCTATTCACCGTGCCGCAAGGGCATTGA
TGAACGGTCAATTGGACGACGGTGATTTCCCCCAACAGGAACTGATGGGAGTTTTTATGAGGAATGCCAT
GTTGCACTATGCAGCATATAGGAATATCTTCCCATTGTGGGCTCTCGGAGAATACCGTACACTGGTTCAG
TTGCCTACCAAAAGGATTTGAGACTTGAACCACAAGCAGCCACAAGCAGAGGAGTTCATGGACTTGCCAA
TACAACCAGAACTAATAAAAGCTTGTTCTAGTTGGGCCATATTTTTTCTTTTCTTATTTTATGTTTGTAG
AGTTTGATGTACTTGTAACCGCTGAAGAATGAATAATCTATGCATATATATAAATATATTGTATCTTACT
TATA

>XP_021819928.1
MWKIKFGEGANDPLLYSTNNFHGRQTWEFDPDAGTPEERAEVEEARENFYRNRFKVQPSSDLLWRFQMQR
ERNFKQEIPPVRIGEGEEITYDQATAAYRRAATFWNALQSPHGHWPAENAGPNFYFPPLVMAAYIPGYLN
VIFTPEHKKEILRYTYNHQNEDGGWGLHIAGPSMMFTTCLNYCMMRILGDGPDGGRDNACARARKWILDR
GGACYAASWGKTWMAILGVYDWEGSNPMPPEFWTYPTLLPFHPSKMFCYCRLTYLPMSYFYATRFVGPIT
PLVEELRQEIYCEPYSEINWSTVRHSCAKEDNYYPHGRVQRFMWDSLYTVVEPLLKRWPFKKIRDNAIQF
TIDQIHYEDENSRYITIGCVEKPLMMLACWAEDPSGEAFKKHIPRVTDYIWLGEDGIKMQSFGSQSWDCA
LVIQALLAGNLNAEMGPVLKKAHEFLKISQVRINTSGDYLAHFRHISKGAWTFSDRDHGWQVSDCTAEAL
RCCCLFANMSPEVVGEPMEAECMYDAVNVIMSLQSPNGGVSAWEPTGAPKWLEWLNPVEFLEDLVIEYEY
IECTSSSIQALILFRKLYPGHRRKEINNFITRAADYIEDIQYPDGSWYGNWGICFVYGTWFAIKGLEAAG
RTYNNCEAVRKGVDFLLKTQREDGGWGEHYTSCTNKKYTAQDSTNLVQTALGLMGLIHGRQAERDPTPIH
RAARALMNGQLDDGDFPQQELMGVFMRNAMLHYAAYRNIFPLWALGEYRTLVQLPTKRI

>XM_021954982.1

AGCCAGATGTGGGATGCTAGTTTCGCCATGCAAGCTTTGCTTGCTGCAAATCTGAATGATGAACTTGGAT
CTGTACTTAAGAAAGGACATGACTTCCTTAAGAAATCTCAGGTTAGGGATAATCCTTCTGGAGACTTTGT
AGCCCATTTTCGCCACATTTCTAAAGGAGGATGGACTTTCTCTGATCAAGACCATGGATGGCAAGTTTCT
GATTGTACTGCAGAAGCTTTGAAGAATTGCTTGTTGTTATCGATGCTGCCGCCACAACTCGTCGGTGAAC
AACTAGAACCTGAGCGTTTATATGATGCTGTCAATGTCGTACTTTCTCTGCAGGGTCCAAATGGTGGTGT
ATCAGCCTGGGAACCGGCAGGAGCTCCAAAATGGTTGGAGTGGCTCAACCCTATAGAATTTATGGGGGAT
CTCGTCATCGAATATGAGCACGTCGAGTGCACCTCATCTTCAATCCAGGCATTAGCTTTGTTTAGGAAGT
TATACCCCACCCACAGAAGGAAACAGATAGATAATTTCATCACAACTGCTGCAGGGTTCATTGAAGACAT
ACAAAGTCCTGATGGGTCATGGTATGGAAACTGGGGAATCTGCTTCATGTATGGAACATGGTTTGCAATC
AGAGGGTTAGAGGCTGCTGGCAAGACTTACAATAATTGTGAGGCTATACGCCGAGGTGTTGAATTTTTAC
TCAAAACACAGAGAGATGATGGTGGATGGGGAGAGAGCTACATTTCTTGTACAAACAAGATCTATACACC
TCTTGAAGGAGACCGATCCAATGTGGTGCAGACTGCAATGGGATTAATGGGTTTAATTCATGGTGGACAG
GCGGAGAGAGACCCTACTCCTATTCACCAAGCTGCAAAGATGTTGATCAATTCTCAATTGGAAAATGGCG
ATTTCCCCCAGCAGGAAGTGATGGGAGTTTTCATGAGGAATGTCATGTTACACTACGCAGCATACAGGAA
TACTATCCCAATTTGGGCTCTTGCAGAATACAGCAATATGGTTCGTGTAGTCGTGTAGATTAATCTACGT
GTATGTACGTATATACAAAGAGTGTTGTCTGCTTAACGCCATTAAGTAAAGGAAGAAAACAGTTTAAAAG
TTAATGAGTTATTTATGCAGCCTATTAAAGTACAAGGGGGTGAATATTCTCTTTATATTTGGTTTTGGAT
ATCAGCTTTACTTTTAAGCATGTATTTTAAATACTTTGTCAGAGTTGATAATAAATTTTATTAATAATAA
A

>XP_021810674.1
MWDASFAMQALLAANLNDELGSVLKKGHDFLKKSQVRDNPSGDFVAHFRHISKGGWTFSDQDHGWQVSDC
TAEALKNCLLLSMLPPQLVGEQLEPERLYDAVNVVLSLQGPNGGVSAWEPAGAPKWLEWLNPIEFMGDLV
IEYEHVECTSSSIQALALFRKLYPTHRRKQIDNFITTAAGFIEDIQSPDGSWYGNWGICFMYGTWFAIRG
LEAAGKTYNNCEAIRRGVEFLLKTQRDDGGWGESYISCTNKIYTPLEGDRSNVVQTAMGLMGLIHGGQAE
RDPTPIHQAAKMLINSQLENGDFPQQEVMGVFMRNVMLHYAAYRNTIPIWALAEYSNMVRVVV

>XM_021964235.1
AAACAATGCAAGCATTGCAAATGGTTTAAGAGCCCTTGAGAAATTTCCCAACCAAAAGCTTTTTGGTAGT
TCTTCATTAAGAAGTGTACAACAACATTAATTATAAGTAGTCTCCAGTGATCTCAGTCAAATTAATTGAG
ATCATCAAGAATTAATAATGTGGAAGCTGAAGTTTGGAGCTGAGGGTGAAAATGTTGACCCTTACTTGTT
CAGCACAAACAAGTTTGTGGGAAGGCAAATATGGGAGTTTGATCCTCATGCAGGCACACCTCAAGAGCGA
GCAGAGGTTGAAGAGGCTCGTCAAAACTTTTACAACAATCGTTACAAGGTCAAGGCCTCCAGTGACATCC
TCTGGCGAATGCAGCTTCACAGAGAGAGAAATTTCAAGCAAACAATTCCCCAAGTTAAAATCGAAGATGA
TGATGAGGAATGTAGACATGAAAAAGCCACCGTTGCATTCAAAAGGGCTGTTGCCTTCTGGTCTGCCTTG
CAATCACCACACGGCCATTGGCCCGCTCAAAATACTGGTCCTTTGTTTTATACTCCTCCCTTTGTCATAG
CCCTGTATATTACAGGCCATCTTAATATGGTTTTCTCTGCAGACCATCGTAAGGAAATGCTTTGGTTCAT
GTACTGTCACCAGAACGAAGATGGTGGGTGGGGATTACACATAGAAGAGGGCCAAAGTATGATGTTGTGT
ACACTTCTGAACTACATTTGTATGCGTCTACTTGGCGAAGGACCCGATGGTGGTCTAGACAATGCTTGTG
CAAGAGCAAGGAAATGGATTCTTGATCATGGTGGTGCAACACACTCTTCATCTTGGGGAAAGATTTGGAT
GGCAATGCTCGGTGTATATGATTGGGAGGGAAACAACCCTATTCCCCCAGAGCTTTGGATCTGCCCATCT
TTCCTCCCTTTTCATCCAGCAAAAATACTCTGCTATACTCGCATGAGTTACTTGGCTATGTCATATTTGT
TTGGGAAAAGAGTTGTTGGCCCCATCACACCCCTCATTCTGCAACTGAGAGAAGAAATCTACAATGAACC
TTACAATGAAATTAAATGGAGCGAAGTGCGCCATCTGTGTGCAAAAGAGGATAAATACCATCCCCATCGT
AAGTTACAATGCTTGATGTGGGATAGTCTTCACACAATCTGTGAGCCTCTTTTCACTCGCTGGCCCTTCA
AAAAGCTCAGAGAGAAGGCTCTTCAAGAAACCAGTAAACACATTCATTATGAAGACGAGAATACTCGATA
CATTACTATTGGAGTTGGGGGAAAGCCATTAAATATGCTTGCTTGCTGGGCTGAAGATCCGAATGGAGAA
TCTTACAAGAAGCATCTCGCAAGGATTGCAGATTACATATGGGTTGCAGAAGATGGAATGACAATGCAGA
GTTTTGGTAGCCAGATGTGGGATGCTAGTTTCGCCATTCAAGCTTTGCTTGCTGCAAATCTGAATGATGA
ACTTGGATCTGTACTTAAGAAAGGACATGACTTCATTAAGAAATCTCAGGTTAGGGATAATCCTTCTGGT
GACTTTTTAGCCTATTTTCGCCACATTTCTAAAGGAGCATGGACTTTCTCTGATCAAGACCAAGGATTGC
AAGTTTCTGATTGTACTGCAGAAGGTTTGAAGTGTTGCTTGTTGTTGTCCATGCTGCCACCACAACTCGT
TGGTGAACAACTAGAACCTGAGCGTTTATATGATGCTGTCAATGTCATACTTTCTCTGCAGAGTCCAAAT
GGTGGTGTATCAGCCTGGGAGCCGGCAGGAGCTCCAAAATGGTTGGAGTGGCTCAACCCTGTAGAATTTC
TCGACCATCTCATCATCGAATATGAGCATGTTGAGTGCACCTCATCTTCAATCCAAGCATTAGCTTTGTT
TAGGAAGTTATACCCTGCCCACAGAAAGAAACAGATAGATAATTTCATCACAACTGCTGCAGGGTTCATT
GAAGACATACAAAGTCCTGATGGGTCATGGTATGGAAACTGGGGAATCTGCTTCATTTATGGAACATGGT
TTGCAATCAGCGGGTTAGAGGCTGCTGGCAAGACTTACAATAATTGTGAGGCTATACGCCGAGGTGTTGA
ATTTTTACTCAAAACACAGAGAGATGATGGTGGATGGGGAGAGAGCTACATTTCTTGTGAAACCAAGATC
TATACACCTCTTGAAGGAGACCGATCCAATTTGGTGCAGACTGCAATGGGATTAATGGGTTTAATTCATG
GTGGACAGGCGGAGAGAGACCCTACTCCTATTCACCAAGCTGCAAAGATGTTGATCAATTCTCAATTGGA

AAATGGCGATTTCCCCCAGCAGGAAGTGATCGGAGTTTTCTTTAGGAATGCCATGTTACACTACGCAGCA
TTCAGGAATATTTTCCCAATTTGGGCTCTTGCAGAATACCGCAATATGGTTCCAATGCCTCTCTATGTGT
AGTCGTGTAGATTAATCTACGTGTATGTACGCATATACGAAGAGTGTTGTCTGCTTAACGCCATTAGGTA
AAGGAAGAAAACAGTTTAAAAGTTAATGAGTTATTTATGCAGATTATTAAAATACAAGGGGGTGACTATT
CTCTTTATATTTGGTTTTGGATATCAGCTTTACTTTTAAGCATGTATTTTAAATATACTTTGTCAGAGTT
GATGATAAATTTTATTAATAATAAATA

>XP_021819927.1
MWKLKFGAEGENVDPYLFSTNKFVGRQIWEFDPHAGTPQERAEVEEARQNFYNNRYKVKASSDILWRMQL
HRERNFKQTIPQVKIEDDDEECRHEKATVAFKRAVAFWSALQSPHGHWPAQNTGPLFYTPPFVIALYITG
HLNMVFSADHRKEMLWFMYCHQNEDGGWGLHIEEGQSMMLCTLLNYICMRLLGEGPDGGLDNACARARKW
ILDHGGATHSSSWGKIWMAMLGVYDWEGNNPIPPELWICPSFLPFHPAKILCYTRMSYLAMSYLFGKRVV
GPITPLILQLREEIYNEPYNEIKWSEVRHLCAKEDKYHPHRKLQCLMWDSLHTICEPLFTRWPFKKLREK
ALQETSKHIHYEDENTRYITIGVGGKPLNMLACWAEDPNGESYKKHLARIADYIWVAEDGMTMQSFGSQM
WDASFAIQALLAANLNDELGSVLKKGHDFIKKSQVRDNPSGDFLAYFRHISKGAWTFSDQDQGLQVSDCT
AEGLKCCLLLSMLPPQLVGEQLEPERLYDAVNVILSLQSPNGGVSAWEPAGAPKWLEWLNPVEFLDHLII
EYEHVECTSSSIQALALFRKLYPAHRKKQIDNFITTAAGFIEDIQSPDGSWYGNWGICFIYGTWFAISGL
EAAGKTYNNCEAIRRGVEFLLKTQRDDGGWGESYISCETKIYTPLEGDRSNLVQTAMGLMGLIHGGQAER
DPTPIHQAAKMLINSQLENGDFPQQEVIGVFFRNAMLHYAAFRNIFPIWALAEYRNMVPMPLYV

>XM_021964219.1
CCAATCTCTACAATAAGGTTTGTGCAGCTTCTTCTTTGCAAACTGCAAACTGCACGTACAGGGTTCTTAT
TGAGAAGATACATATAAAGGTAGATCAGAAGGGGTAGCTTGATATAATAAGAAAGAGATATTGAAGAATG
TGGAAGCTTAAGGTTGCAGATGGAGGGAATGACCCTTACATCTACAGCACAAACGACTTTGTGGGGAGGC
AGATATTTGAGTTTGATCCTGAGGCAGGAACTCCTGAAGAGCGAGCCGAGGTGGAAGAAGCTCGTCTTAA
TTTTTACAATAATCGCTATAAGGTTAAGCCTAGTGGTGACCTCATATGGCGTATGCAGTTCCTACGAGAG
AAGAACTTCAAACAGACAATTCCCGAAGTAAAGGTTGAGGATGGCGAAGAAATCACATATGAAAAGGCCA
CAGCTTCATTGAGAAGGTCTGTCCACTTCTTTTCAGCTTTGCAAGCAAGTGATGGCCATTGGCCTGCTGA
AAATGCTGGCCCATTGTTTTTCCTTCCTCCTCTGGTCATGTGTGCATACATTACAGGGCATCTTAATACT
GTATTCCCAGCAGAGCATCGAAAAGAAATTATGCGTTACATATACTATCATCAGAATGAAGATGGTGGTT
GGGGATTACATATTGAAGGCCACAGCACCATGTTCTGCACAGCTCTGAGCTACATTTGTATGCGCATTCT
TGGAGAAGGACCTGATGGAGGCCAAGACAATGCTTGTGCAAGAGCGAGGAAATGGATTCTTGACCATGGT
AGTGTTACACACATTCCCTCTTGGGGAAAGACTTGGCTTTCGATACTTGGTGTTTTTGAGTGGTCTGGTA
GCAACCCAATGCCCCCAGAGTTTTGGGTTCTGCCTTCATTTCTTCCTATGCATCCAGCAAAAATGTGGTG
CTATTGTAGGATGGTTTACATGCCTATGTCATATTTATATGGGAAAAGGTTTGTTGGTCCAATCACACCT
CTCATTCTGCAGTTAAGGGAAGAACTTTATGCTCAACCTTACAATGAAATTAATTGGAAGGGAGTTCGCC
ATCACTGCGCGAAAGAGGATATCTACTACCCTCACCCTTGGATACAGGACATCATGTGGGATAGTCTCTA
CATATGTACAGAGCCTCTTCTTAATCGCTGGCCGTTTAACAAGTTGATCAGAGAAAAAGCTCTTCAAGTA
ACAATGAAGCATATTCATTATGAAGATGAAAACAGCAGATATATTACCATTGGGTGTGTGGAAAAGGTGT
TATGTATGCTTGCTTGTTGGGCTGAAGATCCAAATGGAGACTATTTTAAGAAGCATCTTGCTAGGATCCC
AGATTATTTATGGGTTGCTGAAGATGGGATGAAGATGCAGAGTTTTGGTAGTCAAGAGTGGGATACTGGG
TTTGCAATTCAAGCTTTGCTTGCTAGCAATCTCACTGATGAAATTGGACCTACGCTCGCTAGAGGACATG
ATTTCATCAAGAAATCTCAGGTCAAGGACAATCCATCTGGTGACTTCAAAAGCATGTACCGCCATATTTC
CAAGGGATCATGGACTTTCTCTGATCAAGACCATGGATGGCAAGTTTCTGATTGCACTGCAGAGGGTTTA
AAGTGTTGCCTATTGTTCTCGGTGATGCGACCGGATATAGTTGGTGAAAAAATGGAACCTGAGCGATTAT
ATGATTCTATCAATGTCCTACTTTCCCTACAGAGTAAAAATGGTGGTTTGGCAGCCTGGGAACCAGCAGG
AGCTGCAGATTGGTTAGAAATGTTAAATCCCACTGAATTCTTCGAGGACATTGTGGTTGAGCATGAATAT
GTTGAATGCACTTCATCTGCAATTCAGGCTTTGGTTCTGTTTAAGAAGTTGTACCCTGGGCACAGAAAGA
GAGAGATTGATCATTTCATTACCAATGCTACGCAGTACCTTGAAAGTATACAAATGCCAGATGGTTCATG
GTATGGAAATTGGGGAGTTTGCTTCACGTATGGTTCCTGGTTTGCCCTTGGAGGGTTAGCAGCTGCCGGC
AAGACTTTCAATAATTGTTTAGCCATGCGCAAGGGGGTTAACTTTCTACTCAAAACACAGAGAGAGAATG
GTGGTTGGGGAGAGAGCTACCTTTCATGTCCCAAAAAGGAGTACGTTCCGCTTGAAGGAAACCGATCAAA
TTTAGTACACACTGCATGGGCTATGATGGGTCTGATTCATGCTGGACAGGCACACAGAGACCCTGCACCT
CTTCATCGCGCGGTGAAGTTGATTATCAATTCTCAAATGGAAAATGGGGATTTTCCCCAACAGGAAATCA
CAGGAGTGTTCATGAAGAACTGCACGCTACATTATGCAGCTTACAGAAATATCTACCCTCTGTGGGCTCT
TGCAGAGTACCGCAAGTGGGTCCCATTGCCTTCCAAAGCCTAAATACTGTATTGTGCAGCTCATCCAACT
TGAAGGATGCAACATATATATAAGAGCCAATTGTGGAAATTCATTTTTATGAGGCTCAGCTTGA

>XP_021819911.1
MWKLKVADGGNDPYIYSTNDFVGRQIFEFDPEAGTPEERAEVEEARLNFYNNRYKVKPSGDLIWRMQFLR
EKNFKQTIPEVKVEDGEEITYEKATASLRRSVHFFSALQASDGHWPAENAGPLFFLPPLVMCAYITGHLN

TVFPAEHRKEIMRYIYYHQNEDGGWGLHIEGHSTMFCTALSYICMRILGEGPDGGQDNACARARKWILDH
GSVTHIPSWGKTWLSILGVFEWSGSNPMPPEFWVLPSFLPMHPAKMWCYCRMVYMPMSYLYGKRFVGPIT
PLILQLREELYAQPYNEINWKGVRHHCAKEDIYYPHPWIQDIMWDSLYICTEPLLNRWPFNKLIREKALQ
VTMKHIHYEDENSRYITIGCVEKVLCMLACWAEDPNGDYFKKHLARIPDYLWVAEDGMKMQSFGSQEWDT
GFAIQALLASNLTDEIGPTLARGHDFIKKSQVKDNPSGDFKSMYRHISKGSWTFSDQDHGWQVSDCTAEG
LKCCLLFSVMRPDIVGEKMEPERLYDSINVLLSLQSKNGGLAAWEPAGAADWLEMLNPTEFFEDIVVEHE
YVECTSSAIQALVLFKKLYPGHRKREIDHFITNATQYLESIQMPDGSWYGNWGVCFTYGSWFALGGLAAA
GKTFNNCLAMRKGVNFLLKTQRENGGWGESYLSCPKKEYVPLEGNRSNLVHTAWAMMGLIHAGQAHRDPA
PLHRAVKLIINSQMENGDFPQQEITGVFMKNCTLHYAAYRNIYPLWALAEYRKWVPLPSKA

>XM_021964224.1
TATTTTTAGAATCAAGATGGTGGTTGGGGATTACATATTGAAGGCCACAGCACCATGTTCTGCACAGCCC
TGAGCTACATTTGTATGCGCATTCTCGGAGAAGGACCTGATGGGGGCCAGGACAATGCTTGTGCCAGAGC
AAGGAAGTGGATTCTTGATCATGGTAGTGTCACACACATGCCCTCTTGGGGAAAGACTTGGCTTTCGATA
CTTGGCGTGTTTGAGTGGTCTGGAAGCAACCCAATGCCGCCGGAGTTTTGGATGCTGCCTTCATTTCTTC
CTATGCATCCAGCAAAAATGTGGTGCTATTGTAGGATGGTTTACATGCCTATGTCATATTTATATGGGAA
GAGGTTTGTTGGCCCAATCACACCTCTCATTCTGCAATTAAGGGAAGAACTATATGCTCAACCTTACGAT
GAAATTAATTGGAAGGGAGTGCGCCATCTTTGCGCGAAAGAGGATATCTACTACCCACACCCTTGGATAC
AGGACATCATGTGGGATAGTCTCTACATATGTACAGAGCCTCTTCTTACTCGCTGGCCGTTTAACAAGTT
GGTCAGAGAAAAAGCTCTTCAAGTAACAATGAAGCATATTCATTATGAAGATGAAAACAGCAGATATATT
ACCATTGGATGTGTGGAAAAGGTGTTATGTATGCTTGCTTGTTGGGCTGAAGATCCAAATGGAGACTATT
TTAAGAAGCATCTTGCTAGGATCCCAGATTATTTATGGGTTGCTGAAGATGGGATGAAGATGCAGAGTTT
TGGAAGTCAGCAGTGGGATACTGGTTTTGCCATTCAAGCTTTGCTTGCTAGCAATCTCGCTGATGAAATT
GGACCTACGCTCGCTAGAGGACATGACTTCATCAAGAAATCTCAGGTCAAGGACAATCCATCTGGTGACT
TCAGAAGCATGCACCGCCACATTTCTAAAGGATCATGGACTTTCTCAGATCAAGACCATGGATGGCAAGT
TTCTGATTGCACCGCAGAGGGTTTAAAGTGTTGCCTGCTGTTCTCGACGATGCGACCAGATATAGTTGGT
GAAAAAATGGAACCTCAGAGGTTATACGATTCTGTCAATGTCCTACTTTCCCTACAGAGTAAGAATGGTG
GTTTAGCAGCCTGGGAACCAGCAGGAGCAGCAGAGTGGTTAGAAATGTTAAATCCCACAGAATTCTTCGC
GGACATTGTGGTTGAGCACGAATATGTTGAATGCACTTCATCTGCAATTCAGGCTTTAGTTCTGTTTAAG
AAGTTGTATCCCGGGCACAGGAAAAAAGAGATTGATCATTTCATTACCAATGCTACAGAGTACCTTGAAA
ACATACAAATGCCAGATGGTTCATGGTATGGAAATTGGGGAGTTTGCTTTACATATGGTAGCTGGTTTGC
CCTTGGAGGGTTAGCGGCTGCCGGAAAGACTTTCAATAATTGTTTAACCGTGCGCAAAGGCGTTAACTTT
CTACTCACGACACAAAGAGAGAACGGTGGCTGGGGAGAGAGCTATCTTTCATGTCCAAAAAAGGAGTACG
TTCCTCTCGAAGAAAACCGATCGAATTTAGTACACACTGCATGGGCTATGATGGGTCTGATTCAAGCTGG
ACAGGCAGAAAGAGATCCTGCACCTCTTCATCGTGCAGCAAAGTTGATTATCAATTCTCAAATGGAAAAT
GGAGATTTTCCCCAACAGGAAATCACAGGAGTGTTCATGAAGAACTGCATGCTACATTATGCAGCTTATA
GAAATATCTACCCTCTGTGGGCTCTTGCAGAGTACCGCAAGCGGGTTCCATTGCCTTCGGAAGCCTAAAT
CGCTAACCGTATTGTGTAGCTCATCCAACATAAAGGATTAAACATATATATAAGAGCCAATTGTTGAAAA
TCAATTTTATGAGGGTTCAGCTTGATGCTTAGTGAGAATATATGTCTTTTTCATGTCTTCATTCCTCTCA
TCTTCCGGATATTTTCCTTTCTCATTCACACAATCACGGTGGCATGAGGGTTCGAATTTTAAACTACTGG
TCTGCAAGTTAATGCTATTTTTCACTGAA

>XP_021819916.1
MFCTALSYICMRILGEGPDGGQDNACARARKWILDHGSVTHMPSWGKTWLSILGVFEWSGSNPMPPEFWM
LPSFLPMHPAKMWCYCRMVYMPMSYLYGKRFVGPITPLILQLREELYAQPYDEINWKGVRHLCAKEDIYY
PHPWIQDIMWDSLYICTEPLLTRWPFNKLVREKALQVTMKHIHYEDENSRYITIGCVEKVLCMLACWAED
PNGDYFKKHLARIPDYLWVAEDGMKMQSFGSQQWDTGFAIQALLASNLADEIGPTLARGHDFIKKSQVKD
NPSGDFRSMHRHISKGSWTFSDQDHGWQVSDCTAEGLKCCLLFSTMRPDIVGEKMEPQRLYDSVNVLLSL
QSKNGGLAAWEPAGAAEWLEMLNPTEFFADIVVEHEYVECTSSAIQALVLFKKLYPGHRKKEIDHFITNA
TEYLENIQMPDGSWYGNWGVCFTYGSWFALGGLAAAGKTFNNCLTVRKGVNFLLTTQRENGGWGESYLSC
PKKEYVPLEENRSNLVHTAWAMMGLIQAGQAERDPAPLHRAAKLIINSQMENGDFPQQEITGVFMKNCML
HYAAYRNIYPLWALAEYRKRVPLPSEA

Sweet cherry CYPs(nucleotide and protein sequences)

>XM_021961069.1
ATAAAAACGACTCATATCAGATCCACTTCCTTCAAACCATTCAAACAGACTCAAAGGCACGTTCCTCACT
CGTCCCACACAAAACACAAAGCTCCTCTCTCATTCTTCATCAAACACTGATCACAACAGTTTGATTAATC
ATGGAGCATTTCTATCTCACCCTCCTCTTGGGTTTTGTCTCCTTCATCTCTTTTTCTCTCTTTGTGCTCT
TCTACCGCCACAGAGCTCAGTTCACCGGAAACAACCTCCCGCCTGGCAAAGTTGGCTACCCTGTGATTGG
TGAGAGCTATGAGTTCTTGGCCTCAGGATGGAAAGGCCACCCTGAGAAGTTCATCTTTGACCGCATGACC
AAGTACTCATCTGACGTCTTCAAGACCTCCATCTTCGGCGAGAAGGCTGCCATCTTCTGCGGCGCTGCCT
GCAACAAGTTCTTGTTCTCCAATGAAAACAAGCTTGTCACCGCATGGTGGCCTAGCTCTGTCAACAAGGT
CTTCCCTTCTTCCCAAGAGACTTCCGCCAAAGAGGAGGCCAAGAAGATGAGAAAGATGCTCCCCAACTTC
ATGAAGCCTGAGGCTCTCCAACGATACATCGGGATCATGGACACTGTTGCCCAGAGGCACTTTGCTGATG
GTTGGGAAAACAGGAAGGAAGTTGAAGTCTTCCCCCTTGCCAAGAACTACACCTTTTGGCTTGCTGCACG
GTTGTTTGTTAGCCTTGAGGACGCAACGGAAATAGCAAGGCTAGGCGATCCATTCGCCCTGTTGGCCTCC
GGGATCATATCGATGCCTCTGGACTTCCCCGGAACTCCGTTCTACAAAGCCATCAAGGCGTCCAACTTCA
TCAGAGAGGAGCTGACAAAGATCATCAAGCAGAGGAAGGTTGATCTGGCTGAGGGCAAGGCGTCCCCAAC
ACAAGACATATTGTCACATATGTTGTTGTTGTGCGATGAGCATGGAAGTCACATGAAGGAACACGATATT
GCCGATAAGATTTTGGGGTTGTTGATTGGTGGGCATGACACGGCCAGCGCTACCTGCACTTTCATTGTCA
AGTATCTTGCTGAGCTTCCTCACATTTACGATGAGGTCTACAAGGAGCAAATGGAGATCCTGAGTTGCAA
AGCCCCAGGGGAGTTGTTGAACTGGGATGACCTACAGAAGATGAAATACTCATGGAACGTAGCCCAAGAA
GTGCTGAGATTGGCACCACCTCTTCAAGGAGCTTTCAGGGAAGCCTTGGCTGACTTTGTCTTCAATGGTT
TCACCATTCCAAAGGGCTGGAAGTTATATTGGAGCGCAAACTCAACACACAAGAACGCAGATTACTTCCC
GGAGCCATTTAAATTCGACCCAACAAGATTCGAAGGAAATGGGCCAGCACCCTACACCTTTGTGCCCTTT
GGAGGAGGCCCCAGGATGTGCCCAGGCAAAGAGTATGCCCGCCTGGAAATTCTAGTGTTCATGCACAACT
TGGTCAAGAGGTTCAAGTGGGAGAAGATTCTTCCCGATGAGAAGATCGTCGTTGACCCTCTCCCCATGCC
CGCCAAGGGCCTCCCCGTCCGCCTTTTTCCTCACAAAAAGTCTGCTTAACTTCACTTGAAAGGGCTGGCT
GGCTACGTGCATTTCCTTCCTTCATTGTCTATTTAATTATCTAGCTAAGTTGCAGATGTATACATAAAAC
AATCGAACAAGGCTTTGTTTCTTCTTTTTTTGGGATCCTTTTTCTTTTTCTTGGTTATTGTTGTATTTGT
TCTTCATGTCCACTGTACGTGTTTAATCAGGGCTTAAGCTCGTACGTGCCACTTGATGTT

>XP_021816761.1
MEHFYLTLLLGFVSFISFSLFVLFYRHRAQFTGNNLPPGKVGYPVIGESYEFLASGWKGHPEKFIFDRMT
KYSSDVFKTSIFGEKAAIFCGAACNKFLFSNENKLVTAWWPSSVNKVFPSSQETSAKEEAKKMRKMLPNF
MKPEALQRYIGIMDTVAQRHFADGWENRKEVEVFPLAKNYTFWLAARLFVSLEDATEIARLGDPFALLAS
GIISMPLDFPGTPFYKAIKASNFIREELTKIIKQRKVDLAEGKASPTQDILSHMLLLCDEHGSHMKEHDI
ADKILGLLIGGHDTASATCTFIVKYLAELPHIYDEVYKEQMEILSCKAPGELLNWDDLQKMKYSWNVAQE
VLRLAPPLQGAFREALADFVFNGFTIPKGWKLYWSANSTHKNADYFPEPFKFDPTRFEGNGPAPYTFVPF
GGGPRMCPGKEYARLEILVFMHNLVKRFKWEKILPDEKIVVDPLPMPAKGLPVRLFPHKKSA

>XM_021967982.1
ATGGAAACCCTCTACCTTATCCTATCTTTAGGTGCTGCTTTTTTAGCTCTAACCATCTTTGCATTCAAAG
GGAAATCAGATGATGGCAAAAACCTTCCACCGGGGAGCTTGGGGTGGCCTATTGTGGGTGAAACTCTGGA
GTTTCTGTTTGGGAAGCCAGAAATCTTTGTCTCCAAGAGGATGAGGAGGTACTCCCCTGAAATCTTCAAG
ACCAAGATTCTTGGAGAGAAAACTGCCGTTATTTGTGGTCCTAATGGACACAAATTTCTGTTTTCTAATG
AGCAGAAGTACTTCACAGCTTTTCGACCACATTCGATGCAAAAGATGTTTCGTTCGTATAAGGCTCCAGC
AGCAGCTGCTGCTGCTGCTGCTCCTCCAGCAGTCGCAGAACCTGCTCGTGACGAAGAAGCCAAAGTGTTG
AGGTCACCGGGCTTTTTGAAGCCAGAAGCATTGGTAAGGTACTTGGGGGTAATGGACTCTATCACCCAAG
CACAGATGAAGGCCTATTGGGAAGGCAAAGATGAGGTGGAGGTGTACCCTCTGACCAAGACCCTCACTCT
AGGCCTTGCATGCAGATTCTTCTTGGGCATAGATGAGCCAGACAGAATTGCAAGGTTTGTGAGCAATTTC
GATGATGTGACTGTTGGGATGCATTCACTTATTCTGAATTTCCCAGGAACAACATTCTACAAAGCAACCA
AAGCAGCCGATGAACTTCGAAGGGAGTTGAAGATTGTGATTCAGGAGAAGAAGACTGCAATGGCATCAGG
AGCTCCCATGCATGACATATTGTCACATATGATTTTGGCAAGTGACCCAACTGGCAAACACATGCCTGAG
GCTGAGATTGCTGATAAGATCATGGGTTTGCTCACAGCAGGATACAGCACTGTGGCTACTGCCATGACTT
TCTTCATGAAATATGTTGGAGAGAGGCCAGACATTTATGCCAAAGTCCTAGCAGAACACAAGGAGATTGC
AGAATCAAAGAAGCCTGGACAATTTTTGGAGTGGGATGACATCAACAAGATGAAGTACTCATGGAATGCA
GTGAATGAAGTGATGAGATTCACACCACCACTTCAGGGGACATTCAGAGAGGCCTTGACTGATTTCACCT
ACGCTGGTTACACCATCCCAAAGGGCTGGAAGGTATATTGGACTGTTAGTACAACAAACATGAACCCACA
GTACTTTCCCAACCCAGAAAAGTTTGACCCCTCAAGATATGATGACTTGAATGCATTCCCAGCTTTCACA
TTTGTCCCATTTGGAGGAGGACCAAGAATGTGCCCTGGCAAAGAGTATGCAAGGCTAGCCATACTCACTT
TTGTTCACAATGTGGTGATGAGGTTCAAATGGGAAGTGCTATTTCCTAACGAAAAGATCACAGGTGACAT
GATGCCGACACCGGAGAAAGGACTTCCAGTTCGTCTTCATCGTCACTAG

>XP_021823674.1
METLYLILSLGAAFLALTIFAFKGKSDDGKNLPPGSLGWPIVGETLEFLFGKPEIFVSKRMRRYSPEIFK
TKILGEKTAVICGPNGHKFLFSNEQKYFTAFRPHSMQKMFRSYKAPAAAAAAAAPPAVAEPARDEEAKVL
RSPGFLKPEALVRYLGVMDSITQAQMKAYWEGKDEVEVYPLTKTLTLGLACRFFLGIDEPDRIARFVSNF
DDVTVGMHSLILNFPGTTFYKATKAADELRRELKIVIQEKKTAMASGAPMHDILSHMILASDPTGKHMPE
AEIADKIMGLLTAGYSTVATAMTFFMKYVGERPDIYAKVLAEHKEIAESKKPGQFLEWDDINKMKYSWNA
VNEVMRFTPPLQGTFREALTDFTYAGYTIPKGWKVYWTVSTTNMNPQYFPNPEKFDPSRYDDLNAFPAFT
FVPFGGGPRMCPGKEYARLAILTFVHNVVMRFKWEVLFPNEKITGDMMPTPEKGLPVRLHRH

>XM_021959971.1
ATGAATACACTTCTTGTCACCATTTTCCTCTTAGTCATCCCCATCTTCTTTCTCATTACGAGAAGAAGAA
AATCATCGAAAAGGCTTCCTCCAGGTTCATTGGGACTACCCATAATAGGACAGAGCCTTGGCTTTCTTCG
AGCCCTGCGCAACAACACTGCAGAAAAATGGCTTGAACAAAGAATAACCAAGTATGGTCCGGTTTCAAAG
CTGAATCTCTTTGGCAAGCCAACAGTATTCATTCATGGACAGGCTGCAAACAAGTTTGTATTCAACAATG
ATGGCACAGCCATTACCAGCCAACAACCTGAGTCTACTCGAATGATTTTAGGTGACCGGAACATATTGGA
GCTGAGTGGCGAGGATCACAAGCGAGTTAGAAATGCTCTTATGGTGTTCTTGAAGCCTGAGTCACTGAAG
CAATATGTGGGAAAAATGGACGAAGAAATCAGGAAGCATCTTGAGTTGAATTGGCATGGGAAGCAAAAAA
TAACAGTCTTGCCTCTCATGAAGAACCTCACGTTCAACATTATATGCTCACTGCTTTTTGGGGTTGAACG
AGGACCTCGCAGAGACGAACTCATTGAGTGTTTTCAACTAATGATAGAAGGAGTGTGGTCAGTGCCTCTT
AACTTGCCGTTCACGCGTTTCAACTGCAGCATCAAGGCAAGCAAGAGGGTTCAAAACATGCTTAAGGAAC
TAATTTGTGAAAAGAGGATGCAACTTGAGCAAAAAACTGCTTTACCACTCCAAGACCTTATCACTTGCTT
GCTTAGCATTCGAAATGTTGACAATGAAGAAGAATTAACTGAAAAAGAGATCTTGCACAATATCATAATA
GTTATGGTTGCAGGATATGACACTTCATCTGTGGTCATTACTTTTCTTTTGCGGCTTCTAGCTAATGAAC
CTGCTGTTTATGCAGCCCTTCTTCAAGAACAGGAAGAAATAGCTAGAAGCAAATCCTTTGGGGAACTTCT
GACATGGGAAGACCTTGCTAAGATGAAGTACACATGGAGGGTAACAATGGAAATTCTGAGGACAACTCCT
CCTGTCTTTGGTGGCATGAGGAGGGCTATGAAAGATATTGAGTATGGAGGATTCCTCATCCCTCAAGGGT
GGCAAATATTCTGGGCTATTCCTATGACACACAATGACGATAGCATATTCCCTGAGCCATCAAAGTTCGA
TCCAAGTAGGTTCGAGAACCAAAAATCAGTTCCACCCTACAGCTTTGTTCCATTCGGAGGAGGAACACGA
ATATGTCCAGGATATGAGTTTGCCAGGATTGAAATCCTTGTTGCAATCCATTATATGGTAACTCAATTCA
CATGGAAGCTATGCGCAGACAACAAGTTCAGTAGGGTTCCCATGCCAGTACCTACTCAAGGACTACCTAT
AGAAATCATGCCAAGGAAACAAATGTAA

>XP_021815663.1
MNTLLVTIFLLVIPIFFLITRRRKSSKRLPPGSLGLPIIGQSLGFLRALRNNTAEKWLEQRITKYGPVSK
LNLFGKPTVFIHGQAANKFVFNNDGTAITSQQPESTRMILGDRNILELSGEDHKRVRNALMVFLKPESLK
QYVGKMDEEIRKHLELNWHGKQKITVLPLMKNLTFNIICSLLFGVERGPRRDELIECFQLMIEGVWSVPL
NLPFTRFNCSIKASKRVQNMLKELICEKRMQLEQKTALPLQDLITCLLSIRNVDNEEELTEKEILHNIII
VMVAGYDTSSVVITFLLRLLANEPAVYAALLQEQEEIARSKSFGELLTWEDLAKMKYTWRVTMEILRTTP
PVFGGMRRAMKDIEYGGFLIPQGWQIFWAIPMTHNDDSIFPEPSKFDPSRFENQKSVPPYSFVPFGGGTR
ICPGYEFARIEILVAIHYMVTQFTWKLCADNKFSRVPMPVPTQGLPIEIMPRKQM

OSC protein sequences used for phylogenetic analysis

>BPY AB055512 BETA-AMYRIN SYNTHASE [BETULA PLATYPHYLLA]BAB83088
QASDGHWPAENA
GPLFFLPPLVMCMYITGHLNTVFPAEHQKEILRYIYYHQNEDGGWGLHIEGHSTMFCTAL
SYICMRILGEGPDGGQDNACARARKWILDHGGVTHMPSWGKTWLSILGIFEWIGSNPMPP
EFWILPSFLPMHPAKMWCYCRMVYMPMSYLYGKRFVGPITPLILQLREELYTQPYHQVNW
KKVRHLCAKEDIYYPHPLIQDLLWDSLYIFTEPLLTRWPFNKLVREKALQVTMKHIHYED
ENSRYITIGCVEKVLCMLACWVEDPNGDYFKKHIARIPDYIWVAEDGIKMQSFGSQEWDT
GFAIQALLASNLTDEIGPTLARGHDFIKKSQVKDNPSGDFESMHRHISKGSWTFSDQDHG
WQVSDCTAEGLKCCLLFSIMPPEIVGEKMEPEQLYDSVNVLLSLQSKNGGLAAWEPAGAQ
EWLELLNSTEFFADIVIEHEYIECTASAMQTLVLFKKLYPGHRKKEIENFIKNAAQFLQV
IQMPDGSWYGNWGVCFTYGTWFALGGLAAVGKTYNNCLAVRRAVDFLLRAQRDNGGWGES
YLSCPKKEYVPLEGNKSNLVHTAWAMMGLIHAGQAERDPTPLHRAAKLIINSQLEDGDF

>ETAS AB206469 BETA-AMYRIN SYNTHASE [EUPHORBIA TIRUCALLI]BAE43642
QASDGHWPAENA
GPLFFLPPLVMCLYITGHLDTVFPAPHRLEILRYIYCHQNEDGGWGLHIEGHSTMFCTVL
SYICMRLLGEGPNGGQDNACSRARKWIIDHGGATYIPSWGKTWLSILGVYEWSGSNPMPP
EFWILPTFLPMHPAKMWCYCRMVYMPMSYLYGKRFVGPITPLILQLRQELHTQPYHHINW
TKTRHLCAHEDVYYPHPLIQDLMWDSLYIFTEPLLTRWPFNKIIRKKALEVTMKHIHYED
ENSRYITIGCVEKVLCMLACWAEDPNGVPFKKHLARIPDYMWVAEDGMKMQSFGSQQWDT
GFAIQALLASNLTEEIGQVLKKGHDFIKKSQVKENPSGDFKSMHRHISKGSWTFSDQDHG
WQVSDCTAEGLKCCLLFSMMPPEIVGEKMDAQHLYNAVNILISLQSKNGGLAAWEPAGAQ
QWLEMLNPTEFFADIVIEHEYVECTASAIHALIMFKKLYPGHRKKEIENFITNAVKYLED
VQTADGGWYGNWGVCFTYGTWFAVGGLAAAGKNYNNCAAMRKAVDFLLRTQKQDGGWGES
YLSCPHKKYVPLEDNRSNLVHTSWALMGLISAGQMDRDPTPLHRAAKLLINSQLEDGDF

>PNY2 AB014057 BETA-AMYRIN SYNTHASE [PANAX GINSENG]BAA33722
QADDGHWPAENA
GPLFFLPPLVMCLYITGHLNTVFPAEHRIEILRYIYCHQNDDGGWGLHIEGHSTMFCTAL
SYICMRILGEGRDGGENNACARARKWILDHGSVTAIPSWGKTWLSILGLFDWSGSNPMPP
EFWILPPFLPMHPAKMWCYCRMVYMPMSYLYGKRFVGPITPLILQLREELYAQAYDEINW
RKVRHNCAKEDLYYPHPLIQDLMWDSLYIFTEPFLTRWPFNKLREKALQTTMKHIHYEDE
NSRYITIGCVEKVLCMLACWVEDPNGDYFKQHLARIPDYIWVAEDGMKMQSFGSQEWDTG
FAIQALLASDLIDEIRPTLMKGHDFIKKSQVKENPSGDFKSMHRHISKGSWTFSDQDHGW
QVSDCTAEALKCCLLFSRMPTEIVGDKMEDNQLFDAVNMLLSLQSKNGGLAAWEPAGSSE
WLELLNPTEFFEDIVIEHEYVECTSSAIQAMVMFKKLYPGHRKKEIEVSITNAVQYLEDI
QMPDGSWYGNWGVCFTYGTWFAMGGLTAAGKTYNNCQTLHKAVDFLIKSQRSDGGWGESY
LSCPNKEYTPLEGNRSNLVHTSWAMMGLIHSRQAERDPTPLHRAAKLLINSQMESGDF

>PNY1 AB009030 BETA-AMYRIN SYNTHASE [PANAX GINSENG]BAA33461
QASDGHWPAE
NSGPLFFLPPLVMCVYITGHLDTVFPAEHRKEILRYIYCHQNEDGGWGLHIEGHSTMFCT
TLSYICMRILGEGPDGGVNNACARGRKWILDHGSVTAIPSWGKTWLSILGVYEWIGSNPM
PPEFWILPSFLPMHPAKMWCYCRMVYMPMSYLYGKRFVGPITPLILQLREELYGQPYNEI
NWRKTRRVCAKEDIYYPHPLIQDLLWDSLYVLTEPLLTRWPFNKLREKALQTTMKHIHYE
DENSRYITIGCVEKVLCMLVCWVEDPNGDYFRKHLARIPDYIWVAEDGMKMQSFGSQEWD
TGFSIQALLDSDLTHEIGPTLMKGHDFIKKSQVKDNPSGDFKSMYRHISKGSWTFSDQDH
GWQVSDCTAEGLKCCLIFSTMPEEIVGKKIKPERLYDSVNVLLSLQRKNGGLSAWEPAGA
QEWLELLNPTEFFADIVIEHEYVECTSSAIQALVLFKKLYPGHRKKEIDNFITNAVRYLE
DTQMPDGSWYGNWGVCFTYGSWFALGGLAAAGKTYYNCAAVRKAVEFLLKSQMDDGGWGE
SYLSCPKKVYVPLEGNRSNLVHTGWALMGLIHSEQAERDPTPLHRAAKLLINSQMEDGDF

>KCMS AB257507 MULTIFUNCTIONAL TRITERPENE SYNTHASE [KANDELIA
CANDEL]BAF35580
QASDGHWCAENS
GPMFYVPPMVFALYITGHLTTVFSAEHCKEILRYIYCHQNEDGGWGLHIEGHSTMFCTVL
NYICMRILGEGRDGGKDNACERARKWILDHGSATAISSWGKTWLAILGVYEWDGCNPMPP
EFWVFPTFFPIHPAKMLCYCRLTYIAMSYLYGKKFVGPITPLILQLREEIYNEPYDEINW
SRMRHLCAKEDNHYPHTLTQIILWDAIYLLSEPLLKRWPWSKLRKKALKITIDHIHYEDE
NSRYITIGCVEKPLNMLACWHEDPNGDAFKKHLARISDYVWLAEDGMKIQSFGSQAWDTS

FVLQALIASNLLSETAPTLEKGHNFIKDSQVTENPSGDFRRMFRHISKGSWTFSDKDHGW
QVSDCTAESLKCCLLFSMMPPELVGRKMEPQRVYDAVNVIISLQSKNGGCSAWEQAGAGS
WMEWLNPVEFLEDLVIEHEYIECTSSSVQALVLFKKLYPEHRRKEIENFIVNAVRFIEEI
QKPDGSWYGNWGICFLFGTWFGLKGLATAGKTYYNCTAVRKGVEFLLRTQREDSGWGESY
LSCPKKVYVPLEGNQSNLIHTALAMMGLILSGQAERDPTPLHRALKLLINSQTELGDF

>ATLUP2 NM106545 LUPEOL SYNTHASE [ARABIDOPSIS THALIANA]AT1G78960 NP_178017
QSSDGHWPAEI
TGTLFFLPPLVFCFYITGHLEKIFDAEHRKEMLRHIYCHQNEDGGWGLHIEGKSVMFCTV
LNYICLRMLGEGPNGGRNNACKRARQWILDHGGVTYIPSWGKIWLSILGIYDWSGTNPMP
PEIWLLPSFFPIHLGKTLCYTRMVYMPMSYLYGKRFVGPLTPLIMLLRKELHLQPYEEIN
WNKARRLCAKEDMIYPHPLVQDLLWDTLHNFVEPILTNWPLKKLVREKALRVAMEHIHYE
DENSHYITIGCVEKVLCMLACWIENPNGDHFKKHLARIPDFMWVAEDGLKMQSFGSQLWD
TVFAIQALLACDLSDETDDVLRKGHSFIKKSQVRENPSGDFKSMYRHISKGAWTLSDRDH
GWQVSDCTAEALKCCMLLSMMPAEVVGQKIDPEQLYDSVNLLLSLQGEKGGLTAWEPVRA
QEWLELLNPTDFFTCVMAEREYVECTSAVIQALVLFKQLYPDHRTKEIIKSIEKGVQFIE
SKQTPDGSWHGNWGICFIYATWFALSGLAAAGKTYKSCLAVRKGVDFLLAIQEEDGGWGE
SHLSCPEQRYIPLEGNRSNLVQTAWAMMGLIHAGQAERDPTPLHRAAKLIITSQLENGDF

>ATLUP1 NM106546 LUPEOL SYNTHASE [ARABIDOPSIS THALIANA]AT1G78970 NP_178018
QASDGHWPGEI
TGPLFFLPPLIFCLYITGHLEEVFDAEHRKEMLRHIYCHQNEDGGWGLHIESKSVMFCTV
LNYICLRMLGENPEQDACKRARQWILDRGGVIFIPSWGKFWLSILGVYDWSGTNPTPPEL
LMLPSFLPIHPGKILCYSRMVSIPMSYLYGKRFVGPITPLILLLREELYLEPYEEINWKK
SRRLYAKEDMYYAHPLVQDLLSDTLQNFVEPLLTRWPLNKLVREKALQLTMKHIHYEDEN
SHYITIGCVEKVLCMLACWVENPNGDYFKKHLARIPDYMWVAEDGMKMQSFGCQLWDTGF
AIQALLASNLPDETDDALKRGHNYIKASQVRENPSGDFRSMYRHISKGAWTFSDRDHGWQ
VSDCTAEALKCCLLLSMMSADIVGQKIDDEQLYDSVNLLLSLQSGNGGVNAWEPSRAYKW
LELLNPTEFMANTMVEREFVECTSSVIQALDLFRKLYPDHRKKEINRSIEKAVQFIQDNQ
TPDGSWYGNWGVCFIYATWFALGGLAAAGETYNDCLAMRNGVHFLLTTQRDDGGWGESYL
SCSEQRYIPSEGERSNLVQTSWAMMALIHTGQAERDLIPLHRAAKLIINSQLENGDF

>PNA AB265170 DAMMARENEDIOL-II SYNTHASE [PANAX GINSENG]
QAHDGHWPAE
NAGSLLYTPPLIIALYISGTIDTILTKQHKKELIRFVYNHQNEDGGWGSYIEGHSTMIGS
VLSYVMLRLLGEGLAESDDGNGAVERGRKWILDHGGAAGIPSWGKTYLAVLGVYEWEGCN
PLPPEFWLFPSSFPFHPAKMWIYCRCTYMPMSYLYGKRYHGPITDLVLSLRQEIYNIPYE
QIKWNQQRHNCCKEDLYYPHTLVQDLVWDGLHYFSEPFLKRWPFNKLRKRGLKRVVELMR
YGATETRFITTGNGEKALQIMSWWAEDPNGDEFKHHLARIPDFLWIAEDGMTVQSFGSQL
WDCILATQAIIATNMVEEYGDSLKKAHFFIKESQIKENPRGDFLKMCRQFTKGAWTFSDQ
DHGCVVSDCTAEALKCLLLLSQMPQDIVGEKPEVERLYEAVNVLLYLQSRVSGGFAVWEP
PVPKPYLEMLNPSEIFADIVVEREHIECTASVIKGLMAFKCLHPGHRQKEIEDSVAKAIR
YLERNQMPDGSWYGFWGICFLYGTFFTLSGFASAGRTYDNSEAVRKGVKFFLSTQNEEGG
WGESLESCPSEKFTPLKGNRTNLVQTSWAMLGLMFGGQAERDPTPLHRAAKLLINAQMDN
GDF

>OEA AB291240 MIXED AMYRIN SYNTHASE [OLEA EUROPAEA]
QASDGHWPAE
NAGPMFFTPPLIIVLYISGAINTILTSEHRKEMVRYIYNHQNDDGGWGFYIEGHSTMIGS
ALSYIALRLLGEGPDDGNGSIARARKWILDHGGATGIPSWGKTYLSVLGVYDWDGCNPLP
PEFWLFPSFLPYHPAKMWCYCRTTYMPMSYLYGRKYHGPLTDLVLSLRNEIHIKPYNEID
WNKARHDCCKEDLYYPHSSIQDLLWDTLNYCAEPVMRRWPLNKIRQRALNKTIKYMRYGA
EESRYITIGCVEKSLQMMCWWAHDPNGDEFKHHLARVPDYLWLAEDGMKMQSFGSQIWDS
TLATQAVIATGMVEEYGDCLKKAHFYVKESQIKENPAGDFKSMYRHFTKGAWTFSDQDQG
WVVSDCTAEALKCLLLLSQLPTETAGEKADVERLYEAVNVLLYLQSPESGGFAIWEPPVP
QPYLQMLNPSEIFADIVVETEHVECSASIIQALLAFKRLYPGHREKEIEISVAKAISFLE
GRQWPDGSWYGYWGICFLYGTFFVLGGLSAAGKTYENSEAVRKGVNFLLSTQNEEGGWGE
CLESCPSMKYTPLEGNRTNLVQTSWAMLGLMYGGQAERDPTSLHKAAKLLIDAQMDDGDF

>PSM AB034803 MIXED-AMYRIN SYNTHASE [PISUM SATIVUM]
QSNDGHWPALNA

GPLFYFPPLVFCMYVTGHLDSIFPYEYRKEILRYIYCHQNEDGGWGLHVEGHSIMFCTVL
NYICMRILGEGPNGGKEDACARARKWIHDHGSVTHVSSWGKIWLSVLGIFDWCASNPMPP
EFWMLPSFLLKHPAKMLCYCRLVYMPMSYLYGKRFVGPITPLILMLREELLTQPYEKVNW
KKTRHLCAKEDLYYPHPLIQDLIWDSLYIFVEPLLTHWPFNKLLREKALQTVMKHIHYED
ENSRYITIGCVEKVLCILACWVEDPNGDAFKKHLARLPDYLWVSEDGMTLHSFGSQTWDA
SLIIQALLATNLIEDVGPILTKAHEFIKKSQVRDNPSGDFKSMYRHISKGSWTFSDKDHG
WQVSDCTAESLKCCLLLSMLPPEIVGEKMEPEMLYDSVNILLSLQGKKGGLPAWEPSEAV
EWLELFNPIEFLEEIVVEREYVECTSSAIQALVLFKKLYPEHRKKEVENFIANAVRFLEY
KQTSDGSWYGNWGICFTYGSWFALNGLVAAGKTYDNCAAIRKGVEFLLTTQREDGGWGES
HLSSSKKIYVPLERSQSNIVQTSWAIMGLIHAGQMERDPTPLHRAVKLIINFQQEEGDW

>RCLUS DQ268869 LUPEOL SYNTHASE [RICINUS COMMUNIS]
QASDGHWCAENG
GLLFFLPPLVFAVYITGHLNTVFSPEHRKEILRYIYCHQNEDGGWGIHIEGHSTMFCTVL
NYICMRILGEARDGGIENACERGRKWILDHGGATGISSWGKTWLSILGVYEWDGTNPMPP
EFWAFPSSFPLHPAKMFCYCRITYMPMSYLYGKRFVGPITPLILQIREEIYNEPYNKIKW
NSVRHLCAKEDNYFPHPTIQKLLWDALYTFSEPLFSRWPFNKLREKALKITMDHIHYEDH
NSRYITIGCVEKPLCMLACWIEDPHGEAFKKHLARIADYIWVGEDGIKMQSFGSQTWDTS
LALQALIASDLSHEIGPTLKQGHVFTKNSQATENPSGDFRKMFRHISKGAWTFSDKDQGW
QVSDCTAESLKCCLLFSMMPPEIVGEKMEPEKVYDSVNVILSLQSQNGGFTAWEPARAGS
WMEWLNPVEFMEDLVVEHEYVECTSSAIQALVLFKKLYPRHRNKEIENCIINAAQFIENI
QEPDGSWYGNWGICFSYGTWFALKGLAAAGRTYENCSAIRKGVDFLLKSQRDDGGWAESY
LSCPKKVYVPFEGNRSNLVQTAWAMMGLIYGGQAKRDPMPLHRAAKLLINSQTDLGDF

>ATCAS1 NM126681 CYCLOARTENOL SYNTHASE 1 [ARABIDOPSIS THALIANA]AT2G07050
QAHDGHWPGDYGG
PMFLLPGLIITLSITGALNTVLSEQHKQEMRRYLYNHQNEDGGWGLHIEGPSTMFGSVLN
YVTLRLLGEGPNDGDGDMEKGRDWILNHGGATNITSWGKMWLSVLGAFEWSGNNPLPPEI
WLLPYFLPIHPGRMWCHCRMVYLPMSYLYGKRFVGPITSTVLSLRKELFTVPYHEVNWNE
ARNLCAKEDLYYPHPLVQDILWASLHKIVEPVLMRWPGANLREKAIRTAIEHIHYEDENT
RYICIGPVNKVLNMLCCWVEDPNSEAFKLHLPRIHDFLWLAEDGMKMQGYNGSQLWDTGF
AIQAILATNLVEEYGPVLEKAHSFVKNSQVLEDCPGDLNYWYRHISKGAWPFSTADHGWP
ISDCTAEGLKAALLLSKVPKAIVGEPIDAKRLYEAVNVIISLQNADGGLATYELTRSYPW
LELINPAETFGDIVIDYPYVECTSAAIQALISFRKLYPGHRKKEVDECIEKAVKFIESIQ
AADGSWYGSWAVCFTYGTWFGVKGLVAVGKTLKNSPHVAKACEFLLSKQQPSGGWGESYL
SCQDKVYSNLDGNRSHVVNTAWAMLALIGAGQAEVDRKPLHRAARYLINAQMENGDF

>GGBAS1 AB037203 BETA-AMYRIN SYNTHASE [GLYCYRRHIZA GLABRA]
QTSDGHWPAQIA
GPLFFLPPLVFCMYITGHLDSVFPEEYRKEILRYIYYHQNEDGGWGLHIEGHSTMFCTAL
NYICMRILGEGPDGGQDNACARARKWIHDHGGVTHIPSWGKTWLSILGVFDWCGSNPMPP
EFWILPSFLPMHPAKMWCYCRLVYMPMSYLYGKRFVGPITPLILQLREELFTEPYEKVNW
KKARHQCAKEDLYYPHPLLQDLIWDSLYLFTEPLLTRWPFNKLVREKALQVTMKHIHYED
ETSRYITIGCVEKVLCMLACWVEDPNGDAFKKHLARVPDYLWVSEDGMTMQSFGSQEWDA
GFAVQALLATNLVEEIAPTLAKGHDFIKKSQVRDNPSGDFKSMYRHISKGSWTFSDQDHG
WQVSDCTAEGLKCCLLLSMLPPEIVGEKMEPERLYDSVNVLLSLQSKKGGLSAWEPAGAQ
EWLELLNPTEFFADIVVEHEYVECTGSAIQALVLFKKLYPGHRKKEIENFIANAVRFLED
TQTADGSWYGNWGVCFTYGSWFALGGLAAAGKTFANCAAIRKAVKFLLTTQREDGGWGES
YLSSPKKIYVPLEGSRSNVVHTAWALMGLIHAGQAERDPAPLHRAAKLIINSQLEEGDW

>PNX AB009029 CYCLOARTENOL SYNTHASE [PANAX GINSENG] BAA33460
QAHDGHWPGDYGG
PMFLMPGLVITLSITGVLNVVLSKEHKREICRYLYNHQNRDGGWGLHIEGPSTMFGTVLN
YVTLRLLGEGANDGQGAMEKGRQWILDHGSATAITSWGKMWLSVLGVFEWSGNNPLPPET
WLLPYILPIHPGRMWCHRRMVYLPMSYLYGKRFVGPITPTVLSLRKEVFSVPYHEIDWNQ
ARNLCAKEDLYYPHPLIQDILWASLDKVWEPIFMHWPAKKLREKSLRTVMEHIHYEDENT
RYICIGPVNKVLNMLCCWVEDPNSEAFKLHLPRLHDFLWLAEDGMKMQGYNGSQLWDTAF
AVQAIISTNLAEEYGPTLRKAHTFMKNSQVLDDCPGDLDAWYRHVSKGAWPFSTADHGWP
ISDCTAEGFKAVLQLSKLPSELVGEPLDAKRLYDAVNVILSLQNSDGGYATYELTRSYSW
LELVNPAETFGDIVIDYPYVECTSAAIQALTAFKKLFPGHRREEIQHSIEKAALFIEKIQ
SSDGSWYGSWGVCFTYGTWFGIKGLVTAGRTFSSCASIRKACDFLLSKQVASGGWGESYL

SCQNKVYTNLEGNRSHVVNTGWAMLALIDAGQAERDATPLHRAAKLLINSQMENGDF

>PSY AB034802 BETA-AMYRIN SYNTHASE [PISUM SATIVUM] BAA97558
QTSDGHWPAQIA
GPLFFMPPLVFCVYITGHLDSVFPPEHRKEILRYIYCHQNEDGGWGLHIEGHSTMFCTAL
NYICMRILGEGPDGGEDNACVRARNWIRQHGGVTHIPSWGKTWLSILGVFDWLGSNPMPP
EFWILPSFLPMHPAKMWCYCRLVYMPMSYLYGKRFVGPITPLILQLREELHTEPYEKINW
TKTRHLCAKEDIYYPHPLIQDLIWDSLYIFTEPLLTRWPFNKLVRKRALEVTMKHIHYED
ENSRYLTIGCVEKVLCMLACWVEDPNGDAFKKHIARVPDYLWISEDGMTMQSFGSQEWDA
GFAVQALLATNLIEEIKPALAKGHDFIKKSQVTENPSGDFKSMHRHISKGSWTFSDQDHG
WQVSDCTAEGLKCCLLLSLLPPEIVGEKMEPERLFDSVNLLLSLQSKKGGLAAWEPAGAQ
EWLELLNPTEFFADIVVEHEYVECTGSAIQALVLFKKLYPGHRKKEIENFIFNAVRFLED
TQTEDGSWYGNWGVCFTYGSWFALGGLAAAGKTYTNCAAIRKGVKFLLTTQREDGGWGES
YLSSPKKIYVPLEGNRSNVVHTAWALMGLIHAGQSERDPTPLHRAAKLLINSQLEQGDW

>PSX D89619 CYCLOARTENOL SYNTHASE [PISUM SATIVUM] BAA23533
QSHDGHWPGDYGG
PMFLMPGLVITLSVTGALNAVLTDEHRKEMRRYLYNHQNKDGGWGLHIEGPSTMFGSVLC
YVTLRLLGEGPNDGEGDMERGRDWILEHGGATYITSWGKMWLSVLGVFEWSGNNPMPPEI
WLLPYALPVHPGRMWCHCRMVYLPMSYLYGKRFVGPITPTVLSLRKELFTVPYHDIDWNQ
ARNLCAKEDLYYPHPLVQDILWATLHKFVEPVFMNWPGKKLREKAIKTAIEHIHYEDENT
RYICIGPVNKVLNMLCCWVEDPNSEAFKLHLPRIYDYLWVAEDGMKMQGYNGSQLWDTAF
AAQAIISTNLIDEFGPTLKKAHAFIKNSQVSEDCPGDLSKWYRHISKGAWPFSTADHGWP
ISDCTAEGLKAVLLLSKIAPEIVGEPLDSKRLYDAVNVILSLQNENGGLATYELTRSYTW
LEIINPAETFGDIVIDCPYVECTSAAIQALATFGKLYPGHRREEIQCCIEKAVAFIEKIQ
ASDGSWYGSWGVCFTYGTWFGIKGLIAAGKNFSNCLSIRKACEFLLSKQLPSGGWAESYL
SCQNKVYSNLEGNRSHVVNTGWAMLALIEAEQAKRDPTPLHRAAVCLINSQLENGDF

>AT1G66960 AF489920 MULTIFUNCTIONAL TRITERPENE SYNTHASE [ARABIDOPSIS THALIANA] F1019.4
QASDGHWPGEF
TGPLCMLPPLVFCLYITGHLEEVFDAEHRKEMLRYIYCHQNEDGGWGFHIESKSIMFTTT
LNYICLRILGVGPDGGLENACKRARQWILSHGGVIYIPCWGKVWLSVLGIYDWSGVNPMP
PEIWLLPYFLPIHLGKAFSYTRITYMPISYLYGKKFVGQITPLIMQLREELHLQPYEEIN
WNKARHLCAKEDKYYPHPLVQDLIWDALHTFVEPLLASWPINKLVRKKALQVAMKHIHYE
DENSHYITIGCIEKNLCMLACWIDNPDGNHFKKHLSRIPDMMWVAEDGMKMQCFGSQLWM
TGFAVQALLASDPRDETYDVLRRAHDYIKKSQVRDNPSGDFKSMYRHISKGGWTLSDRDH
GWQVSDCTAEAAKCCMLLSTMPTDITGEKINLEQLYDSVNLMLSLQSENGGFTAWEPVRA
YKWMELMNPTDLFANAMTEREYTECTSAVLQALVIFNQLYPDHRTKEITKSIEKAVQFIE
SKQLRDGSWYGSWGICFTYGTWFALCGLAAIGKTYNNCLSMRDGVHFLLNIQNEDGGWGE
SYMSCPEQRYIPLEGNRSNVVQTAWAMMALIHAGQAKRDLIPLHSAAKFIITSQLENGDF

>AT5G42600 BT020312 PUTATIVE PENTACYCLIC TRITERPENE SYNTHASE [ARABIDOPSIS THALIANA] AT5G42600
QANDGHWPSEV
SGSMFLDAPFVICLYITGHLEKIFTLEHVKELLRYMYNTQNEDGGWGLDVESHSVMFCTV
LNYICLRILGVEPDHDGQKSACARARKWILDHGGATYAPMVAKAWLSVLGVYDWSGCKPL
PPEIWMLPSFSPINGGTLWIYIRDLLMGMSYLYGKKFVATPTALILQLREELYPQPYSKI
IWSKARNRCAKEDLLYPKSFGQDLFWEGVHMLSENIINRWPLNKFVRQRALRTTMELVHY
HDETTHYITGACVAKPFHMLACWVEDPDGDYFKKHLARVPDFIWIAEDGLKFQLMGMQSW
NAALSLQVMLAANMDDEIRSTLIKGYDFLKQSQISENPQGDHLKMFRDITKGGWTFQDRE
QGLPISDGTAESIECCIHFHRMPSEFIGEKMDVEKLYDAVNFLIYLQSDNGGMPVWEPAP
GKKWLEWLSPVEHVENTVVEQEYLECTGSVIAGLVCFKKEFPDHRPKEIEKLIKKGLKYI
EDLQMPDGSWYGNWGVCFTYGTLFAVRGLAAAGKTFGNSEAIRRAVQFILNTQNAEGGWG
ESALSCPNKKYIPSKGNVTNVVNTGQAMMVLLIGGQMERDPSPVHRAAKVLINSQLDIGD
F
>AT4G15370 NM117625 PUTATIVE PENTACYCLIC TRITERPENE SYNTHASE [ARABIDOPSIS THALIANA]
QADDGHWPAEN
AGSIFFNAPFVICLYITGHLEKIFTHEHRVELLRYMYNHQNEDGGWGLHVESPSNMFCSV
INYICLRILGVEAGHDDKGSACARARKWILDHGGATYSPLIGKAWLSVLGVYDWSGCKPI

PPEFWFLPSFFPVNGGTLWIYLRDIFMGLSYLYGKNFVATSTPLILQLREEIYPEPYTNI
SWRQARNRCAKEDLYYPQSFLQDLFWKGVHVFSENILNRWPFNNLIRQRALRTTMELVHY
HDEATRYITGGSVPKVIAVFHMLACWVEDPESDYFKKHLARVPDFIWIGEDGLKIQSFGS
QVWDTALSLHVFIDGFDDDVDEEIRSTLLKGYDYLEKSQVTENPPGDYMKMFRHMAKGGW
TFSDQDQGWPVSDCTAESLECCLFFESMSSEFIGKKMDVEKLYDAVDFLLYLQSDNGGIT
AWQPADGKLVEFIEDAVVEHEYVECTGSAIVALAQFNKQFPGYKKEEVERFITKGVKYIE
DLQMVDGSWYGNWGVCFIYGTFFAVRGLVAAGKCYNNCEAIRRAVRFILDTQNTEGGWGE
SYLSCPRKKYIPLIGNKTNVVNTGQALMVLIMGNQMKRDPLPVHRAAKVLINSQMDNGDF

>AT1G78500_NM106497 MULTIFUNCTIONAL PENTACYCLIC TRITERPENE SYNTHASE [ARABIDOPSIS THALIANA]
QAEDGHWPAEN
SGCLFFEAPFVICLYITGHLEKILTLEHRKELLRYMYNHQNEDGGWGIHVEGQSAMFCTV
INYICLRILGVEADLDDIKGSGCARARKWILDHGGATYTPLIGKAWLSILGVYDWSGCKP
IPPEVWMLPTFSPFNGGTLWIYFRDIFMGVSYLYGKKFVATPTPLILQLREELYPQPYDK
ILWSQARNQCAKEDLYYPQSFLQEMFWKCVHILSENILNRWPCNKLIRQKALRTTMELLH
YQDEASRYFTGGCVPKPFHMLACWVEDPDGDYFKKHLARVPDYIWIGEDGLKIQSFGSQL
WDTAFSLQVMLAYQDVDDDDDEIRSTLIKGYSFLNKSQLTQNPPGDHRKMLKDIAKGGWT
FSDQDQGWPVSDCTAESLECCLVFGSMPSELIGEKMDVERLYDAVNLLLYFQSKNGGITV
WEAARGRTWLEWLSPVEFMEDTIVEHEYVECTGSAIVALARFLKEFPEHRREEVEKFIKN
AVKYIESFQMPDGSWYGNWGVCFMYGTFFAVRGLVAAGKTYQNCEPIRKAVQFILETQNV
EGGWGESYLSCPNKKYTLLEGNRTNVVNTGQALMVLIMGGQMERDPLPVHRAAKVLINSQ
LDNGDF

>AT5G48010 NM124175 PUTATIVE PENTACYCLIC TRITERPENE SYNTHASE [ARABIDOPSIS THALIANA]
QADDGHWPAEN
SGPNFYTPPFLICLYITGHLEKIFTPEHVKELLRHIYNMQNEDGGWGLHVESHSVMFCTV
INYVCLRIVGEEVGHDDQRNGCAKAHKWIMDHGGATYTPLIGKALLSVLGVYDWSGCNPI
PPEFWLLPSSFPVNGGTLWIYLRDTFMGLSYLYGKKFVAPPTPLILQLREELYPEPYAKI
NWTQTRNRCGKEDLYYPRSFLQDLFWKSVHMFSESILDRWPLNKLIRQRALQSTMALIHY
HDESTRYITGGCLPKAFHMLACWIEDPKSDYFKKHLARVREYIWIGEDGLKIQSFGSQLW
DTALSLHALLDGIDDHDVDDEIKTTLVKGYDYLKKSQITENPRGDHFKMFRHKTKGGWTF
SDQDQGWPVSDCTAESLECCLFFESMPSELIGKKMDVEKLYDAVDYLLYLQSDNGGIAAW
QPVEGKAWLELLNIMIFRYVECTGSAIAALTQFNKQFPGYKNVEVKRFITKAAKYIEDMQ
TVDGSWYGNWGVCFIYGTFFAVRGLVAAGKTYSNCEAIRKAVRFLLDTQNPEGGWGESFL
SCPSKKYTPLKGNSTNVVQTAQALMVLIMGDQMERDPLPVHRAAQVLINSQLDNGDF

>AT4G15340(ATPEN1) NM117622 PENTACYCLIC TRITERPENE SYNTHASE 1 [ARABIDOPSIS THALIANA]
QADDGHWPADN
SGPNFFIAPLVICLYITGHLEKIFTVEHRIELIRYMYNHQNEDGGWGLHVESPSIMFCTV
INYICLRIVGVEAGHDDDQGSTCTKARKWILDHGGATYTPLIGKACLSVLGVYDWSGCKP
MPPEFWFLPSSFPINGGTLWIYLRDIFMGLSYLYGKKFVATPTPLILQLQEELYPEPYTK
INWRLTRNRCAKEDLCYPSSFLQDLFWKGVHIFSESILNRWPFNKLIRQAALRTTMKLLH
YQDEANRYITGGSVPKAFHMLACWVEDPEGEYFKKHLARVSDFIWIGEDGLKIQSFGSQL
WDTVMSLHFLLDGVEDDVDDEIRSTLVKGYDYLKKSQVTENPPSDHIKMFRHISKGGWTF
SDKDQGWPVSDCTAESLKCCLLFERMPSEFVGQKMDVEKLFDAVDFLLYLQSDNGGITAW
EPADGKTWLEWFSPVEFVQDTVIEHEYVECTGSAIVALTQFSKQFPEFRKKEVERFITNG
VKYIEDLQMKDGSWCGNWGVCFIYGTLFAVRGLVAAGKTFHNCEPIRRAVRFLLDTQNQE
GGWGESYLSCLRKKYTPLAGNKTNIVSTGQALMVLIMGGQMERDPLPVHRAAKVVINLQL
DNGDF

>AT5G36150(ATPEN3) NM123006 PENTACYCLIC TRITERPENE SYNTHASE 3 [ARABIDOPSIS THALIANA]
QSDDGHWPSEN
AGCIFFNAPFVICLYITGHLDKVFSEEHRKEMLRYMYNHQNDDGGWGIDVESHSFMFCTV
INYICLRIFGVDPDHDGESACARARKWIIDHGGATYTPLFGKAWLSVLGVYEWSGCKPIP
PEFWFFPSYFPINGGTLWIYLRDTFMAMSYLYGKKFVAKPTPLILQLREELYPQPYAEIV
WSQARSRCAKEDLYYPQSLVQDLFWKLVHMFSENILNRWPFNKLIREKAIRTAMELIHYH
DEATRYITGGAVPKVFHMLACWVEDPESDYFKKHLARVSHFIWIAEDGLKIQTFGSQIWD

TAFVLQVMLAADVDDEIRPTLIKGYSYLRKSQFTENPPGDYINMFRDISKGGWGYSDKDQ
GWPVSDCISESLECCLIFESMSSEFIGEKMEVERLYDAVNMLLYMQSRNGGISIWEAASG
KKWLEWLSPIEFIEDTILEHEYLECTGSAIVVLARFMKQFPGHRTEEVKKFITKGVKYIE
SLQIADGSWYGNWGICFIYGTFFAVRGLVAAGNTYDNCEAIRRAVRFLLDIQNGEGGWGE
SFLSCPNKNYIPLEGNKTDVVNTGQALMVLIMGGQMDRDPLPVHRAAKVLINSQMDNGDF

>LJAMY2 AF478455 MULTIFUNCTIONAL BETA-AMYRIN SYNTHASE [LOTUS JAPONICUS]
QTSDGHWPAQIA
GPLLFTPPLIFCMYITGHLDSVFPEVYRKEILRYTYVHQNEDGGWGLHIEGHSTMFCTVL
NYICMRILGEGPDGGQDNACARARKWIHDHGGATHIASWGKTWLSILGIFDWSGTNPMPP
EFWILPSFLPMHPAKMWCYCRLVYMPMSYLYGKRFVGPITPLILQLREELFTQPYEKVNW
KKARHQCAKEDLYYPHPLIQDLMWDSLYLFTEPFLTRWPFNKLIRERALQVTMKHIHYED
HNSRYITIGCVEKVLCMLACWVEDPNGIAFKRHLARVPDYLWLAEDGMCMQSFGSQEWDA
GFAVQALLSTNLIDELGPALAKGHDFIKNSQVKDNPSGDFKSMHRHISKGAWTFSDQDHG
WQVSDCTAEGFKCCLLLSMLPPEIVGEKIEPERLFDTVNLLLSLQSKKGGFAVWEPAGAQ
EWLELLNPIEFFEDIVIEHELVECTGSAIGALVLFKNHYPEHRKKEIEDCIANAVRYFED
IQTADGSWYGNAGICFIYGTWFALGGLEAAGKTYANCAAIRKGVKFLLTTQSKDGGWGES
YLSCPKKIYVPLEGNRSNVVQTAWALMGLIHAGQAERDPTPLHRAAKLLINSQLEDGDW

>LJAMY1 AB181244 BETA-AMYRIN SYNTHASE [LOTUS JAPONICUS]
QTSDGHWPAQIA
GPLFFQPPLVFCMYITGHLNSVFPEEYRKEILRYIYVHQNEDGGWGLHIEGHSTMFCTAL
NYICMRMLGEGPDGGQDNACARARKWILDHGGVTHIPSWGKTWLSILGIFDWKGSNPMPP
EFWILPSFLPMHPAKMWCYCRLVYMPMSYLYGKRFVGPITPLILQLREELFTQPYEKVNW
KKARHQCAKEDIYYPHPLIQDLMWDSLYLFTEPLLTRWPFNKLVREKALEVTMKHIHYED
ENSRYITIGCVEKVLCMLACWVEDPNGDAFKKHLARIPDYLWVSEDGMCMQSFGSQEWDA
GFAVQALLATNLVDELGPTLAKGHDFIKKSQVRDNPSGDFKNMHRHISKGSWTFSDQDHG
WQVSDCTAEGLKCCLLLSMLPPDIVGEKMEPECLFDSVNLLLSLQSKKGGLAAWEPAGAQ
EWLELLNPTEFFADIVVEHEYVECTGSAIGALVLFKKLYPGHRKKEIENFISEAVRFLED
TQTADGSWYGNWGVCFTYGSWFALGGLAAAGKTYANCAAIRKAVKFLLTTQRGDGGWGES
YLSSPKKIYVPFEGNRSNVVHTAWALMGLIHSGQAERDPTPLHRAAKLLINSQLEEGDW

>MTAMY1 AF478453 BETA-AMYRIN SYNTHASE [MEDICAGO TRUNCATULA]
QTSDGHWPAQIA
GPLFFMPPLVFCVYITGHLDSVFPREHRKEILRYIYCH
QNEDGGWGLHIEGHSTMFCTAL
NYICMRILGEGPDGGQDNACARARNWIRAHGGVTYIPSWGKTWLSILGLFDWLGSNPMPP
EFWILPSFLPMHPAKMWCYCRLVYMPMSYLYGKRFVGPITPLILQLREELHTQPYEKINW
TKSRHLCAKEDIYYPHPLIQDLIWDSLYIFTEPLLTRWPFNKLVRKRALEVTMKHIHYED
ENSRYLTIGCVEKVLCMLACWVEDPNGDAYKKHLARVQDYLWMSEDGMTMQSFGSQEWDA
GFAVQALLAANLNDEIEPALAKGHDFIKKSQVTENPSGDFKSMHRHISKGSWTFSDQDHG
WQVSDCTAEGLKCCLLLSMLPPEIVGEKMEPERLYDSVNVLLSLQSKKGGLAAWEPAGAQ
EWLELLNPTEFFADIVVEHEYVECTGSAIQALVLFKKLYPGHRKKEIENFISEAVRFIED
IQTADGSWYGNWGVCFTYGSWVALGGLAAAGKTYTNCAAIRKAVKFLLTTQREDGGWGES
YLSSPKKIYVPLEGSRSNVVHTAWALMGLIHAGQAERDPTPLHRAAKLLINSQLEEGDW

>MDOSC1 EST172892
QSPHGHWPAENA
GPNFYFPPLVMAAYIPGYLNVIFSAEHKKEILRYTYNHQNEDGGWGLHIAGPSMMFTTCL
NYCMMRILGDGPDGGRDNACARARKWILDRGGAYYSASWGKTWMAILGVYDWEGSNPMPP
EFWTGSTLLPFHPSKMFCYCRLTYLPMSYFYATRFVGPITPLVEELRQEIYCESYNEINW
PKVRHWCATEDNYYPHGRVQRFMWDGFYNIVEPLLKRWPFKKIRDNAIQFTIDQIHYEDE
NSRYITIGCVEKPLMMLACWAEDPSGEAFKKHLPRVTDYIWLGEDGIKMQSFGSQSWDCA
LVIQALLAGNLNAEMGPTLKKAHEFLKISQVRINTSGDYLSHFRHISKGAWTFSDRDHGW
QVSDCTAEALRCCCIFANMSPEVVGEPMEAECMYDAVNVIMSLQSPNGGVSAWEPTGAPK
WLEWLNPVEFLEDLVIEYEYIECTSSSIQALTLFRKLYPGHRRKEINNFITRAADYIEDI
QYPDGSWYGNWGICFVYGTWFAIKGLEAAGRTYNNCEAVRKGVDFLLKTQRADGGWGEHY
TSCTNKKYTAQDSTNLVQTALGLMGLIHGRQAERDPTPIHRAAAVLMNGQLDDGDF

>MDOSC2 EST186376
QASDGHWPAENA

GPLFFLPPLVMCVYITGHLNTVFHAEHRKEILRYIYYHQNQDGGWGLHIEGHSTMFCTAL
SYICMRILGEGPDGGQDNACARARKWILDHGSVTHIPSWGKTWLSILGVFEWSGSNPMPP
EFWILPSFLPMHPAKMWCYCRMVYMPMSYLYGKRFVGPITPLILQLREELYAQPYDEINW
KGVRHHCAKEDIYYPHPWIQDFLWDSLYICTEPLLTRWPFNKLIRKRALDVTMKHIHYED
ENSRYITIGCVEKVLCMLACWAEDPNGDYFKKHLARIPDYLWVAEDGLKMQSFGSQLWDT
GCAIQALLASNLTDEIAPTLARGHDFVKKSQVKDNPSGDFKSMHRHISKGSWTFSDQDHG
WQVSDSTADGLKCCLLLSMMPPEMVGEQMEPERLYDAVNVIISLQSKNGGLAAWEPAGAA
DWLEMLNPTEFFADIVVEHEYVECTSSAIQALVLFKKLYPGHRKKEIDQFITNAAQYLEN
TQMADGSWYGNWGVCFTYGTWFALGGLTAAGKTFNNCAVIRKAISFLLTIQKENGGWGES
YLSCPKKEYVPLEGNRSNLVHTAWAMMGLIHAGQAERDPTPLHRAAKLIINSQMENGDF

>MDOSC3 EST246215
QSPHGHWPAENA
GPNFYFPPLVMAAYIPGYLNVIFSAEHKKEILRYTYNHQNEDGGWGLHISGPSMMFTTCL
NYCMMRILGEGPDGGRDNACARARKWILDRGGAYYSASWGKTWMAILGVYDWEGSNPMPP
EFWTGSTLLPFHPSKMFCYCRLTYLPMSYFYATRFVGPITPLVEELRQEIYCEPYSEINW
SKVRHWCAPDDNYYPHGRVQRFMWDSFYNIAEPLLKRWPFKKIRDNAIQFTIDQIHYEDE
NSRYITIGCVEKPLMMLACWAEDPSGEAFKKHLPRVTDYIWLGEDGIKMQSFGSQSWDCA
LVIQALLAGNLNTEMAPTLKKAHEFLKISQVRVNTSGDYLAHFRHVSKGAWTFSDRDHGW
QVSDCTAEALRCCCIFANMSPELVGEPMEAECMYDAVNVILTLQSPNGGVSAWEPTGAPK
WLEWLNPVEFLEDLVIEYEYIECTSSSIQALTLFRKLYPGHRRKEINNFITRAADYIEDI
QYPDGSWYGNWGICFVYGTWFAIKGLEAAGRTYNNCEAVRKGVDFLLKTQRADGGWGEHY
TSCTNKKYTAQDSTNLVQTALGLMGLIHGRQAERDPTPIHRAAAVLMKGQLDDGDF

>MDOSC4 AMS36882.1 [MALUS DOMESTICA]
QASDGHWPAENAGPLFFLPPLVMCVYITGHLN
TVFHAEHRKEILRYIYYHQNQDGGWGLHIEGHSTMFCTALSYICMRILGEGPDGGQDNACARARKWILDH
GSVTHIPSWGKTWLSILGVFEWSGSNPMPPEFWILPSFLPMHPAKMWCYCRMVYMPMSYLYGKRFVGPIT
PLILQLREELYAQPYDEINWKGVRHHCAKEDIYYPHPWIQDFLWDSLYICTEPLLTRWPFNKMIRKRALE
VTMKHIHYEDENSRYITIGCVEKVLCMLACWAEDPNGDYFKKHLARIPDYLWVAEDGMKMQSFGSQQWDT
GFAIQALLASNLTDEIAPTLARGHDFVKKSQVKDNPSGDFKSMHRHISKGSWTFSDQDHGWQVSDCTAEG
LKCCLLLSMMPPELVGEKMEPERLYDAVNVLISLQSKNGGLAAWEPAGAAEWLEMLNPTEFFADIVVEHE
YVECTSSAIQALVLFKKLYPGHRKKEIDQFITNAAQYLENIQMEDGSWYGNWGVCFTYGTWFALGGLTAA
GKTFNNCAVIRKAINFLLTIQKENGGWGESYLSCPKKEYVPLEGNRSNLVHTAWAMMGLIYAGQAERDPA
PLHRAAKLIMNSQMENGDF

>MDOSC5 AMS36883.1 [MALUS DOMESTICA]
QASDGHWPAENAGPLFFLPPLVMCTYITGHLN
TVFPTEHRKEILRYIYYHQNQDGGWGLHIEGHSTMFCTALNYICMRILGEGPDGGQDKACARARKWILDH
GSVTHIPSWGKTWLSILGVFEWSGSNPMPPEFWILPSFLPMHPAKMFCYSRMVYMPMSYLYGKRFVGPIT
PLILQLREELYAQPYGEINWKGVRRHCAKVDIYYPHPWIQDFLWDSLYVCTEPLLTRWPFNKLIRKRALE
VTMEHVHYEDENSRYITIGCVEKVLCMLACWVEDPNGDYFKKHLARIPDYLWVAEDGMKMQSFGSQQWDT
GFAIQALLASNLTDEIAPTLARGHDFVKKSQVKDNPSGDFKSMYRHISKGSWTFSDQDHGWQVSDCTAEG
LKCCLLLSMMPPEMVGEKMEPERLFDAVNVLISLQSKNGGLAAWEPAGAAEWLEMFNPSEFFADIVVEHE
YVECTSSAIQALVLFKKLCPGHRKEEIDQFITAAALYLENVQMADGSWYGNWGVCFTYGTWFALGGLTAA
GKTFNNCAAMRKAISFLLAIQKENGGWGESYLSCPKKEYVPLEGNRSNLVHTAWAMMGLIHAGQAERDPT
PLHRAAKLIINSQMENGDF

>AMCAS1 AF216755 CYCLOARTENOL SYNTHASE [ABIES MAGNIFICA]
QAHDGHWPGDYGG
PMFLMPGLVIALYVTGALNAVLSEMHKKEICRYLYNHQNEDGGWGLHIEGHSTMFGTVLN
YVTLRLLGQAPDGGQGAMEKGCAWILDHGGATAIPSWGKMWLSVLGVFDWTGNNPLPPEM
WLLPYFLPTHPGRMWCHCRMVYLPMSYIYGRRFVGPLTGIVMSLREELYTVPYEKIDWNQ
ARSMCAKEDLYYPHPFLQDILWGTLHKVVEPALMHWPGSMLRERALQSVMKHIHYEDENT
RYICIGPVNKVLNMLCCWVEDSNSEAFKRHLARVVDYLWVAEDGMKMQGYNGSQLWDTAF
ATQALISTNLLDDCGPLLKKAHIYIERSQVQEDCPGDLNFWYRHISNGAWPFSTRDHGWP
ISDCSSEGLKAALALSQLPQDIVGKPIPSQRIFDCVNLMLSMQNSDGGFATYELTRSYPW
LEKINPAETFGDIVIDYSYVECTSAITQALVSFKKLYPEHRHKEIETCILKATRYIENIQ
RPDGSWYGSWGVCFTYGTWFGVLGLAAAGKTYQNCSNIRKACEFLLSKQLPSGGWGESYL
SCQEKVYTHLEGGRSHIVSTAWAMLALIYAGQALRDPKPLHRAAIVLVNYQMENGDF

>CASBPX1 AB055509 CYCLOARTENOL SYNTHASE [BETULA PLATYPHYLLA]
QADDG
HWPGDYGGPMFLIPGLVITLSITGTLNAFLSKEHQCEICRYLYNHQNEDGGWGLHIEGPS
TMFGTALNYITLRLLGEPEDGMGAVEKARKWILDHGGATAITSWGKMWLSVLGVYEWSGN
NPLPPEVWLCPYLLPCHPGRMWCHCRMVYLPMSYLYGKRFVGPITSTIQSLRKELYTVPY
HEIDWNKARNDCAKEDLYYPHPLVQDILWASLYYAYEPIFMYWPAKRLREKALDTVMQHI
HYEDENTRYICIGPVNKVLNMLCCWAEDPNSEAFKLHLPRILDYLWIAEDGMKMQGYNGS
QLWDTTFAVQAIISTNIAEEYGQTLRKAHEYIKDSQVLEDCPGDLNFWYRHISKGAWPFS
TADHGWPISDCTAEGLKAVILLSQFPSETVGKSVDVKRLYDAVHVILSLQNTDGGFATYE
LTRSYHWLELINPAETFGDIVIDYPYVECTSAAIQALTLFKKLHPGHRREEIENCIAKAA
EFIENIQASDGSWYGSWGVCFTYAGWFGIKGLVAAGRTYKNCSSIHKACDYLLSKELASG
GWGESYLSCQDKVYTNLKDNRPHIVNTGWAMLALIDAGQAERDPTPLHRAARILINSQME
NGDF

>CASBPX2 AB055510 CYCLOARTENOL SYNTHASE [BETULA PLATYPHYLLA]
QAHDGHWAGDYGG
PMFLMPGLVITLSITGALNTVLSEEHKKEMCRYLYNHQNKDGGWGLHIEGPSTMFGTVLS
YVTLRLLGEGANDGQGAIERGRKWILDHGSATAIISWGKMWLSVLGAFEWSGNNPLPPEI
WLLPYMLPVHPGRMWCHCRMVYLPMSYLYGKRFVGPITPTVMSLRKELYSVPYHEIDWNQ
ARNLCAKEXLYYPHPLVQDILWASLHKLVEPVLMRWPGKRLREKALRTVLEHIHYEDENT
RYICIGPVNKVLNMLCCWVEDPNSEAFKLHLPRINDYLWIAEDGMKMQGYNGSQLWDTAF
AVQAIISTNLFEEYGPTLEKAHMYIKKSQVREDCPGDLDFWYRHISKGAWPFSTADHGWP
ISDCTAEGLKAALLLSKIPPDVVGEPLVEERLYDAVNVILSLQNADGGFATYELTRSYPW
LELINPAETFGDIVIDYNYVECTSAAIQALTSFKKSYPKHREEEVDVCIKRAAMFTEKIQ
ASDGSWYGSWGVCFTYGTWFGVKGLVAAGKNFNDCFGIRKACDFLLSKQLPSGGWGESYL
SCQNKVYSHVEGNRSHVVNTGWAMLALIEAGQAERDPTPLHRAARVLINSQMENGDF

>GGCAS1 AB025968 CYCLOARTENOL SYNTHASE [GLYCYRRHIZA GLABRA]
QSHDGHWPGDYGG
PMFLMPGLVITLSITGALNAVLTEEHRKEICRYLYNHQNKDGGWGLHIEGPSTMFGSVLN
YVALRLLGEGPNDRQGEMEKGRDWILGHGGATFITSWGKMWLSVLGVYEWSGNNPLPPEI
WLLPYVLPIHPGRMWCHCRMVYLPMSYLYGKRFVGPITPTILSLRKELYTIPYHDIDWNQ
ARNLCAKEDLYYPHPLVQDILWASLHKFLEPILMHWPGKKLREMAIKTAIEHIHYEDDNT
RYLCIGPVNKVLNMLCCWVEDPNSEAFKLHLPRIYDYLWIAEDGMKMQGYNGSQLWDTAF
TAQAIISSNLIEEYGPTLRKAHTYIKNSQVLEDCPGDLSKWYRHISKGAWPFSTADHGWP
ISDCTAEGLKAVLLLSKIAPEIVGEPLDAKRLYDAVNVILSLQNEDGGFATYELTRSYTW
LELINPAETFGDIVIDYPYVECTSAAIQALTSFKKLYPGHRREEIQCCIEKAASFIEKTQ
ASDGSWYGSWGVCFTYGTWFGVKGLIAAGKSFNNCSSIRKACEFLLSKQLPSGGWGESYL
SCQNKVYSNVESNRSHVVNTGWAMLALIDAEQAKRDPTPLHRAAVYLINSQMENGDF

>CSOSC1 AB058507 CYCLOARTENOL SYNTHASE [COSTUS SPECIOSUS]
QAHDGHWPGDYGG
PMFLMPGLIITLYVTGALNTVLTSEHQKEIRRYLYNHQNEDGGWGLHIEGESTMFGSALT
YVILRLLGEGPDDGDGAMEKGRKWILDHGSATAITSWGKMWLSVLGVFDWSGNNPLPPEM
WLLPYFLPVHPGRMWCHCRMVYLPMSYIYGKRFTGPITPLILSLRKELFNLPFDQLDWNK
ARNECAKEDLYYPHPFIQDVLWASLHKFVEPILMHWPGSKLREKAVNTAMQHVHYEDENT
RYICIGPVNKVLNMLCCWIEDPNSEAFKLHLPRVMDYLWLAEDGMKMRGYNGSQLWDTAF
TVQAIISTDLFEEFGLALTKAHEFIKKTQVLEDCPGDLNFWYRHISKGAWPFSTADHGWP
ISDCTSEGLKAALLLSKISPEIVGDPLDGKSLYDAVNVILSLMNNDGGFATYELTRSYAW
LEIINPAETFGDIVIDYPYVECTSAAIQALTLFKKTYPGHRREEIDNCIRKSARFIEKIQ
LADGSWYGSWGVCFTYGIWFGMKGLLAAGRTYETSSCIRKACDFLLSKQVASGGWGESYL
SCQNKVYTNLEGNRAHAVNTGWAMLALIDAGQGERDPKPLHRAAKVLINMQMENGEF

>CACAS AY520819 CYCLOARTENOL SYNTHASE [CENTELLA ASIATICA]
QANDGHWAGDYGG
PMFLMPGLVITLSITEALNAILSKEHKREICRYLYNHQNRDGGWGLHIEGPSTMFGSVLN
YVTLRLLGEGTKDGQGAMEKGRQWILDHGGATAITSWGRMWLSVLGVFEWSGNNPLPPEI
WLFPYNLPFHPGRMWCHCRMVYLPMSYLYGKRFVGPITPTVLSLRKELFTVPYHEIDWNQ
ARNLCAKEDLYYPHPLVQDILWASIDKVLEPILMRWPGKKLREKALRTVMEHIHYEDENT
RYICIGPVNKVLNMLCCWAEDPNSEAFKLHLPRLYDFLWLAEDGMKMQGYNGSQLWDTAF
AVQAIISTSLTEYCGPTLRKAHTFMKDSQVLDDCPGDLDFWYRHISKGAWPFSTADHGWP

ISDCTAEGFKAVLLLSKFPAELVGEPLDAKRLYDAVNVILSLQNSDGGYATYELTRSYRW
LELINPAETFGDIVIDYPYAECTSAAIQALSAFKKLYPGHRREEIQCSIEKAADFIEKIQ
ASDGSWYGSWGVCFTYGTWFGVKGLISAGRTFSNCSSIRKACYFLLSKQLASGGWGESYL
SCQNKVYTNLEGERSHVVNTGWAMLALIEAGQAERDATPLHHAAKLLINSQMENGDF

>LCCAS1 AB033334 CYCLOARTENOL SYNTHASE [LUFFA CYLINDRICA]
QGDDG
HWPGDYG-GPM-FLIPGLVITLSITGALNAVLSTEHQREICRYLYNHQNKDGGWGLHIEG
PSTMFGSVLNYVSLRLLGEEAEDGQGAVDKARKWILDHGGASAITSWGKMWLSVLGVYEW
AGNNPLPPELWLLPYLLPFHPGRMWCHCRMVYLPMCYLYGKRFVGPITPIIRSLRKELYL
VPYHEVDWNKARNECAKEDLYYPHPLVQDIVWASLHHVYEPLFMRWPAKRLREKALQCVM
QHIHYEDENTRYICIGPVNKVLNMLCCWVEDPHSEAFKLHIPRIFDYLWIAEDGMKMQGY
NGSQLWDTAFAVQAIMSTKLAEEYGTTLRKAHKYIKDSQVLEDCPGDLQSWYR-HISKGA
WPFSTADHGWPISDCTAEGLKAVLLLSKLPSEIVGKSIDEEQIYDAVNVILSLQNTDGGF
ATYELTRSYPWLELMNPAETFGDIVIDYTYVECTSAAIQALVAFKKLYPGHRRDEIDNCV
AKAADFIESIQATDGSWYGSWGVCFTYGGWFGIRGLVAAGRRYDNCSSLRKACDFLLSKE
LASGGWGESYLSGQNKVYTNIKDDRPHIVNTGWAMLSLIDAGQSERDPTPLHRAARILIN
SQMDDGDF

>ASBAS1 AJ311789 BETA-AMYRIN SYNTHASE [AVENA STRIGOSA]
QADDGHWPGDYS
GILFIMPIIIFSLYVTRSLDTFLSPEHRHEICRYIYNQQNEDGGWGKMVLGPSTMFGSCM
NYATLMILGEKRNGDHKDALEKGRSWILSHGTATAIPQWGKIWLSIIGVYEWSGNNPIIP
ELWLVPHFLPIHPGRFWCFTRLIYMSMAYLYGKKFVGPISPTILALRQDLYSIPYCNINW
DKARDYCAKEDLHYPRSRAQDLISGCLTKIVEPILNWWPANKLR-DRALTNLMEHIHYDD
ESTKYVGICPINKALNMICCWVENPNSPEFQQHLPRFHDYLWMAEDGMKAQVYDGCHSWE
LAFIIHAYCSTDLTSEFIPTLKKAHEFMKNSQVLFNHPNHESYYRHRSKGSWTLSSVDNG
WSVSDCTAEAVKALLLLSKISADLVGDPIKQDRLYDAIDCILSFMNTDGTFSTYECKRTF
AWLEVLNPSESFRNIVVDYPSVECTSSVVDALILFKETNPRYRRAEIDKCIEEAVVFIEN
SQNKDGSWYGSWGICFAYGCMFAVRALVATGKTYDNCASIRKSCKFVLSKQQTTGGWGED
YLSSDNGEYIDSGRPNAVTTSWAMLALIYAGQVERDPVPLYNAARQLMNMQLETGDF

>CSOSC2 AB058508 MIXED-AMYRIN SYNTHASE [COSTUS SPECIOSUS]
QAHDGHWPGDCGA
GPMFLLPGLVISLHITGALNTILTPEHQKEMRRYLYNHQNVDGGWGLYSNGPSNMIGSVL
HYVTLRLLGEGANDGEGAMEKGRKWILDHGSATATSSWGKLWLSVLGVYEWAGNNPMPPE
FWLLPYCVPLHPGKMWCLSRTVYLPMSYLYGKRFVGAITPIVVSLRKELYNVPYDHIDWN
KARTDCAKEDQYYPHPLIQDIIWGSLHNFVEPILMRWPGSKLREKALSTVMQHIHYEDEN
TRYICVGPVNKALNMLCCWIDDPNSEAFKLHLPRVYDYLWLAEDGMKMKAYDGFQLWEAA
FAVQAIVSTNLSEEFGPTLKKAHEFVKNSQILEDCPGDLNYWYRHISKGAWTFSTADEGW
PVSDCTGEGLEAVLLLSMISPKIVGDPLDERRLYDAVNLLLSLMNKNGGFATYELTRSYA
WLEIMNPADVFINIVVDHQYVECTSSAIQPLALFKKLYPGHRQEEIDNCIMKAARFIERT
QRADGSWYGSWGVCFTYATWFGVKGLVAAGRTYENSCYIQKACNFLLSKQEASGGWGESF
LSCRKKVYINLEGNKTHAVNTSWAMLALIAAGQGERDPKPLHRAAKALINMQMENGDF

>OSCBPW AB055511 LUPEOL SYNTHASE [BETULA PLATYPHYLLA]
QAHDGHWPGESAGP
LFFLQPFVMALYITGDLNTIFSPAHQKEIIRYLYNHQNEDGGWGFHIEGHSTMFGSALSY
IALRILGEGLEDGEDGAMAKSRKWILDHGGLVAIPSWGKFWVTVLGLYEWSGCNPLPPEF
WFLPDIFPIHPGKMLCYCRLVYMPMSYLYGKRFVGPITGLIQSLRQELYNEPYHQINWNK
ARSTVAKEDLYYPHPLIQDLLWGFLHHVAEPVLTRWPFSMLREKALKAAIGHVHYEDENS
KYLCIGSVEKVLCLIACWAEDPNGEAYKLHLGRIPDNYWVAEDGLKIQSFGCQMWDAGFA
IQAILSCNLNEEYWPTLRKAHEFVKASQVPENPSGDFKAMYRHINKGAWTFSMQDHGWQV
SDCTAEGLKVAILFSQMPPDLVGEKIEKERLYDAVNVILSLQSSNGGFPAWEPQRAYGWL
EKFNPTEFFEDTLIEREYVECTSPAVHGLALFRKFYPRHRGTEIDSSIYRGIQYIEDVQE
PDGSWYGHWGICYTYGTWFAVGALAACGRNYKNCPALRKSCEFLLSKQLPNGGWGESYLS
SQNKVWTNIEGNRANLVQTAWALLSLIDARQAEIDPTPIHRGVRVLINSQMEDGDF

>GGLUS1 AB116228 LUPEOL SYNTHASE [GLYCYRRHIZA GLABRA]
QAHDGHWPAES
AGPLFFLQPLVMALYITGSLDDVLGPEHKKEIVRYLYNHQNEDGGWGFHIEGHSTMFGSA

LSYVALRILGEGPQDKAMAKGRKWILDHGGLVAIPSWGKFWVTVLGAYEWSGCNPLPPEL
WLLPKFAPFHPGKMLCYCRLVYMPMSYLYGKKFVGPITALIRSLREELYNEPYNQINWNT
ARNTVAKEDLYYPHPLIQDMLWGFLYHVGERFLNCWPFSMLRRKALEIAINHVHYEDENS
RYLCIGSVEKVLCLIARWVEDPNSEAYKLHLARIPDYFWLAEDGLKIQSFGCQMWDAAFA
IQAILACNVSEEYGPTLRKAHHFVKASQVRENPSGDFNAMYRHISKGAWTFSMHDHGWQV
SDCTAEGLKAALLLSEMPSELVGGKMETERFYDAVNVILSLQSSNGGFPAWEPQKAYRWL
EKFNPTEFFEDTMIEREYVECTGSAMQGLALFRKQFPQHRSKEIDRCIAKAIRYIENMQN
PDGSWYGCWGICYTYGTWFAVEGLTACGKNCHNSLSLRKACQFLLSKQLPNAGWGESYLS
SQNKVYTNLEGNRANLVQSSWALLSLTHAGQAEIDPTPIHRGMKLLINSQMEDGDF

>OEW AB025343 LUPEOL SYNTHASE [OLEA EUROPAEA]
QAHDGHWPAESAG
PLFFLPPLVLALYVTGAINVVLSREHQKEITRYIYNHQNEDGGWGIHIEGHSTMFGSVLS
YITLRLLGEGQEDGEDKAVARGRKWILDHGGAVGIPSWGKFWLTVLGVYEWDGCNPMPPE
FWLLPNFSPIHPGKMLCYCRLVYMPMSYLYGKRFVGPITGLVLSLRQEIYTEPYHGINWN
RARNTCAKEDLYYPHPLAQDMLWGFLHHFAEPVLTRWPFSKLREKALKVAMEHVHYEDMN
SRYLCIGCVEKVLCLIACWVEDPNSEAYKRHIARIPDYFWVAEDGLKMQSFGCQMWDAAF
AIQAILSSNLAEEYGPTLMKAHNFVKASQVQENPSGDFNEMYRHTSKGAWTFSMQDHGWQ
VSDCTAEGLKAALLFSQMPIELVGAEIETGHLYDAVNVILTLQSASGGFPAWEPQKAYRW
LEKLNPTEFFEDVLIERDYVECTSSAVQALKLFKQLHPGHRRKEIASCISKAIQYIEATQ
NPDGSWDGSWGICFTYGTWFAVEGLVACGKNYHNSPTLRRACEFLLSKQLPDGGWSESYL
SSSNKVYTNLEGNRSNLVQTSWALLSLIKAGQVEIDPGPIHRGIKLLVNSQMEDGDF

>TRW AB025345 LUPEOL SYNTHASE [TARAXACUM OFFICINALE]
QAHDGHWPAESA
GPLFFLPPLVIALYVTGAMNDILTPAHQLEIKRYIYNHQNEDGGWGLHIEGHSTIFGSVL
SYITLRLLGEEADSVAEDMALVKGRKWILDHGGAVGIPSWGKFWLTILGVYEWGGCNPMP
PEFWLMPKFFPIHPGKMLCYCRLVYMPMSYLYGKRFVGKITELVRDLRQELYTDPYDEIN
WNKARNTCAKEDLYYPHPFVQDMVWGVLHNVVEPVLTSRPISTLR-EKALKVAMDHVHYE
DKSSRYLCIGCVEKVLCLIATWVEDPNGDAYKRHLARIPDYFWVAEDGMKMQSFGCQMWD
AAFAIQAIFSSNLTEEYGPTLKKAHEFVKASQVRDNPPGDFSKMYRHTSKGAWTFSIQDH
GWQVSDCTAEGLKVSLLYSQMNPKLVGEKVETEHLYDAVNVILSLQSENGGFPAWEPQRA
YAWLEKFNPTEFFEDVLIEREYVECTSSAIQGLTLFKKLHPGHRTKEIEHCISRAVKYVE
DTQESDGSWYGCWGICYTYGTWFAVDALVACGKNYHNCPALQKACKFLLSKQLPDGGWGE
SYLSSSNKVYTNLEGNRSNLVHTSWALISLIKAGQAEIDPTPISNGVRLLINSQMEEGDF

>LCIMS1 AB058643 ISOMULTIFLORENOL SYNTHASE [LUFFA CYLINDRICA]
QASDGHWPSETS
GPLFYVCPLLICMYIMGFMDKVFSPEHKKEMMRYIYNHQNEDGGWGLHVGGHSNMFCTTF
NYISLRLLGEEPDVEAVCKARNWIHDHDGVTSILSWGKTWLSILNVFDWSASNPMPPEYW
MLPTWVPIHPSNMMCYTRITYMPMSYLYGKRFQAPLTPLVLQLRDELHTQPYDQINWRKV
RHMCATEDLYFPHPFVQDLLWDTLYLLSEPLMTRWPFNKLIRQKALNETMRHIHYEDENS
RYITIGCVEKPLCMLACWVEDPNSEYVKKHLARIPDYLWMAEDGMKMQSFGSQSWDAALA
MQALLSCNITREIGSVLNSGHDFIKNSQVRNNPPGDYKSMFRYMSKGSWTFSDCDHGWQV
SDCTAENLKCCLLLSLLPPDIVGEKMEPERFYDAVNVILNMQSKNGGLPAWEPASSYYWM
EWLNPVEFLEDLIIEHQHVECTSSALQAILLFRKQYPGHRRKEINNFINKAVQFLQDIQL
PDGSWYGNWGICYTYGTWFALKALSMAGKTYENCEAVRKGANFLRKIQNPEGGFGESYLS
CPYKRYIPLDGKRSNLVQTAWGMMGLICAGQADVDPTPIHRAAKLLINSQTEDGDF

>ATLAS1 NM114382 LANOSTEROL SYNTHASE [ARABIDOPSIS THALIANA]
QSQDGFWPGDYGG
PLFLLPALVIGLYVTEVLDGTLTAQHQIEIRRYLYNHQNKDGGWGLHVEGNSTMFCTVLS
YVALRLMGEELDGGDGAMESARSWIHHHGGATFIPSWGKFWLSVLGAYEWSGNNPLPPEL
WLLPYSLPFHPGRMWCHCRMVYLPMSYLYGRRFVCRTNGTILSLRRELYTIPYHHIDWDT
ARNQCAKEDLYYPHPKIQDVLWSCLNKFGEPLLERWPLNNLRNHALQTVMQHIHYEDQNS
HYICIGPVNKVLNMLCCWVESSNSEAFKSHLSRIKDYLWVAEDGMKMQGYNGSQLWDVTL
AVQAILATNLVDDYGLMLKKAHNYIKNTQIRKDTSGDPGLWYRHPCKGGWGFSTGDNPWP
VSDCTAEALKAALLLSQMPVNLVGEPMPEEHLVDAVNFILSLQNKNGGFASYELTRSYPE
LEVINPSETFGDIIIDYQYVECTSAAIQGLVLFTTLNSSYKRKEIVGSINKAVEFIEKTQ
LPDGSWYGSWGVCFTYATWFGIKGMLASGKTYESSLCIRKACGFLLSKQLCCGGWGESYL
SCQNKVYTNLPGNKSHIVNTSWALLALIEAGQASRDPMPLHRGAKSLINSQMEDGDY

>OSC7 AB244671 LANOSTEROL SYNTHASE [LOTUS JAPONICUS]
QAQDGFWPGDYA
GAMFMLPGLVIGLSVTGALNAALSPEHQSEMKRYVLNHQNEDGGWGLHIEGPTTMFGTVL
NYVAMRLLGEDIDGGDGAMKKARKWILDRGGATSIPSWGKFWLSVLGVYEWRGINPMPPE
LWLLPYSLPSHPGRMWCHTRLVYLSMSYLYGRRFVGPFNALVLSLRKELYTLPYHLLDWN
EARNLCAKEDLSHPRPGIQNILWGLLHHVGEPLLTHKLFSRLRQKALHHVMEHIHNEDEA
SNYICIGPVNKVLNMICCWLEDPNSQAFKYHISRIKDYLWVAEDGMKMQGYGGSQLWDVA
FSVQAILATNLDDEYGSMLKRANEFIKCSQITTNSSSNPSAWYRHISKGSWGFSTPDNGW
PVSDCTAEGLKAAILLSNFPSETVGKAMETEKLYDAVSWVLSMQNENGGFASYELTRSYA
WLEKINPVETFRDIMIDYQYVECTSAAIQGLALFTQRYPEYRRREIDSCIAKAARYIEST
QLADGSWYGSWGICYTYATWFGIKGLIAASKSYQESKSIRRACEFLLSKQLLSGGWGESY
LSCELKVYTNLEGNKSHLVNTAWAMLALIEGGQAERDPTPLHRAAKVLINSQMENGEF

>CPQ AB116238 CUCURBITA PEPO
QTRDGN
WASDLGGPLFLLPGLVIALHVTGVLNSVLSKHHRVEMCRYLYNHQNEDGGWGLHIEGTST
MFGSALNYVALRLLGEDADGGDGGAMTKARAWILERGGATAITSWGKLWLSVLGVYEWSG
NNPLPPEFWLLPYSLPFHPGRMWCHCRMVYLPMSYLYGKRFVGPITPKVLSLRQELYTIP
YHEIDWNKSRNTCAKEDLYYPHPKMQDILWGSIYHVYEPLFTRWPGKRLREKALQAAMKH
IHYEDENSRYICLGPVNKVLNMLCCWVEDPYSDAFKLHLQRVHDYLWVAEDGMRMQGYNG
SQLWDTAFSIQAIVATKLVDSYAPTLRKAHDFVKDSQIQEDCPGDP

>CABAS AY520818 CENTELLA ASIATICA PUTATIVE BETA-AMYRIN SYNTHASE (OSCCAS)
QAHDGHWPAE
NAGPMFFTPPLIIALYISGAIDTHLTIQHKKEMIRFIYLHQNKDGGWGFYIEGHSTMIGS
ALSYVALRLLGEGPDDGDGAVERARKWILDHGGAASIPSWGKTYLAVLGVYEWEGCNPLP
PEFWLFPEALPYHPAKMWCYCRTTYMPMSYLYGKKYHGPITDLVISLRKEIHPIPYEKIN
WNKQRHNCNKEDLYYPHSFIQDLLWDGLHYFTEPIIKMWPFNKLRKKGMKRAIELMRYGG
YESRFITIGCVSKSLDMMCWWAENPNGPEFKHHLARVPDYLWLAEDGMKMQSFGSQLWDC
VLATQAVMSTGMVDEYGDCLKKAHFYIKESQCKKNPSGDY

>RSM1 AB263203 MULTIFUNCTIONAL TRITERPENE SYNTHASE [RHIZOPHORA STYLOSA]
QASDGHWPAENA
GPLFFLPPLVMCMCITGHLDTVFPAEHRKEILRYIYYHQNEDGGWGLHIEGHSTMFCTAL
NYICMRILGEGPNGGQDDACTRARKWIHDHGSVTNIPSWGKTWLSILGVYDWSGCNPMPP
EFWMLPSFLPMHPAKMWCYCRMVYMPMSYLYGKRFVGLITPLIQQLREELFTQPYDQINW
KKNCHQCAPEDLYYPHPFIQDLIWDCLYISMEPLLTRWPLNMIIRKKALELTMKHIHYED
GSSRYITIGCVEKVLCMLACWVEDPNGDYFKKHLARIPDYIWVAEDGMKMQSFGSQQWDT
GFAIQALLATNLTDEIGGVLRRGHDFIKKSQVQDNPSGDFKSMYRHISKGSWTFSDQDHG
WQVSDCTAEGLKCCLLFSMMPPEIVGEHMEPERLYDSVNVLLSLQSKNGGLSAWEPAGAQ
DWLELLNPTEFFADIVIEHEYVERTSSAIHALVLFKKLYPGHRKKEIEDFIAKSVRFLES
IQTSDGTWYGNWGVCFTYGTWFALGGLAAAGKTYNSCLAMRKAVDFLLRIQKDDGGWGES
YLSCPEKKYVPLEANHSNLVHTAWAMMALVHAGQMDRDPTPLHRAAKLMINSQLEDGDF

>RSM2 AB263204 MULTIFUNCTIONAL TRITERPENE SYNTHASE [RHIZOPHORA STYLOSA]
QSSDGFWPAD
ASAPVFYLAPWVIGLYVIGHLNTVFPAEHQKEILRYIYCHQNEDGGWGLHYEDGGTMFGT
AFNYVCMRILGEGPGGGRDNACERARKGILDHGGVTYIPSGGKTWLAMLGVFDWSGCNPM
PPEFWMLPPFFPMHPAQMWCYCRIVYMPMSYLYGRRFVGPITPLVQQLREELHTQPFHEI
EWSKARHLCAKEDLFHRRPWIQELFWDCLHTFAEPLLTRWPLNNFIREKALKITMEHVHY
DDKASHYINPGSVEKVICMVACWVEDPSGEPFQRHLARISDYVWIAEDGMRITGIGSQTW
DAALSIQALIACNLIEEMGPTLKKGYDFLKNSQAKDNPPGDFKRMYRHFGKGAWAFSSQD
YGVIALDCTAESLMCCLHFSMMPPEIVGEKLEPEKLYLAVDFILSLQSKNGGLTCWEPAR
GGKWLEVLNPLEFFENIVVEHEYVEVTASAINALVMFKKRYPGYREKEIEHFISKAVHYL
IQTQFPNGPWYGVWGICFMYGTYFALKGLAAAGNTYANCPAIPKAVDFLLKTQCQDGGWG
ESYLSGTTKVYTPLEGNRSNLVQTAWALMGLIHSGQAERDPTPLHRSAKLLINSQTSDGD
F
>BGBAS AB289585 BETA AMYRIN SYNTHASE [BRUGUIERA GYMNORHIZA]
QASDGHWPAEIA
GPLFFLPPLVMCVYITGHLDAVFPAEHRKEILRYIYYHQNEDGGWGLHIEGHSTMFCTAL

NYICMRIIGEGPNGGQDDACARARKWIHDHGSVTNIPSWGKTWLSILGVYDWSGSNPMPP
EFWMLPSFLPMHPAKMWCYCRMVYMPMSYLYGKRFVGPITPLIQQLREELFTQPYDQINW
KKTRHQCAPEDLYYPHPFVQDLIWDCLYIFTEPLLTRWPLNEIIRKKALEVTMKHIHYED
ESSRYITIGCVEKVLCMLACWVEDPNGDYFKKHLARIPDYIWVAEDGMKMQSFGSQEWDT
GFAIQALLATNLTDEIGDVLRRGHDFIKKSQVRDNPSGDFKSMYRHISKGSWTFSDQDHG
WQVSDCTAEGLKCCLLFSMMPPEIVGEHMVPERLYDSVNVLLSLQSKNGGLSAWEPAGAQ
EWLELLNPTEFFADIVIEHEYVECTSSAIHALVLFKKLYPGHRKKEIDNFIVNAVRYLES
IQTSDGGWYGNWGVCFTYGTWFALGGLAAAGKTYNNCLAMRKAVDFLLRIQRDNGGWGES
YLSCPEKRYVPLEGNRSNLVHTAWALMALIHAGQMDRDPTPLHRAARLMINSQLEDGDF

>BGLUS AB289586 LUPEOL SYNTHASE [BRUGUIERA GYMNORHIZA]
QASDGHWCAENS
GPMFFVPPMVFSLYITGHLNAVFSAEHCKEILRYIYCHPNEDGGWGLHIEGHSAMFSTVL
NYNWLGKLGEGRDGGKDNACERARRRILDHGSATAISSWGKTWLAILGVYEWDGCNPMPP
EFWAFPTFFPIHPARMLCYCRLTYMAMSYLYGKKFVGPITPLILQLREEIYNEPYDQINW
SRMRHLCAKEDNYYAHTLTQIILWDAIYMLGEPLLKRWPFNKLREKALKITMDHIHYEDE
NSQYITIGSVEKPLLMLACWHEDPNGDAFKKHLARIPDYVWLGEDGIKIQSFGSQVWDTS
FVLQALIASNLPSETGPTLEKGHNFIKNSQVTQNPSGDFRRMFRHISKGSWTFSDKDHGW
QVSDCTAESLKCCLLFSMMPPELVGEKMGPQRMYDAVNVIISLQSKNGGCSAWEPAGAGS
WMEWLNPVEFLADLVIEHEYVECTSSSLQALVLFKKLYPEHRRKEIEIFILNAVRFTEEI
QQPDGSWYGNWGICFLSGTWFGLKGLAAAGKTYYNCTAVRKGVEFLLQTQRDDGGWGESY
LSCPKKIYVPLEGNRSNLVQTALAMMGLILGGQGERDPTPLHRAAKLLINSQTELGDF

>OSC3 AB181245 LUPEOL SYNTHASE [LOTUS JAPONICUS]
QAHDGHWPAESAGP
LFFVQPLVMALYITGSLDDVLGPQHKKEIIRYLYNHQNEDGGWGFHIEGHSTMFGSALSY
IALRVLGQSLEDGEDMAVARGRKWILDHGGLVAIPSWGKFWVTVLGVYEWSGCNPLPPEF
WLLPKIFPIHPGKMLCYCRLVYMPMSYLYGKKFVGPITALVRSLRKELYNEPYDRVDWNK
ARNTVAKEDLYYPHPLIQDMLWGFLHHVGERVLNTWPFSMLRQKAIEVAINHVRYEDETT
RYLCIGSVEKVLYLIARWVEDPNSEAYKLHLARIPDYFWLAEDGLKIQSFGCQMWDAAFA
IQAILSGNVSEEYGPTLKKAHHFVKASQVRENPSGDFKAMYRHISKGAWTFSMHDHGWQV
SDCTAEGLKVALLLSEMSDDLVGAKMETEQFYDAVNVILSLQSSNGGFPAWEPQRAYQWL
EKFNPTEFFEETLIEREYVECTGSAMQALALFRKLYPKHRRKEIDRCISKAIRYIENTQN
PDGSWYGCWGICYTYGTWFAVEGLTACGKNFQNSVTLRRACKFLLSKQLPNGGWGESYLS
SQDKVYTNIEGKRANLVQSSWALLSLMRAGQAEIDPTPIHRGIRLLINSQMDDGDF

>AT1G78950 (ATBAS)
QASDGHWPAEN
AGPLFFLPPLVFCLYITGHLDEVFTSEHRKEILRYIYCHQKEDGGWGLHIEGHSTMFCTT
LNYICMRILGESPDGGHDNACGRAREWILSHGGVTYIPSWGKTWLSILGVFDWSGSNPMP
PEFWILPSFFPVHPAKMWSYCRMVYLPMSYLYGKRFVGPITSLILQLRKELYLQPYEEIN
WMKVRHLCAKEDTYYPRPLVQELVWDSLYIFAEPFLARWPFNKLLREKALQLAMKHIHYE
DENSRYITIGCVEKVLCMLACWVEDPNGDYFKKHLSRISDYLWMAEDGMKMQSFGSQLWD
TGFAMQALLASNLSSEISDVLRRGHEFIKNSQVGENPSGDYKSMYRHISKGAWTFSDRDH
GWQVSDCTAHGLKCCLLFSMLAPDIVGPKQDPERLHDSVNILLSLQSKNGGMTAWEPAGA
PKWLELLNPTEMFSDIVIEHEYSECTSSAIQALSLFKQLYPDHRTTEITAFIKKAAEYLE
NMQTRDGSWYGNWGICFTYGTWFALAGLAAAGKTFNDCEAIRKGVQFLLAAQKDNGGWGE
SYLSCSKKIYIAQVGEISNVVQTAWALMGLIHSGQAERDPIPLHRAAKLIINSQLESGDF

>AT1G78955 (CAMS1)
QASDGHWPAEN
AGPLFFLPPLVFCLYVTGHLHEIFTQDHRREVLRYIYCHQNEDGGWGLHIEGNSTMFCTT
LNYICMRILGEGPNGGPGNACKRARDWILDHGGATYIPSWGKTWLSILGVFDWSGSNPMP
PEFWILPSFLPIHPAKMWCYCRLVYMPMSYLYGKRFVGPISPLILQLREEIYLQPYAKIN
WNRARHLCAKEDAYCPHPQIQDVIWNCLYIFTEPFLACWPFNKLLREKALGVAMKHIHYE
DENSRYITIGCVEKALCMLACWVEDPNGIHFKKHLLRISDYLWIAEDGMKMQSFGSQLWD
SGFALQALVASNLVNEIPDVLRRGYDFLKNSQVRENPSGDFTNMYRHISKGSWTFSDRDH
GWQASDCTAESFKCCLLLSMIPPDIVGPKMDPEQLYEAVTILLSLQSKNGGVTAWEPARG
QEWLELLNPTEVFADIVVEHEYNECTSSAIQALILFKQLYPNHRTEEINTSIKKAVQYIE
SIQMLDGSWYGSWGVCFTYSTWFGLGGLAAAGKTYNNCLAMRKGVHFLLTTQKDNGGWGE
SYLSCPKKRYIPSEGERSNLVQTSWAMMGLLHAGQAERDPSPLHRAAKLLINSQLENGDF

>XP_021819928.1
QSPHGHWPAENAGPNFYFPPLVMAAYIPGYLN
VIFTPEHKKEILRYTYNHQNEDGGWGLHIAGPSMMFTTCLNYCMMRILGDGPDGGRDNACARARKWILDR
GGACYAASWGKTWMAILGVYDWEGSNPMPPEFWTYPTLLPFHPSKMFCYCRLTYLPMSYFYATRFVGPIT
PLVEELRQEIYCEPYSEINWSTVRHSCAKEDNYYPHGRVQRFMWDSLYTVVEPLLKRWPFKKIRDNAIQF
TIDQIHYEDENSRYITIGCVEKPLMMLACWAEDPSGEAFKKHIPRVTDYIWLGEDGIKMQSFGSQSWDCA
LVIQALLAGNLNAEMGPVLKKAHEFLKISQVRINTSGDYLAHFRHISKGAWTFSDRDHGWQVSDCTAEAL
RCCCLFANMSPEVVGEPMEAECMYDAVNVIMSLQSPNGGVSAWEPTGAPKWLEWLNPVEFLEDLVIEYEY
IECTSSSIQALILFRKLYPGHRRKEINNFITRAADYIEDIQYPDGSWYGNWGICFVYGTWFAIKGLEAAG
RTYNNCEAVRKGVDFLLKTQREDGGWGEHYTSCTNKKYTAQDSTNLVQTALGLMGLIHGRQAERDPTPIH
RAARALMNGQLDDGDF

>XP_021810674.1
MWDASFAMQALLAANLNDELGSVLKKGHDFLKKSQVRDNPSGDFVAHFRHISKGGWTFSDQDHGWQVSDC
TAEALKNCLLLSMLPPQLVGEQLEPERLYDAVNVVLSLQGPNGGVSAWEPAGAPKWLEWLNPIEFMGDLV
IEYEHVECTSSSIQALALFRKLYPTHRRKQIDNFITTAAGFIEDIQSPDGSWYGNWGICFMYGTWFAIRG
LEAAGKTYNNCEAIRRGVEFLLKTQRDDGGWGESYISCTNKIYTPLEGDRSNVVQTAMGLMGLIHGGQAE
RDPTPIHQAAKMLINSQLENGDF

>XP_021819927.1
QSPHGHWPAQNTGPLFYTPPFVIALYITG
HLNMVFSADHRKEMLWFMYCHQNEDGGWGLHIEEGQSMMLCTLLNYICMRLLGEGPDGGLDNACARARKW
ILDHGGATHSSSWGKIWMAMLGVYDWEGNNPIPPELWICPSFLPFHPAKILCYTRMSYLAMSYLFGKRVV
GPITPLILQLREEIYNEPYNEIKWSEVRHLCAKEDKYHPHRKLQCLMWDSLHTICEPLFTRWPFKKLREK
ALQETSKHIHYEDENTRYITIGVGGKPLNMLACWAEDPNGESYKKHLARIADYIWVAEDGMTMQSFGSQM
WDASFAIQALLAANLNDELGSVLKKGHDFIKKSQVRDNPSGDFLAYFRHISKGAWTFSDQDQGLQVSDCT
AEGLKCCLLLSMLPPQLVGEQLEPERLYDAVNVILSLQSPNGGVSAWEPAGAPKWLEWLNPVEFLDHLII
EYEHVECTSSSIQALALFRKLYPAHRKKQIDNFITTAAGFIEDIQSPDGSWYGNWGICFIYGTWFAISGL
EAAGKTYNNCEAIRRGVEFLLKTQRDDGGWGESYISCETKIYTPLEGDRSNLVQTAMGLMGLIHGGQAER
DPTPIHQAAKMLINSQLENGDF

>XP_021819911.1
QASDGHWPAENAGPLFFLPPLVMCAYITGHLN
TVFPAEHRKEIMRYIYYHQNEDGGWGLHIEGHSTMFCTALSYICMRILGEGPDGGQDNACARARKWILDH
GSVTHIPSWGKTWLSILGVFEWSGSNPMPPEFWVLPSFLPMHPAKMWCYCRMVYMPMSYLYGKRFVGPIT
PLILQLREELYAQPYNEINWKGVRHHCAKEDIYYPHPWIQDIMWDSLYICTEPLLNRWPFNKLIREKALQ
VTMKHIHYEDENSRYITIGCVEKVLCMLACWAEDPNGDYFKKHLARIPDYLWVAEDGMKMQSFGSQEWDT
GFAIQALLASNLTDEIGPTLARGHDFIKKSQVKDNPSGDFKSMYRHISKGSWTFSDQDHGWQVSDCTAEG
LKCCLLFSVMRPDIVGEKMEPERLYDSINVLLSLQSKNGGLAAWEPAGAADWLEMLNPTEFFEDIVVEHE
YVECTSSAIQALVLFKKLYPGHRKREIDHFITNATQYLESIQMPDGSWYGNWGVCFTYGSWFALGGLAAA
GKTFNNCLAMRKGVNFLLKTQRENGGWGESYLSCPKKEYVPLEGNRSNLVHTAWAMMGLIHAGQAHRDPA
PLHRAVKLIINSQMENGDF

>XP_021819916.1
MFCTALSYICMRILGEGPDGGQDNACARARKWILDHGSVTHMPSWGKTWLSILGVFEWSGSNPMPPEFWM
LPSFLPMHPAKMWCYCRMVYMPMSYLYGKRFVGPITPLILQLREELYAQPYDEINWKGVRHLCAKEDIYY
PHPWIQDIMWDSLYICTEPLLTRWPFNKLVREKALQVTMKHIHYEDENSRYITIGCVEKVLCMLACWAED
PNGDYFKKHLARIPDYLWVAEDGMKMQSFGSQQWDTGFAIQALLASNLADEIGPTLARGHDFIKKSQVKD
NPSGDFRSMHRHISKGSWTFSDQDHGWQVSDCTAEGLKCCLLFSTMRPDIVGEKMEPQRLYDSVNVLLSL
QSKNGGLAAWEPAGAAEWLEMLNPTEFFADIVVEHEYVECTSSAIQALVLFKKLYPGHRKKEIDHFITNA
TEYLENIQMPDGSWYGNWGVCFTYGSWFALGGLAAAGKTFNNCLTVRKGVNFLLTTQRENGGWGESYLSC
PKKEYVPLEENRSNLVHTAWAMMGLIQAGQAERDPAPLHRAAKLIINSQMENGDF

CYP protein sequences used for phylogenetic analysis

>XP_021823674.1 P. avium
AYWEGKDEVEVYPLTKTLTLGLACRFFLGIDEPDRIARFVSNF
DDVTVGMHSLILNFPGTTFYKATKAADELRRELKIVIQEKKTAMASGAPMHDILSHMILASDPTGKHMPE
AEIADKIMGLLTAGYSTVATAMTFFMKYVGERPDIYAKVLAEHKEIAESKKPGQFLEWDDINKMKYSWNA
VNEVMRFTPPLQGTFREALTDFTYAGYTIPKGWKVYWTVSTTNMNPQYFPNPEKFDPSRYDDLNAFPAFT
FVPFGGGPRMCPGKEYARLAILTFVHNVVMRFKWEVLFPNEKITGDM

>XP_021816761.1 P. avium
DGWENRKEVEVFPLAKNYTFWLAARLFVSLEDATEIARLGDPFALLAS
GIISMPLDFPGTPFYKAIKASNFIREELTKIIKQRKVDLAEGKASPTQDILSHMLLLCDEHGSHMKEHDI
ADKILGLLIGGHDTASATCTFIVKYLAELPHIYDEVYKEQMEILSCKAPGELLNWDDLQKMKYSWNVAQE
VLRLAPPLQGAFREALADFVFNGFTIPKGWKLYWSANSTHKNADYFPEPFKFDPTRFEGNGPAPYTFVPF
GGGPRMCPGKEYARLEILVFMHNLVKRFKWEKILPDEKIVVDP

>XP_021815663.1 P. avium
LNWHGKQKITVLPLMKNLTFNIICSLLFGVERGPRRDELIECFQLMIEGVWSVPL
NLPFTRFNCSIKASKRVQNMLKELICEKRMQLEQKTALPLQDLITCLLSIRNVDNEEELTEKEILHNIII
VMVAGYDTSSVVITFLLRLLANEPAVYAALLQEQEEIARSKSFGELLTWEDLAKMKYTWRVTMEILRTTP
PVFGGMRRAMKDIEYGGFLIPQGWQIFWAIPMTHNDDSIFPEPSKFDPSRFENQKSVPPYSFVPFGGGTR
ICPGYEFARIEILVAIHYMVTQFTWKLCADNKFSRVP

>CYP705A1 A. thaliana NP_193268.3
DKAMKKESVMIHKEASRFVNNSLYKMCTGRSFSVENNEVERIM
ELTADLGALSQKFFVSKMFRKLLEKLGISLFKTEIMVVSRRFSELVERILIEYEEKMDGHQGTQFMDALL
AAYRDENTEYKITRSHIKSLLTEFFIGAADASSIAIQWAMADIINNREILEKLREEIDSVVGKTRLVQET
DLPNLPYLQAVVKEGLRLHPPTPLVVREFQEGCEIGGFFVPKNTTLIVNSYAMMRDPDSWQDPDEFKPER
FLASLSREEDKKEKILNFLPFGSGRRMCPGSNLGYIFVGTAIGMMVQCFDWEINGDKINM

>CYP705A5 A. lyrata EFH40098.1
DKAMKKECVEIRNEAMKLTNNTICKMIMGRSCSEENGEA
ETVRGLVTESIFLTKKHFLGAMFHKPLKKLGISLFAKELMSVSNKFDELLEKILVKHEEKLQEHHQCTDM
LDMLLEAYGDEKAEYKITRDQIKSLFVDLFSAGTESSANTIQWTMAEIINNPKISERLREEIDSVVGNIR
LVQETDLPNLPYLQAIVKEGLRLHPPGPVVRTFQETCEIKGFYIPEKTRLFVNIYAIMRDPDFWEDPEEF
KPERFLTSSRLGQEDEKREDMLKYIPFGSGRRACPGSHLAYAVVGSVIGVMVQNFDWRIKGEKINM

>CYP93E1 G. max NP_001236154.2
ISGNGNYEVVMRKELITHTNNIITRMIMGKKSNAENDEVARLRKVVR
EVGELLGAFNLGDVIGFMRPLDLQGFGKKNMETHHKVDAMMEKVLREHEEARAKEDADSDRKKDLFDILL
NLIEADGADNKLTRESAKAFALDMFIAGTNGPASVLEWSLAELVRNPHVFKKAREEIESVVGKERLVKES
DIPNLPYLQAVLKETLRLHPPTPIFAREAMRTCQVEGYDIPENSTILISTWAIGRDPNYWDDALEYKPER
FLFSDDPGKSKIDVRGQYYQLLPFGSGRRSCPGASLALLVMQATLASLIQCFDWIVNDGKNHHV

>CYP93E2 M. truncatula ABC59085.1
ISKNGKPIEMRHELIRHTNNIISRMTMGKKSSGMNDEVGQLRKVVRE
IGELLGAFNLGDIIGFMRPLDLQGFGKRNKDTHHKMDVMMEKVLKEHEEARAKEGAGSDRKKDLFDILLN
LIEADDGAESKLTRQSAKAFALDMFIAGTNGPASVLEWALAELIRNPHVFKKAREEIDSTVGKERLFKES
DIPNLPYLQAVVKETLRMHPPTPIFAREATRSCQVDGYDVPAFSKIFINAWAIGRDPNYWDNPLVFNPER
FLQSDDPSKSKIDVRGQYYQLLPFGSGRRSCPGSSLALLVIQATLASLIQCFDWVVNDGKSHDI

>CYP93E3 G. uralensis BAG68930.1
ISKTGKGVEMRQELIRHTNNIISRMTMGKKSNGTNDEVGQVRKLVRE
IGELLGAFNLGDIIGFMRPFDLQGFGKKNRDAHHNMDVMMEKVLKEHEEARAKEKGGAESDRKKDLFDIL
LNLIEADGADNKLTRESAKAFALDMFIAGTNGPASVLEWSLAELIRNPQVFKKAREEIDSVVGKERLVKE
SDIPNLPYLQAVVKETLRMHPPTPIFAREAIRGCQVDGYDIPANSKIFINAWAIGRDPKYWDNPQVYSPE
RFLITDEPGKSKIDVRGQYYQLLPFGSGRRSCPGSSLALLVIQATLASLVQCFDWVVNDGKNSEI

>CYP71A16 A. thaliana NP_199073.1
EASLSSSSVNLSKLITNMVSDIMGKVVLGKKYSGEEGTIDVKTITKSFLD
AVGLSPVGEYIPSLAWIGKITGSDGKLEKITKQFGDFIEKVLQEHEDTTADKETPDFVDMLLTIQRDETA

QCQLDKSDLKVIIFEMFLGSTTTTSAVIEWAMTRLMRNPECLKKLQDEIRSVSKMNSYVSGKEVENMNYL
KAVIKEVLRLHPPLPLLVPRLLSEDVKLKGYDITAGTQVIINAWAIQRDTATWGSDAQEFRPERHFDSTW
DFVGRNFKYIPFGAGRRLCPGIGLGSVMASVTLANLVKRFDWRVEDGPSGYD

>AHB62239.1 L. japonicus
ISMNTGSCVNLTDIVLSMTYSIVARAAFGDKCKDQEAYILFMKKSMRV
AESFSVTNLFPSQRWLLVISGAMNKFKELHRTTDKVLEKIITEATAKSGGDGSLLSILLNLKDHGDPEFH
LTINNIKAVLQDMFIAGSETSSTSLEWTFSEMLKNPRVMKRAQAEVRQVFGSRGYVEEMALEELKFLKAV
IKETLRLHPPIPLFPRECGETCEIDGYTIPVGTQVIVNTWAIGRDLCWSEEEKFYPERFLDCPIDYKGSN
FEFIPFGAGKRICPGILFALPNIVLPLAQLLYYFDWELPFGTSHED

>CYP71D313 AEY75217.1 P. ginseng
IKTSLDSPVNLTHKFTSLTNAITCRAAIGERSKYQDELVHLIELMAALGGGF
DIADLFPSYKFLHFLSGLRSKLEKVRKRLDDIFYNILKEHEEKRAKTKNSDGRVAGEEDLVDVLLRVQEK
GGLQFPISSNNIQGIICDMLTAGTDTASTALDWAMSELVRYPSVLHKAQAEVREAFKGKTKIHEDDVQGL
SYLKLVIKETLRLHPPAPLLLPKECREQCVIEGYTIPVRTKLIVNAWAIGRDPEYWVNAESFDPERFSNK
SIDYNGTNLNYIPFGAGRRSCPGIAFGIATIELPLALLLYHFNWGMPGGIKPSA

>CYP51H10 ABG88965.1 A. strigosa
AKWGDEGEVDLKQEFTKLLMLIAGRCLLGSEVRDTIFGEFYTLFADIEE
GVNLFSYMFPYMPVPVNNRRDRAQMKLTSIVSEIVRSRKRCNRVEDDMLQRLIDSRYKDGRPTTEGEVSG
MIIGLIFAGKHTSTITASWTGACLLTHPKFLGAAVEEQKQMMSKYKDNIDYNILSEMEILHSCIKEAGRM
YPAAPVLLRKTLKEISVQTREGGEYGIPKGTTLAHLVMLTGKVPHTYKDPEVYDPDRFRVGREEDKIGGK
LSYTIFGAGRHACAGESFAFMQIKIIWSHLLRNFDLKLTSPFPKQD

>CYP72A68v2 XP_013463952.1 M. truncatula
LSSNGSCEMDVWPSLQSLTSDVIA
RSSFGSSYEEGRKVFQLQIEQGELIMKNLMKSLIPLWRFLPTADHRKINENEKQIETTLKNIINKREKAI
KAGEATENDLLGLLLESNHREIKEHGNVKNMGLSLEEVVGECRLFHVAGQETTSDLLVWTMVLLSRYPDW
QERARKEVLEIFGNEKPDFDGLNKLKIMAMILYEVLRLYPPVTGVARKVENDIKLGDLTLYAGMEVYMPI
VLIHHDCELWGDDAKIFNPERFSGGISKATNGRFSYFPFGAGPRICIGQNFSLLEAKMAMALILKNFSFE
LSQTYAHAP

>CYP72A61v2 XP_003605470.1 cytochrome P450 72A15 M. truncatula
SSSNGPCELDIW
PFVQNVSSDVLARAGFGSSFEEGKRVFQLQKEMISLTMTLFKFAFIPGYRFLPTYTNRRMKAIDLEIRTS
LMKIINRRLKAIKAGEPTNNDLLGILLESNYKESEKGNGGGGMSLRDVVDEVKLFYLAGQEANAELLVWT
LLLLAKNPEWQAKAREESFQVFGNENPDFEKIGQLKIVSMILQESLRLYPPVIMLSRFLRKDTKLGDLTL
PAGVELIVPVSMMHQEKEFWGDDAGDFKPERFSEGVSKATNGKVSYLPFGWGPRLCIGQNFGLLEAKIAV
SMILRQFSLEFSPSYTHAP

>CYP72A63 XP_024628552.1 M. truncatula
LSSDGTCEIDVWPSLQN
FTCDVISRTAFGSSYAEGTKLFQLLKKQGFLLMTGRHTNNPLWGLLATTTKTKMKEIDREIHDSLEGIIE
KREKALKNGETTNDDLLGILLQSNHAEKQGHGNSKNIGMTTQDVIDECKLFYLAGQETTXSLLVWTMVLL
GRYPEWQARAREEVLQVFGNQNPNNEGLSQLKIVTMILYEVLRLFPPLIYFNRALRKDLKLGNLLLPEGT
QISLPILLIHQDHDLWGDDAKEFKPERFAEGIAKATKGQVSYFPFGWGPRICLGQNFALLEAKIAVSLLL
QNFSFELSPNYVHVP

>CYP72A154 BAL45207.1 G. uralensis
LSSDGTCEVDVWPFLQNL
TCDVISRTAFGSSYAEGAKIFELLKRQGYALMTARYARIPLWWLLPSTTKRRMKEIERGIRDSLEGIIRK
REKALKSGKSTDDDLLGILLQSNHIENKGDENSKSAGMTTQEVMEECKLFYLAGQETTAALLAWTMVLLG
KHPEWQARARQEVLQVFGNQNPNFEGLGRLKIVTMILYEVLRLYPPGIYLTRALRKDLKLGNLLLPAGVQ
VSVPILLIHHDEGIWGNDAKEFNPERFAEGIAKATKGQVCYFPFGWGPRICVGQNFALLEAKIVLSLLLQ
NFSFELSPTYAHVP

>CYP88D6 B5BSX1.1 G. uralensis
ELSSMKHPVELLKEMKKVSFKAIVHVFMGSSNQDIIKKI
GSSFTDLYNGMFSIPINVPGFTFHKALEARKKLAKIVQPVVDERRLMIENGPQEGSQRKDLIDILLEVKD
ENGRKLEDEDISDLLIGLLFAGHESTATSLMWSITYLTQHPHILKKAKEEQEEITRTRFSSQKQLSLKEI

KQMVYLSQVIDETLRCANIAFATFREATADVNINGYIIPKGWRVLIWARAIHMDSEYYPNPEEFNPSRWD
DYNAKAGTFLPFGAGSRLCPGADLAKLEISIFLHYFLRNYRLERINPECHVTSLP

>CYP708A2 AED95608.1 A. thaliana
GSFDAKEAVESVIMAHLTPKIISNLKPETQATLVDNIMALGSE
WFQSPLKLTTLISIYKVFIARRYALQVIKDVFTRRKASREMCGDFLDTMVEEGEKEDVIFNEESAINLIF
AILVVAKESTSSVTSLAIKFLAENHKALAELKREHAAILQNRNGKGAGVSWEEYRHQMTFTNMVINETLR
MANMAPIMYRKAVNDVEIKGYTIPAGWIVAVIPPAVHFNDAIYENPLEFNPWRWEGKELRSGSKTFMVFG
GGVRQCVGAEFARLQISIFIHHLVTTYDFSLAQESEFIRAP

>CYP716A53v2 AFO63031.1 P. ginseng
DKWNGSTEVKAFAMSESLTFELACWLLFSINDPVQVQKLSHLFEKVKAGLLSLPL
NFPGTAFNRGIKAANLIRKELSVVIKQRRSDKLQTRKDLLSHVMLSNGEGEKFFSEMDIADVVLNLLIAS
HDTTSSAMGSVVYFLADHPHIYAKVLTEQMEIAKSKGAEELLSWEDIKRMKYSRNVINEAMRLVPPSQGG
FKVVTSKFSYANFIIPKGWKIFWSVYSTHKDPKYFKNPEEFDPSRFEGDGPMPFTFIPFGGGPRMCPGSE
FARLEVLIFMHHLVTNFKWEKVFPNEKIIYTP

>CYP716A1 AED94045.1 A. thaliana
TEWANQDQVIVFPLTKKFTFSIACRSFLSMEDPARVRQLEEQFNTVA
VGIFSIPIDLPGTRFNRAIKASRLLRKEVSAIVRQRKEELKAGKALEEHDILSHMLMNIGETKDEDLADK
IIGLLIGGHDTASIVCTFVVNYLAEFPHVYQRVLQEQKEILKEKKEKEGLRWEDIEKMRYSWNVACEVMR
IVPPLSGTFREAIDHFSFKGFYIPKGWKLYWSATATHMNPDYFPEPERFEPNRFEGSGPKPYTYVPFGGG
PRMCPGKEYARLEILIFMHNLVNRFKWEKVFPNENKIVVDP

>CYP716A2 AED94048.1 A. thaliana
TEWANQDQLIVFPLTKKFTFSIACRLFLSMDDLERVRKLEEPFTTVMTGVF
SIPIDLPGTRFNRAIKASRLLSKEVSTIIRQRKEELKAGKVSVEQDILSHMLMNIGETKDEDLADKIIAL
LIGGHDTTSIVCTFVVNYLAEFPHIYQRVLEGMQIPLL
>CYP716A20 XP_002264643.4 V. vinifera
NHWDLNETVTVFPLAKQYTFMVACRLFLSIDDPKHIAKFANPFHILAAGVMSIPINFPGTPFNRAI
KAADSVRKELRAIIKQRKIQVLAGKSSSSKHDILSHMLTTTDENGQFLNEMDIADKILGLLIGGHDTASA
VITFIIKYLAELPQVYNEVLKEQMEVAAGKKSGEMLDWEDIQKMKYSWNVANEVMRLAPPLQGSFREAIT
DFTYAGFSIPKGWKLYWSTNATHKNPDYFPDPEKFDPSRFEGNGPIPYTYVPFGGGPRMCPGKEYARLEI
LVFIHNVVRRFSWYKLHPNEDVIVDP

>CYP716A8 XP_002309057.1 P. trichocarpa
SDWEGKQEVSVFPLAKTYTFWLACRLFLSMEDPEEVQKFAKPFND
LAAGIISIPIDLPWTPFNRGVKASNVVHKELLKIIKQRKIDLAENKASPTQDILSHMLTTADDNGQCMKK
IDIADKILGLLVGGHDTASAAITFIVKYLAELPHVYNKLLEEQREIAKTKTPGELLNWEDIQRMRYSWNV
ACEVMRVAPPLQGAFREAMTEFNYAGFTIPKGWKLYWSANTTHKNPECFPEPENFDPSRFEGNGPAPYTF
VPFGGGPRMCPGKEYARLEILVFLHNLVKKFRWEKLLPKERIIVDP

>CYP716A3 XP_002324668.2 P. trichocarpa
LEWGNKAEVVVFPLAKSYTFELACRLFLSIEDPSHIARFSHPFNHITSGIFTIPIAFPGTPFNRA
IKATKLIRIELLAIIRQRKKDLAEGKASPTQDILSHMLLSNDANGQYMNEVEIADKIIALLLGAHDSTGT
ACTFIVKYLAEMPHIYEAVYKEQAEIIKSKAPGELLNWVDIQKMKYSWNVACETLRLSPPFIGNFKEAIK
DFTFNGFAIPKGWKASHFLTLYWSASSTHKNPEYFSEPEKFDPSRFEGKGPAPYTFIPFGGGPRMCPGNE
YARLEILVFMHNLVKRFKFERLVLDEKIVFDP

>CYP716A19 XP_002280969.1 V. vinifera
SSWNNKEEVTVFPLAKMFTFWLACRLFLSVEDPDHVEKLAEPFNEL
AAGIIALPIDLPGTSFNKGIKASNLVRKELHAIIKKRKMNLADNKASTTQDILSHMLLTCDENGEYMNEE
DIADKILGLLVGGHDTASATITFIVKFLAELPHVYDEVFKEQMEIAKSKAPGELLNWEDIPKMRYSWNVA
CEVMRLAPPVQGAFREAMNDFIFEGFSIPKGWKLYWSTHSTHRNPEFFPKPEKFDPSRFDGKGPAPYTYV
PFGGGPRMCPGKEYARLEVLVFMHNLVRRFKWEKLLPDEKIIVDP

>CYP716A6 XP_002325241.2 P. trichocarpa
SEWENKKVVVVFPLAKRFTFGLACSLFLSIEDPDHIAKLASPFNLVVSG
IFSIPIDLPGTPLSRAIKASTIIRTELFAIIKQRKKDLAKGKASPKQDILSHMLACDEKGAFMSELDIAD
TILALLASAHESTSAACAFIVKYLAELPLIYNAVYKEQMKISETKAPGDDLLNWNDIQNMTYSWNVIREV
LRLCPTFPNVREAIHDFDFNGFSIPKGWKAKYFPEPERFDPSRFEGTGPAPYTFVPFGGGPMMCPGQGFA

RLEMLIFMHNLVKRFKFDKFVAEEKIMFSP

>CYP716A4 ABC94483.1 A. annua
TEWDGMDQIVTHEVTQNFTFSLACKIFVSIEDPEEVKHLSGPFEKFAPGIFS
IPIDLPWTPLRRAIHAGNFIRKEIIAIIKQRKIDLADGKASPTQDILSQMLCDEESQNIAEADTADVIIG
LLIGGHDNASSTCAFIVKFLADLPEIYEGVLKEQLEIAKFKAPGELLNWEDLSKMKYSWNVACEVLRLAP
PLQGSFREAMTDFVYNGYSIPKGWKLYWSALSTHKNPEVFTEPQKLDPSRFDGKGPAPYTFVPFGGGPHM
CPGREYARLEILVFMHHLVIKYKWEKVIPNEQIIVNP

>CYP716A175 XP_008391096.1 M. domestica
EGWENKKEVEVFPLAKNYTFWLAARLFVSLEDSVEIAKLGDPFAVLAS
GIISMPLDFPGTPFYKAIKASNFIREELTKIIKQRKIDLAEGKASPTQDILSHMLLLCDEHGSHMKEHDI
ADKILGLLIGGHDTASATCTFIVKYLAELPHIYDEVYKEQMEVLSAKAPGDLLNWDDLQKMKYSWNVAQE
VLRLAPPLQGAFREALSDFVFNGFTIPKGWKLYWSANSTHKNAAYFPEPFKFDPTRFEGNGPAPYTFVPF
GGGPRMCPGKEYARLEILVFMHNLVKRFKWEKVLPDEQIVVDP

>CYP716A15 BAJ84106.1 V. vinifera
DSWDNRDEVIVFPLAKRFTFWLACRLFMSIEDPAHVAKFEKPFHVLAS
GLITVPIDLPGTPFHRAIKASNFIRKELRAIIKQRKIDLAEGKASQNQDILSHMLLATDEDGCHMNEMEI
ADKILGLLIGGHDTASAAITFLIKYMAELPHIYEKVYEEQMEIANSKAPGELLNWDDVQNMRYSWNVACE
VMRLAPPLQGAFREAITDFVFNGFSIPKGWKLYWSANSTHKSPECFPQPENFDPTRFEGNGPAPYTFVPF
GGGPRMCPGKEYARLEILVFMHNVVKRFKWDKLLPDEKIIVDP

>CYP716A17 BAJ84106.1 V. vinifera
DSWDNRDEVIVFPLAKRFTFWLACRLFMSIEDPAHVAKFEKPFHVLAS
GLITVPIDLPGTPFHRAIKASNFIRKELRAIIKQRKIDLAEGKASQNQDILSHMLLATDEDGCHMNEMEI
ADKILGLLIGGHDTASAAITFLIKYMAELPHIYEKVYEEQMEIANSKAPGELLNWDDVQNMRYSWNVACE
VMRLAPPLQGAFREAITDFVFNGFSIPKGWKLYWSANSTHKSPECFPQPENFDPTRFEGNGPAPYTFVPF
GGGPRMCPGKEYARLEILVFMHNVVKRFKWDKLLPDEKIIVDP

>CYP716A12 CBN88268.1 M. truncatula
THWDNKNEITVYPLAKRYTFLLACRLFMSVEDENHVAKFSDPFQLIAAG
IISLPIDLPGTPFNKAIKASNFIRKELIKIIKQRRIDLAEGTASPTQDILSHMLLTSDENGKSMNELNIA
DKILGLLIGGHDTASVACTFLVKYLGELPHIYDKVYQEQMEIAKSKPAGELLNWDDLKKMKYSWNVACEV
MRLSPPLQGGFREAITDFMFNGFSIPKGWKLYWSANSTHKNAECFPMPEKFDPTRFEGNGPAPYTFVPFG
GGPRMCPGKEYARLEILVFMHNLVKRFKWEKVIPDEKIIVDP

>CYP716A16 XP_021893541.1 C. papaya
SGWENKEEVKVFPLAKSYTFWIACRLFMSIEDPNHVERFAEPFHHLAS
GVISIPIDLPGTAFNRGIKASNFIRKELLKIIKQRKIDLGNGSASPTQDILSHMLLTSDESGQFMSELDI
ADKILGLLIGGHDTASAACTFIVKYLAELPHIYQKVYEEQMEIAKSKAAGEMLNWDDIQKMKYSWNVACE
VLRLAPPLQGAFRQALYDFIFDGFSIPKGWKLYWSANSTHKDPECFPEPEKFDPTRFEGNGPAPYTFVPF
GGGPRMCPGKEYARLEILVFMHNVIKRFKWEKVLPDEKIIVDP

>CYP76AH1 S4UX02.1 S. miltiorrhiza
KCSDSGRAVDIREAAFITTLNLMSATLFSSQATEFDSKATMEFKEIIEGVATIV
GVPNFADYFPILRPFDPQGVKRRADVFFGKLLAKIEGYLNERLESKRANPNAPKKDDFLEIVVDIIQANE
FKLKTHHFTHLMLDLFVGGSDTNTTSIEWAMSELVMNPDKMARLKAELKSVAGDEKIVDESAMPKLPYLQ
AVIKEVMRIHPPGPLLLPRKAESDQEVNGYLIPKGTQILINAYAIGRDPSIWTDPETFDPERFLDNKIDF
KGQDYELLPFGSGRRVCPGMPLATRILHMATATLVHNFDWKLEDDSTAAA

>CYP716A89 AJD25248.1 S. miltiorrhiza
DGWDNKSEVVVFPLAKNYTFWLACRLFLSVEDPAQVDKFAAPFNLLASG
LISIPIDLPGTPFNKGIKASAYIRKELVAIIKQRKADLADGKASPTQDILSHMLLTSNEDGKFMQESDIA
NKILGLLIGGHDTASSACTFIVKFLAELPQVYEGVYKEQMEIAKSKGEGELLSWEDLQKMKYSWNVACEV
LRLAPPLQGAFREALADFSFNGFSIPKGWKLYWSANSTHKNKEFFPEPEKFDPSRFEGSGPAPYTFVPFG
GGPRMCPGKEYARLEILVFMHHLVKRFKWEKMIPDEKIVVDP

>CYP716A48 BAP59949.1 O. europaea
DGWDNKNEVVVFPLAKRYTFWLACRLFVSVEDPAHVAKFADPFNELASG
LISIPIDLPGTPFHRAIKSSNFIRKELVSIIKQRKIDLAEGKASPTQDILSHMLLTSDESGKFMHELDIA

DKILGLLVGGHDTASSACTFVVKYLAELPEIYEGVYQEQMEIAKSKAPGELLNWDDIQKMKYSWNVACEV
LRLAPPLQGAFREAITDFMFNGFSIPKGWKLYWSANSTHRNSEFFPEPLKFDPSRFEGSGPAPYTFVPFG
GGPRMCPGKEYARLEILVFMHHLVKRFKWEKLIPDEKIVVDP

>CYP716A13 XP_006338129.1 S. tuberosum
SGWENKEQVVVFPLTKRYTFWLACRLFLSVEDPNHVAKFADPFDVLASGLISIP
IDLPGTPFNRAIKASIFIRKELVRIIKQRKIDLGEGKASSTQDILSHMLLTCDENGKFMGDLDIADKILG
LLIGGHDTASSACAFIVKYLAELPHIYQRVYTEQMEIAKSKGPGELLRWEDIQKMKYSWNVACEVLRLAP
PLQGAFREALSDFIFNGFSIPKGWKIYWSANSTHKSGEFFPDPEKFDPSRFEGSGPAPYTFVPFGGGPRM
CPGKEYARLEILVFMHHLVKRFKFEKIIPDEKIIVNP

>CYP716A36 XP_016474461.1 N. tabacum
SSWENKNQIEVFPLAKRYTFWLACRLFVSVEDPNHVAKFADPFNVLAS
GLISIPIDLPGTPFNRAIKASNLIRKELLLIIKQRKVDLAEGKASPTQDILSHMLLTSDENGKYMHELDI
ADKILGLLIGGHDTASSACTFILKYLAELPEIYEGVYKEQMEIAMSKSPGELLNWDDIQKMKYSWNVACE
VLRLASPLQGAFREAINDFIFNGFYIPKGWKLYWSANSTHKNPEYFPEPQKFDPSRFEGSGPAPYTFVPF
GGGPRMCPGKEYARLEILVFMHHLVKRFKWEKVIPNEKIVVDP

>CYP716A52v2 AFO63032.1 P. ginseng
SGWENKNEVVVFPLAKSYTFWIACKVFVSVEEPAQVAELLEPFSAIA
SGIISVPIDLPGTPFNSAIKSSKIVRRKLVGIIKQRKIDLGEGKASATQDILSHMLLTSDESGKFMGEGD
IADKILGLLIGGHDTASSACTFVVKFLAELPQIYEGVYQEQMEIVKSKKAGELLKWEDIQKMKYSWNVAC
EVLRLAPPLQGAFREALSDFTYNGFSIPKGWKLYWSANSTHINSEVFPEPLKFDPSRFDGAGPPPFSFVP
FGGGPRMCPGKEYARLEILVFMHHLVKRFKWEKVIPDEKIVVNP

>CYP716A50 BAP59951.1 C. arabica
SGWENKDQVKVFPLCKNYTFWIASRLFVSVEEPTEVAKLLEPFNVLASG
LISVPIDLPGTPFNRAVKASNQIRKMLVALIKQRKVDLAESKASPTQDIMSHMLTISDENGKFMHELDVA
DKILGLLIGGHDTASSACTFVIKFLAELPEIYEGVYKEQMEIVKSKAPGELLNWDDIQKMKYSWNVACEV
LRLAPPLQGAFREALADFMYNGFSIPKGWKIYWSANTTHRNPECFPEPQKFDPSRFEGSGPAPYTFVPFG
GGPRMCPGKEYARLEILVFIHHVVKRFRWEKIIPDEKIVVDP

>CYP716AL1 AEX07773.1 C. roseus
SGWENREQVEVFPLAKNYTFWLASRLFVSVEDPIEVAKLLEPFNVLASG
LISVPIDLPGTPFNRAIKASNQVRKMLISIIKQRKIDLAEGKASPTQDILSHMLLTSDENGKFMHELDIA
DKILGLLIGGHDTASSACTFIVKFLGELPEIYEGVYKEQMEIANSKAPGEFLNWEDIQKMKYSWNVACEV
LRLAPPLQGAFREALNDFMFHGFSIPKGWKIYWSVNSTHRNPECFPDPLKFDPSRFDGSGPAPYTFVPFG
GGPRMCPGKEYARLEILVFMHNLVKRFKWEKIIPNEKIVVDP
